# Novel approach for identification of influenza virus host range and zoonotic transmissible sequences by determination of host-related associative positions in viral genome segments

**DOI:** 10.1186/s12864-016-3250-9

**Published:** 2016-11-16

**Authors:** Fatemeh Kargarfard, Ashkan Sami, Manijeh Mohammadi-Dehcheshmeh, Esmaeil Ebrahimie

**Affiliations:** 1 Department of Computer Science and Engineering, School of Electrical and Computer Engineering, Shiraz University, Shiraz, Iran; 2 School of Animal and Veterinary Sciences, The University of Adelaide, Adelaide, Australia; 3 School of Medicine, Faculty of Health Sciences, The University of Adelaide, Adelaide, Australia; 4 Institute of Biotechnology, Shiraz University, Shiraz, Iran; 5 School of Information Technology and Mathematical Sciences, Division of Information Technology, Engineering and the Environment, University of South Australia, Adelaide, Australia; 6 School of Biological Sciences, Faculty of Science and Engineering, Flinders University, Adelaide, Australia

**Keywords:** Association rule mining, Host range of influenza, Detecting hot spots

## Abstract

**Background:**

Recent (2013 and 2009) zoonotic transmission of avian or porcine influenza to humans highlights an increase in host range by evading species barriers. Gene reassortment or antigenic shift between viruses from two or more hosts can generate a new life-threatening virus when the new shuffled virus is no longer recognized by antibodies existing within human populations. There is no large scale study to help understand the underlying mechanisms of host transmission. Furthermore, there is no clear understanding of how different segments of the influenza genome contribute in the final determination of host range.

**Methods:**

To obtain insight into the rules underpinning host range determination, various supervised machine learning algorithms were employed to mine reassortment changes in different viral segments in a range of hosts. Our multi-host dataset contained whole segments of 674 influenza strains organized into three host categories: avian, human, and swine. Some of the sequences were assigned to multiple hosts. In point of fact, the datasets are a form of multi-labeled dataset and we utilized a multi-label learning method to identify discriminative sequence sites. Then algorithms such as CBA, Ripper, and decision tree were applied to extract informative and descriptive association rules for each viral protein segment.

**Result:**

We found informative rules in all segments that are common within the same host class but varied between different hosts. For example, for infection of an avian host, HA14V and NS1230S were the most important discriminative and combinatorial positions.

**Conclusion:**

Host range identification is facilitated by high support combined rules in this study. Our major goal was to detect discriminative genomic positions that were able to identify multi host viruses, because such viruses are likely to cause pandemic or disastrous epidemics.

**Electronic supplementary material:**

The online version of this article (doi:10.1186/s12864-016-3250-9) contains supplementary material, which is available to authorized users.

## Background

Influenza A is a virus related to the Orthomyxoviridae family of negative sense, single-stranded, segmented RNA viruses. This virus includes eight functional protein segments: HA (hemagglutinin), NA (neuraminidase), NP (nucleoprotein), M (two matrix proteins, M1 and M2), NS (two distinct non-structural proteins, NS1 and NS2), PA (RNA polymerase and PA-X), PB1 (RNA polymerase and PB1-F2 protein), and PB2 (RNA polymerase) [1].

The natural host of influenza virus is aquatic birds though they are capable of infecting a number of other host species, including swine and humans [2]. Influenza A evolves through different mechanisms, including point mutations and gene reassortment causing antigenic drift and antigenic shift respectively [3]. Interactions occur between viruses of different lineages. The segmented structure of the virus facilitates gene reassortment when viruses from different hosts simultaneously infect a single cell [4]. The reassortment of genetic material between viruses with different host origins can significantly alter antigenic sites [5]. By this mechanism, novel viruses may enter the human population that lacks previous immunity, potentially causing the emergence of pandemics or disastrous epidemics [6].

Three global pandemics emerged in the twentieth century by antigenic shift between viruses with different hosts. Reassortment of avian viruses with circulating viruses in mammalian hosts such as human or swine caused the 1918 H1N1 pandemic [7]. The 1957 H2N2 pandemic was the consequence of a reassortment of five human H1N1 segments and avian segments encoding the viral surface proteins and the PB1 protein. Similarly, the 1968 H3N2 pandemic involved a reassortment of avian segments encoding hemagglutinin and PB1 [8]. The viral genome of the 2009 H1N1 pandemic had a more complex history involving triple reassortment between hosts which mixed segments of human H3N2 (PB1), avian influenza A virus (PA, PB2), and classical North American swine influenza A virus (HA, NP, NS), [9, 10]. This genetic reassortment allowed the virus to infect human, swine, and birds and, in addition, it acquired the life-threatening ability to transmit from human to human without the need of intermediate swine or birds.

Molecular factors governing host range and the possibility of human-to-human transmission are largely unknown. Determination of these factors is vital for improvement of antiviral treatment strategies, developing new vaccines targeting the risk regions, and early detection of potential pandemic strains. The roles of some segments in host recognition specificity have been investigated recently. Matrosovich et al. [11] showed that positions 138, 190, 194, 225, 226, and 228 are highly conserved in the HA amino acids of avian viruses, whereas point substitutions occur in these positions in viruses infecting humans. Although, the HA sequence has a key influence on host determination, the PB2 polypeptide also plays a critical role. In particular, the residue 627 of the PB2 polypeptide is highly important in host identity. Additionally, other segments such as, PB1, NP and PA contribute to host range, and compatibility between these four polypeptides is also important. Gene sequence analysis of M1 and M2 proteins shows respectively 3 and 7 sites with amino acids specific for human or avian variants [12]. In 2009, Allen et al. [13] found sixteen positions on NS1, NS2, PA, NP, M1 and PB2 proteins which were related to human host range.

Due to the high level of complexity, the major challenge is finding an approach which can consider contributions of all segments and different host specificities to elucidate this biological phenomenon. Sherif et al. [14] used several computational methods to identify genetic signatures characteristic of the HA gene of swine, human and bird viral strains. Application of supervised data mining has opened a new avenue for better understanding of diseases, gene expression, protein behavior, drug design and performance, and molecular marker discovery [4, 15–25]. In particular, association rule mining is an effective method that has the potential to discover interesting and previously hidden relationships between items in a dataset [26]. The technique can be applied to gene expression data [27], protein bioinformatics [24] and medical data [28]. In our previous study, we used association rule mining to identify and predict pandemic H1N1 influenza [29].

Current understanding of reassortment patterns between viral host groups which eventuate in emerging pandemic viruses is very limited. In all previous studies, factors have been evaluated for a single host. However, some viral strains infect more than one host. Identification of host range of an influenza sequence is a major challenge. It is possible that the influenza sequence has a completely different origin from the host which it is isolated. To address this issue, based on literature surveys (Additional files 1 and 2 include the references of whole strains), we constructed a new dataset containing all segments of influenza A viruses showing a range of hosts, rather than one single host. Some of the sequences belong to one class while others were held by two or three classes. In fact, we have three labels (human, avian, and swine) and our sequences were categorized into seven groups: human, avian, swine, human-avian, human-swine, avian-swine, and human-avian-swine. Therefore, our datasets were multi-labeled datasets. In order to effectively identify host range in our new dataset, different rule-base classifications were applied to extract association rules. Association rule mining techniques provide the best potential solution to extract mutation/reassortment spots with important influence on host determination. We also used the distinct amino acid residues between viral genomes of different hosts to generate combinational rules between all segments. This allowed development of predictive models to provide a novel strategy for recognition of future pandemic influenza with a broad host range.

## Results and discussion

We applied CBA, Ripper, C4.5, and decision tree algorithms for rule extraction in identifying host range of influenza sequences for each protein sequence of influenza in different iterations. The final association rules cover entire dataset. We specified the minimum support for frequent itemsets to be 0.5% and the minimum confidence for association rules to be 80%.

Table 1 illustrates some of the extracted rules of the first iteration on HA protein sequences. These rules suggest the most informative positions among the three different host range groups. For example, rule 1 in Table 1 declares that the content at position 444 of HA sequences is ‘D’ in the 30.861% of whole sequences whose host is avian. As a matter of fact, 1/3 of avian sequences have ‘D’ value at this position. As another example, where position 153 is ‘T’, 256 is ‘N’, 140 is ‘S’, and 405 is ‘V’, almost 21% of the sequences are considered human. Also, if position 177 is ‘N’ and position 9 is ‘M’ then more than 12% of sequences will assign to the swine category.

**Table 1:** Part of extracted rules of influenza A viral strain of HA protein to identify host ranges

Class	Rule	Support	Confidence	Algorithm
Avian	Att444 = D	30.861%	100%	CBA
Avian	Att540 = R and Att9 = −	22.255%	100%	CBA
Avian	Att540 = R and Att10 = M	21.513%	100%	CBA
Avian	Att117 = N and Att15 = L	17.953%	100%	CBA
Avian	Att517 = A and Att121 = G and Att286 = I and Att433 = D and Att451 = E	11.869%	100%	DT
Human	Att153 = T and Att265 = N and Att140 = S and Att405 = V	21.068%	100%	DT
Human	Att176 = K and Att9 = −	8.605%	100%	CBA
Human	Att194 = K and Att9 = K	6.973%	100%	CBA
Human	Att571 = I and Att13 = I	5.341%	100%	CBA
Human	Att173 = L and Att13 = I	4.303%	100%	CBA
Swine	Att177 = N and Att9 = M	12.908%	100%	CBA
Swine	Att163 = R and Att9 = M	11.424%	100%	CBA
Swine	Att223 = T and Att11 = A	6.825%	100%	CBA
Swine	Att448 = I	5.638%	100%	CBA
Swine	Att52 = I and Att9 = M	5.045%	100%	CBA

In the next iteration, first we eliminated sequences covered by rules of iteration 1. Then we reapplied CBA, Ripper, C4.5, and decision tree algorithms to the remaining datasets, such that several rules with new support and confidence were extracted. These rules represent the most discriminative spots between various hosts, whereas our original aim was to find the most informative sites that are common within class and varied between classes. Additional files 3, 4, 5, 6, 7, 8, 9, 10, 11, 12, 13 and 14 illustrates the details of extracted rules for a range of hosts and in different segments.

In order to satisfy our main goal entirely, we utilized these discriminative points as features of a new dataset. Potential distinctive points of each polypeptide provided a new opportunity to put these points together for building powerful informative and discriminative rules. Remarkably, these new rules have high support, common within a class and showing variation between different hosts, thus achieving one of our major aims. The details of this novel approach are explained in Additional file 15 using illustrative examples.

Table 2 shows a selected set of integrated rules to identify host range. It appears that most rules are combinations of informative positions of several segments. The first rule indicates that position 444 of HA plays a significant role in identification of the avian host. The second rule reveals that both position 14 of HA and 230 of NS1 influence host variety. The remaining rules of Table 2 display the significant positions of diverse segments in distinguishing avian, human and swine hosts. This table indicates that most segments of influenza participate in effective host range. Comparison between these rules and the rules of each segment, suggest that the integrated rules are more concise and precise. In other words, they have high support and confidence. The complete integrated rules are illustrated in Additional file 16. In order to evaluate the proposed distinctive points of each polypeptide in this study in comparison to previous reports, we searched other studies which identify amino acids specific for these viruses. In most such studies, the number of viruses was limited (less than 10) and all were from a restricted geographical area. In contrast, our large scale analysis considered 674 viral strains from different hosts across the entire world. Nonetheless, we identified 50 positions in these previous limited studies which corresponded to our discriminative positions. Table 3 illustrates the characteristics of these positions for each segment. Additional file 16, describes the detail of these positions in the cited references and the current study.

**Table 2:** The selective set of integrated rules extracted from whole segments of influenza (protein sequences) A strain to identify host ranges

Host	Rule	Support	Confidence
Avian	HA_Att444 = D	30.861%	100%
Avian	NS1_Att230 = S and HA_Att14 = V	16.320%	100%
Avian	NS1_Att229 = E and HA_Att117 = N	15.282%	100%
Avian	NA_Att85 = L	14.688%	100%
Avian	PB1_Att257 = T and HA_Att121 = D	14.392%	100%
Avian	NA_Att364 = Y and HA_Att117 = N	12.463%	100%
Avian	PB1_F2_Att76 = V and HA_Att14 = V	10.979%	100%
Human	HA_Att194 = T and HA_Att16 = L	8.754%	100%
Human	M2_Att66 = A	6.231%	100%
Human	NA_Att387 = L and HA_Att9 = K	6.083%	100%
Human	PB2_Att134 = A	5.786%	100%
Human	NS2_Att14 = V and HA_Att13 = I	5.045%	100%
Human	PB1_F2_Att84 = S and HA_Att13 = L	3.412%	100%
Human	NS1_Att59 = L and HA_Att14 = F	3.412%	100%
Swine	HA_Att240 = S and HA_Att222 = S	17.211%	100%
Swine	NS1_Att125 = I and HA_Att9 = M	12.463%	100%
Swine	M1_Att207 = S and HA_Att9 = M	9.347%	100%
Swine	PB1_Att12 = V and HA_Att9 = M	9.050%	100%
Swine	NA_Att394 = I and HA_Att177 = N	8.309%	100%
Swine	PA_Att208 = K	6.083%	100%
Swine	NP_Att431 = I and HA_Att9 = M	5.341%	100%

**Table 3:** Similarities and novelties in number of critical positions in determining influenza host range. (This comparison is between previous studies and the present study. In this study associative classification rule mining was used. The previous studies are based on laboratory techniques.)

Segment	Number of common position	Number of new positions in this study	Common Position between current study and biological studies
HA	6	46	13, 129, 158, 194, 222, 224, 226
M1	11	25	15, 59, 95, 115, 116, 121, 167, 181, 239
M2	17	16	11, 14, 16, 18, 20, 21, 27, 28, 31, 44, 54, 55, 61, 78, 82
PB1	9	34	152, 157, 211, 339, 375, 397, 581, 654
PB2	3	34	134, 153
NS1	6	50	91, 125, 127, 213
NS2	1	24	40
NA	1	59	-
PA	4	51	55, 57, 409
NP	1	60	377
PA-X	0	36	-
PB1-F2	0	51	66

A significant highlight of this study is finding associative positions between different segments (co-occurrence of mutation/reassortment) whereas previous research only detected a single divergence in sequences in one experiment.

## Conclusions

In this research, in addition to the standard host categories of influenza i.e. human, avian, and swine, we investigated viruses that belong to more than one class. Thus we have four additional classes: human-swine, human-avian, avian-swine, and human-avian-swine. These classes evolved by breaking host barriers via reassortment between viral strains. We applied rule-based classification algorithms and a multi-label learning method to identify the statistically significant points of the influenza viral genomes relating to hosts range. Descriptive rules were successfully identified that facilitated specific detection of the viral hosts. These rules potentially revealed undiscovered important sites of novel viral protein sequences likely to encounter low levels of host antibody and immunity.

In conclusion, genome comparisons of viruses originating in avian, human, swine, human-avian, human-swine, avian-swine, and human-avian-swine hosts using integrated rules of polypeptide sequences represent the evolutionary pathways of bypassing host barriers. The results provide new information for studying the mechanisms of pandemic viral infection and replication in various hosts. Here, informative combined class association rules with high statistical support and confidence were produced that will improve prognosis. Pattern analysis, including all segments of the influenza viral genome, was performed to extract the most important and distinctive positions. Fast detection of pandemic influenza can improve flu antiviral strategies, reduce species mortality, and prevent financial losses. Unravelling the discriminative factors of host range in this study provided new insights into the underlying mechanisms of evolution of pandemic influenza strains. Recognition of these informative sequence positions offers novel markers for reliable detection of potential new pandemic viruses and improving the efficiency of vaccines.

## Methods

**Fig. 1:**
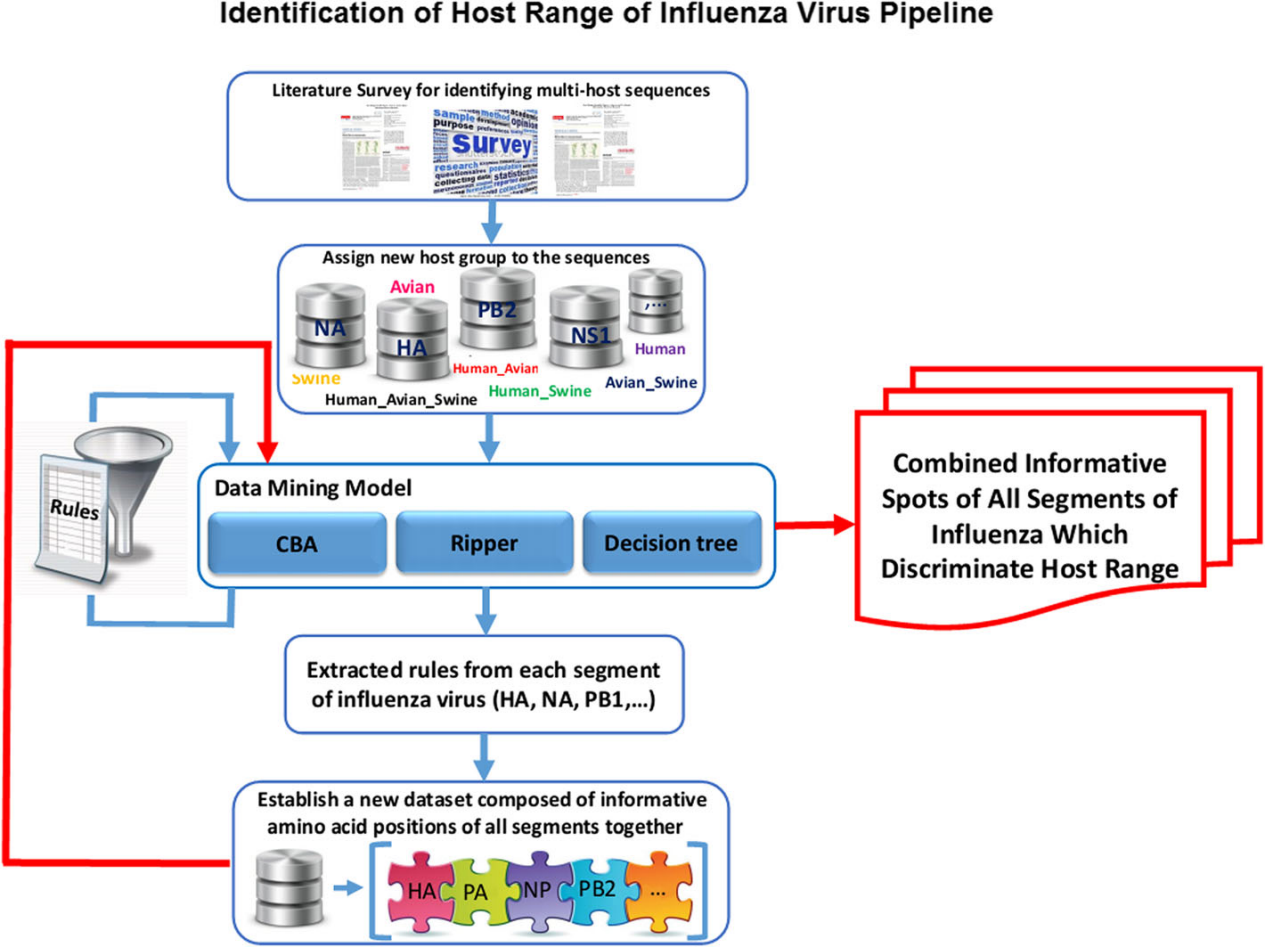
The pipeline of identification influenza host ranges based on rule discovery methods and integration of significant spots on each segment

The following steps were taken to discover sequence based markers and for identifying models of host range: 1) Sequences were collected based on related literature and multiple host group were assigned to some of these sequences. 2) Multiple sequence alignments. 3) Informative sites (positions) in every host group in each segment were detected by applying different rule base classification and multi-labeled learning methods. 4) A new dataset was constructed composed of informative amino acid positions of all segments together. 5) Class association rules were extracted in each host group of the new dataset. Figure 1 represents a schematic view of identification of host ranges of the influenza pipeline.

### Data collection and first dataset construction

A dataset containing 12 proteins was generated to identify combinational molecular factors of all segments for detecting hosts range. Only complete sequences with protein sequences for all segments were used in this research. The sequences were divided into two parts: the sequences belonging only to one of the avian or human or swine categories (three classes) and the sequences belong to two or three of these classes i.e. they belonged to both human and avian, human and swine, avian and swine, or human and avian and swine (four classes). Most of the sequences were downloaded from the Influenza Research Database [30]. A small number of sequences were downloaded from other Influenza data repositories such as the NCBI Influenza Virus Resource [31] and the Global Initiative on Sharing All Influenza Data databases [32].

**Fig. 2:**
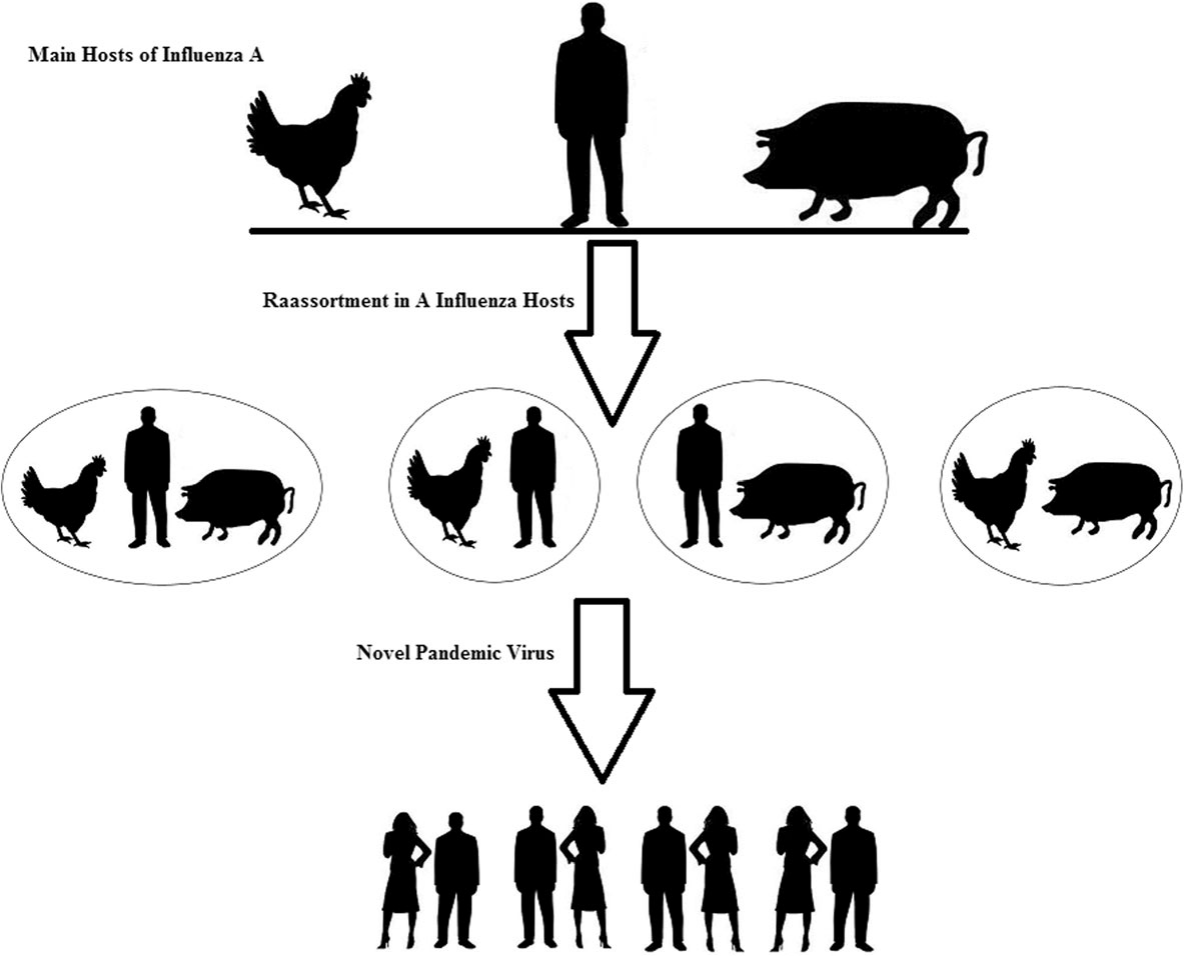
Schematic view of reassortment between main hosts of influenza A that cause novel pandemic viruses

To the best of our knowledge, none of the repositories has provided multiple hosts of a viral strain, to run supervised data mining for rule discovery, and it was necessary for us categorize sequences based on the similarity of their segments and infection range of hosts. If a viral strain infected one host and also it had one or more segments similar to another host, we classified it as two-hosted. Table 4 provides more information about the dataset. Figure 2 represents a schematic view of reassortment between influenza A strains isolated from the main hosts that causes novel pandemic viruses.

**Table 4:** The number of sequences of influenza A used for host range identification in the current study (These sequences were obtained from a literature review and separated based on hosts and segments)

Host/segment	PB2	PB-1	PB1-F2	PA	PA-X	HA	NP	NA	M1	M2	NS1	NS2
Avian	65	71	42	68	76	118	77	80	86	72	88	77
Human	64	69	41	68	74	108	74	118	72	62	74	70
Swine	48	52	19	52	53	81	65	57	65	44	67	53
Avian-Swine	17	17	9	19	22	18	33	13	20	17	22	20
Human-Avian	106	107	68	117	112	159	118	126	126	111	133	113
Human-Swine	80	91	13	78	85	136	100	122	101	97	86	82
Human-Avian-Swine	46	47	20	47	52	55	51	71	52	47	50	47
Total number	426	454	212	449	474	675	518	587	522	450	520	462

Here we explain some examples to clarify our categorization procedure:

- From April 2013, newly emerged 2013 influenza of H7N9 subtype has infected 33 and killed 9 people in China. In this re-assorted virus, HA and NA genes originated from Eurasian avian influenza viruses while the remaining segments are closely related to avian H9N2 influenza [33]. We considered this viral strain as belonging to both human and avian categories.
- All protein components of the swine virus that emerged in the United States in 2003 had 97 to 98% sequence similarity to two H3N2 isolates, A/turkey/NC/16108/03 and A/turkey/MN/764/03 [34]. Therefore, we assumed that this viral strain belongs to both avian and swine categories.
- Antigenic and genetic features of the A/Hong Kong/1774/99 strain, which was isolated from a young child with mild influenza, are similar to H3N2 viruses circulating in pigs in Europe during the 1990s [35]. This viral strain was allocated to both human and swine categories.
- Antigenic and genetic analysis of the 1994 H1N2 influenza A viruses of pigs in United Kingdom suggested that the RNA segments encoding PB2, PB1, PA, NP, M and NS were related closely to those of avian viruses [36] whereas the HA segment was similar to human H1N1 viruses. Therefore, we assigned these viral strains to three host categories: human, avian, and swine.

### Multiple sequence alignment

Multiple sequence alignment is required in this study for universalization of positions [14]. MUSCLE is perhaps the most well-known multiple alignment software for protein and nucleotide sequences [37]. MUSCLE is capable of both better average accuracy and speed compared with various other multiple alignment tools such as CLUSTALW [38] or T-Coffee [39] by maximizing iterations and diagonal optimization. Using MUSCLE, the variable ‘maximum iteration’ and ‘maximum memory in MB’ were set at 2000 and 3000 MB, respectively. All subtypes in each segment were aligned to obtain common numbering. In fact, whole sequences of the HA segment, regardless of any subtype, were aligned to each other. The same operation was done on other segments (NA, PA, NS1, and etc.)

After sequence alignment, data were stored in a relational table, with a set of attributes featuring the amino acid at each position in a sequence (for example Att20 describes the amino acid at position 20 of the sequence). In the case of the CBA tool, data were converted into C4.5 format (*.data, *.names files).

### Method description

#### Multi-label learning

Multi-label learning is a kind of supervised learning where multiple target labels are assigned to each instance, and then used to predict a set of class labels for a new instance [40]. In this study, the set of class labels is human, avian, or swine, where each instance can belong to one or more classes in this set. There are two main methods for coping with the multi-label learning problem: Simple problem transformation methods and Simple algorithm adaptation methods [31]. Simple problem transformation methods transform the multi-label problem into several binary classification problems and then deal with them one by one. Simple algorithm adaptation methods expand particular learning algorithms in order to handle multi-label data directly [31]. There are several simple problem transformation methods for multi-label classification: Label Powerset (LP), Binary Relevance (BR), Ranking by Pairwise Comparison (RPC), Calibrated Label Ranking (CLR). We have utilized Label Powerset (LP) and Binary Relevance (BR) in this study. The label powerset (LP) transformation creates one binary classifier for every unique label combination. In this study, we have seven labels instead of three labels: human, avian, swine, human-avian, human-swine, avian-swine, and human-avian-swine. Therefore, the new transformed problem is a single label classification task.

Nevertheless, one of the most popular transformation approach is Binary Relevance (BR) that decompose a multi-labeled dataset into k datasets (k = total number of classes) and trains an independent binary classifier on each of these datasets. All of these datasets consist of the same number of instances as the original data, while each dataset considers only one label and, if the instances belong to that label, the class label will be positive. If not, the class label is negative [40]. Therefore, as we have three class labels (human, avian, and swine), our dataset of each segment decomposes to three binary labeled datasets. Hence, we have 36 binary labeled datasets from which to extract rules. In our analyses, Binary Relevance (BR) produces more accurate rules and high support and confidence while Label Powerset (LP) was more suitable for balanced and huge datasets.

#### Association rule

The association rule model discovers rules where a set of items is associated with each other. For instance, a rule could specify a certain product that was frequently found in combination with other products. The rules were extracted from some large and frequently-occurring dataset. An itemset is frequent if the number of its occurrences exceeds a specified minimal support criterion. Support and confidence determine the accuracy of the rules. The support of the rule is the number of transactions that contain that rule, while confidence is the number of situations where the rule is correct, relative to the number of situations that the rule is possible [14]. Here, each protein sequence represents a transaction (T) and where amino acids such as A, R, N, D, C, Q are items (I). All the sequences construct a D set. Y set include three host groups. An association rule is a concept of the form X ⇒ Y, where *X* ⊂ *I* that holds in the transaction set D with Confidence if c % of transactions in D that contains X also contains Y. The rule X ⇒ Y has support in the transaction set D if s % of transactions in D contains X∪Y in D set [41].

#### CBA algorithm

CBA is an integrative algorithm which has the power of both classification and association rule. This integration was done by mining class association rules (CARs). To make proper association rules for the classification, the associative classifier focuses on a unique subset of association rules, i.e. those rules resulted to class variables only, i.e. the so-called class association rules (CARs). Thus, only rules of the form A ⇒ c_i_, where c_i_ denotes a possible class, are generated [42].

CBA has 2 parts:

1. A rule generator (called CBA-RG), which is based on algorithm Apriori for finding association rules. The CBA-RG algorithm generates all frequent *rule items* by making multiple passes over the data [43].
2. A classifier builder (called CBA-CB). The CBA-CB algorithm builds a classifier using CARs. To produce the best classifier out of the whole set of rules, a minimum number of rule sets would be selected to cover the training dataset and minimize the lowest error rate [44].

#### Ripper algorithm

Ripper (Repeated Incremental Pruning to Produce Error Reduction) is a propositional rule learner, which is an optimized version of IREP. The algorithm consists of two phases. In the first phase, a rule set is built by repeatedly adding rules to an empty rule set until no positive examples exist, or the error rate > = 50%. Rules are formed by adding antecedents greedily (or conditions) to the rule when the rule is perfect (i.e. 100% accurate). After a rule set is constructed, each rule is pruned incrementally and any final sequences of the antecedents are pruned. In the second phase, an optimization is executed on the rule set in order to decrease its size and improve its fit to the training data [45].

#### Decision tree

A Decision tree is an uncomplicated representation intended for classifying instances. The purpose is to construct a model which predicts the value of a target variable according to numerous input parameters. In these tree structures, class labels are represented by leaves and branches symbolize conjunctions of features which result in those class labels. A tree is usually “learned” through dividing the original set into subsets according to an attribute value test. This process is replicated upon every taken subset in the recursive approach called recursive partitioning. The recursion is finished when the subset of a node has all the identical values of the target feature, or when additional division does not add more value to the predictions [46].

There are many specific Decision-tree algorithms. Notable ones are: ID3, C4.5, and CHAID [23, 47]. We used C4.5 for extracting rules. At every node of the tree, C4.5 selects the attribute of the data that most properly divides its set of instances into subsets enriched in one class or the other. The division measure is the normalized information gain (difference in entropy). The feature with the highest normalized information gain is taken to build the decision. This process is replicated on the smaller subset [48]. In this study, integration class association rules were produced based on informative markers that are the same in a category and different between various hosts. For rule extraction in detecting host range, the following steps were undertaken:

1. We applied CBA algorithm (an integrated classification and association rule mining [49], Ripper [45], and C4.5, as well as decision tree algorithms (rule based classification algorithms) [48] on every protein sequence. Each classifier prunes some of the intermediate rules to come up with a generalized model that describes the whole dataset. In this study we are mainly interested to find out the rules themselves. Deploying different classifiers will lead to larger set of descriptive rules. In addition, intersection of the rules presents a robust set of rule sets which have been identified by the methods. Therefore, we have applied different classification approaches to extract whole potential hotspots. We used the industrial version of these algorithms such as J48 and Jrip via RapidMiner software [50]. We assigned the minimum support for frequent itemsets to be 0.5% and the minimum confidence for association rules to be 80%.
2. In order to obtain whole association rules of datasets, the “under sampling” technique was applied. The sequences were covered by extracted rules of first step have eliminated.
3. The discovered rules of previous steps demonstrated statistically significant variations between different host classes. We suggested a new and creative approach. A new dataset was constructed with these points. In this new dataset, potential distinctive sites were assumed as features of the new dataset. In fact, the previous steps identify valuable sites and can perform as a feature selection method. Because, we encountered a multi-labeled dataset and our solution was based on the Binary Relevance (BR) method, we have constructed a new dataset for each label. The new datasets consisted of potential distinguished points of every 12 proteins. The number of instances is the same in each new dataset, but the number of features is 403, 333, and 287 for human, avian, and swine, respectively. The number of features in the new datasets was almost 10% of all features in whole segments. In order to achieve rules with high supports and confidence, step 1 was applied on new datasets. Extracted rules were combinations of different segments. Additional file 15 represent the full version of these rules. Additional file 17 explains the method in detail.

## Additional files

Additional file 1: Includes whole accession numbers of sequences used in the current study. (DOCX 37 kb)
Additional file 2: Includes whole references of strains that were used in this study. (DOCX 72 kb)
Additional file 3: Table S1. Listing the rules extracted from HA protein of influenza A in identification of host ranges. (DOCX 21 kb)
Additional file 4: Table S2. Listing the rules extracted from M1 protein of influenza A in identification of host ranges. (DOCX 17 kb)
Additional file 5: Table S3. Listing the rules extracted from M2 protein of influenza A in identification of host ranges. (DOCX 18 kb)
Additional file 6: Table S4. Listing the rules extracted from NA protein of influenza A in identification of host ranges. (DOCX 22 kb)
Additional file 7: Table S5. Listing the rules extracted from NP protein of influenza A in identification of host ranges. (DOCX 20 kb)
Additional file 8: Table S6. Listing the rules extracted from NS1 protein of influenza A in identification of host ranges. (DOCX 20 kb)
Additional file 9: Table S7. Listing the rules extracted from NS2 protein of influenza A in identification of host ranges. (DOCX 20 kb)
Additional file 10: Table S8. Listing the rules extracted from PA protein of influenza A in identification of host ranges. (DOCX 21 kb)
Additional file 11: Table S9. Listing the rules extracted from PA-X protein of influenza A in identification of host ranges. (DOCX 21 kb)
Additional file 12: Table S10. Listing the rules extracted from PB1 protein of influenza A in identification of host ranges. (DOCX 20 kb)
Additional file 13: Table S11. Listing the rules extracted from PB1-F2 protein of influenza in identification of host ranges. (DOCX 20 kb)
Additional file 14: Table S12. Listing the rules extracted from PB2 protein of influenza in identification of host ranges. (DOCX 22 kb)
Additional file 15: Tables S13–S15. Which contain integrated rules based on whole segments of influenza to identify human, avian, and swine hosts. (DOCX 20 kb)
Additional file 16: Table S16. Listing the properties of some of the critical (informative) spots in the determination of influenza host ranges, extracted by associative classification rule mining (in this study), which correspond with similar position in other studies. (DOCX 31 kb)
Additional file 17: Gives details of the developed approach in this study in increasing the support and accuracy of prediction and generation of more informative rules through making secondary datasets. The components of secondary datasets are discovered spots at the first run of rule discovery instead of sequence features. We extracted combinational association rules from several tables to gain rules with higher support and confidence. (DOCX 37 kb)

## Abbreviations

BR: Binary relevance
CBA: Classification based on associations
CLR: Calibrated label ranking
IREP: Incremental reduced error pruning
LP: Label powerset
Ripper: Repeated incremental pruning to produce error reduction
RPC: Ranking by pairwise comparison
