# Supplementary material for: Novel approach for identification of influenza virus host range and zoonotic transmissible sequences by determination of host-related associative positions in viral genome segments
Source: BMC Genomics. 2016 Nov 16;17:925. doi: 10.1186/s12864-016-3250-9 (PMC5112743; doi:10.1186/s12864-016-3250-9)
Supplement: Additional file 1: — Includes whole accession numbers of sequences used in the current study. (DOCX 37 kb) [file 12864_2016_3250_MOESM1_ESM.docx]

**Accession number of HA segment:**

CY034198,DQ099759,DQ099758,CY028700,CY029937,CY028708,CY033169,AB450550,EF541406,EU118132,AB450560,AB450559,AB450556,AB450555,AY649382,DQ083551,AB450558,AB450562,CY014296,HM172095,HM172075,CY028993,GQ122386,GQ122389,GQ122387,GQ122388,AY830774,EF670482,DQ080022,AY950230,AY653200,AY684706,AY950231,DQ997219,AY770079,AY950232,AY950233,AY950234,FJ610094,AB558473,GU052690,CY014717,GU052675,EU743019,AB558456,M30122,AB292664,CY014694,D90308,CY130086,CY006003,AB669139,AB288845,GU052203,CY014679,D90306,AF285885,CY101482,M25290,CY014751,M25286,CY006030,M25283,AB295611,GU052381,AF290436,D90302,AF290437,AF290438,FJ200415,FJ200423,EU296613,AB434360,JN244246,AB434344,CY101126,JQ070800,JN638733,JN655537,JN866186,JQ070768,JQ290172,JQ290188,JQ070776,JQ070784,JN992750,JQ070792,GU937743,EU399751,JX899803,CY067678,CY067670,CY024818,GQ921322,AY648287,EF470587,AY611524,AY646078,EU684261,CY127253,CY014786,M31689,AB269693,AF202247,AF202252,JN244238,GU053110,AY999981,CY022653,CY025125,CY025117,AB558261,GU060482,HQ244415,AY338460,AY338458,AY338457,AB438941,EF015551,AY338459,FN386467,HQ244407,GU052938,CY022677,JQ906576,EPI439486,AGJ72861,EPI440701,KC885956,AGJ51953,EPI439502,EPI440685,EPI440693,EPI439507,KC853766,AGI60301,M24457,M24458,GU186777,CY014992,CY087824,CY014663,CY130054,D90305,AB295601,DQ067444,AJ404626,AJ404627,AY664674,JF715006,JQ356874,KC464598,JQ356873,AY664667,AY664671,AB256746,AY664666,D90304,CY087776,CY014659,CY130046,AB289343,AB669140,D90307,CY130078,GU052216,AB288334,CY110941,CY110949,AF250479,AY703832,CY087752,AB296072,CY130030,AB278600,D90303,EU182253,AF474029,AJ410526,AJ410532,AJ410536,AB271115,AF091313,AF091312,CY014627,AB271113,AY619961,AF091309,AF091311,GU086065,GU086057,GU086073,JX860694,JX860678,JX963591,FJ415610,JX963605,JX860686,JN375119,JN375120,JN375121,JN375122,JN375123,JN375124,JN375125,JN375126,JN375127,JN375128,CY116380,CY010580,AF091316,U72667,CY025253,CY022986,AF091308,EU139823,EU296607,AB434336,AB434328,EU296599,AB434320,EU296601,EF101749,EU296603,FJ688266,EU296605,AB434304,AB434312,AB294217,CY057067,CY057068,CY057071,HM568000,HM567984,HM567968,HM567976,JN375109,JN375110,JN375111,JN375112,JN375113,CY057058,CY097781,JN617974,GQ457506,GQ168650,GQ232064,CY061805,JN375103,JN375104,JN375102,CY057039,CY057063,CY057052,CY057045,CY057060,HM568040,CY057040,CY057061,JN375106,JN375105,JN375107,CY041613,CY057950,CY057035,CY057036,CY057037,CY057038,CY057041,CY057048,CY057049,CY057050,CY057053,CY057054,CY057055,CY057056,CY057057,CY057059,CY057064,CY057065,CY057069,CY057070,FJ984355,GQ160556,GQ117082,GQ117086,GQ168628,FJ984364,FJ984360,CY061733,CY061773,CY061789,CY061797,CY061725,AB434408,AF091306,CY044365,CY130118,CY039991,AB434400,CY022341,CY022397,CY024933,U72668,U72666,AF222036,AF222026,AF222027,EU502884,GU086049,DQ058215,GU086017,GU086025,GU086009,GU086033,GU086041,AF222035,AF222032,CY020693,CY020701,CY020709,KF042153,KF042152,EU373736,EU717849,EU148404,EU148444,EU717853,CY020645,DQ862001,CY016899,DQ862002,CY020653,EU148372,EU148380,EU148396,EU277833,EU148356,EU148388,EU148436,EF619980,DQ407519,EU148364,CY016939,CY016947,CY016907,DQ838517,DQ838516,CY016284,CY017027,CY017179,CY016923,CY016931,CY016276,CY016300,CY021389,CY020677,DQ861999,CY020669,DQ862000,CY016292,CY020661,AY737296,AY737289,AF046096,AF028709,AF046099,AF057291,GU052142,AF098542,AF082034,AF046080,GU052135,AF082035,AF098543,AF098545,AF046088,AF046098,AF082036,DQ767725,AF144305,AF148678,CY014497,CY019408,CY019416,CY019424,CY019432,AM183671,DQ366330,AY609312,HM172114,DQ083578,EF541409,CY094807,AB450561,AB450564,AB450552,EF593102,CY028716,EU268219,EF541408,DQ083565,CY036711,CY034206,CY036695,CY036703,DQ099760,CY009372,EU045362,CY077934,AM920742,CY020501,CY088483,CY112933,GQ293081,CY039087,EU199366,CY035022,CY114381,CY033646,CY034116,EU103823,EU620763,CY002784,CY114373,AB434368,EU296617,AB434376,CY003720,CY003736,CY112396,CY121293,CY113365,CY121309,U26830,CY113677,CY033606,U97740,AF251403,CY095675,AF251427,AF251395,CY112909,CY000801,CY121373,AY531035,DQ487341,GU086113,GU086105,AY852277,FJ970919,EU116040,EU273775,EU273776,EU273777,GU086097,GU086089,CY039079,CY112885,AB019356,AB019355,M73775,M73776,AY619977,AY619969,EU273774,GU086129,GU086137,GU086153,GU086145,CY009300,GU086121,AB537452,AB669134,CY006026,AB275283,CY002096,DQ874876,AF348178,AF348177,AF348179,AF348176,M73772,M73771,AY633252,EU743418,EU743426,EU743433,M21647,CY014671,AB292666,GQ176136,CY006009,L43916,GU051854,JX899805,AB434416,AB434288,AB434296,EU798778,EU798779,DQ280203,DQ139320,EF556203,FJ374511,AGF69142,HM568016,CY057047,CY061757,CY061749,GQ117056,GQ221794,GQ280797,FJ966082,GQ117044,JF915184,FJ969540,CY121680,KC781785,FJ981613,FJ966974,GQ377052,FJ971076,KC780060,GQ117100,CY057044,CY057062,CY057072,HM568080,HM568032,CY057043,CY057066,GQ377072,GQ221812,GQ117119,GQ377082,FJ966952,KC781723,FJ966960,GQ221801,GQ221798,GQ162170,GQ223112,GQ149654,GQ149668,GQ149689,GQ149662,GQ160594,GQ117079,GQ221788,GQ149641,GQ303340,FJ984385,GQ117091,GQ117051,GQ168671,GQ117032,GQ162190,GQ149677,GQ149671,GQ149674,GQ162202,GQ149647,GQ162185,GQ149623,GQ149684,GQ149630,GQ149634,FJ998208,FJ981615,FJ981612,FJ966982,GQ168861,FJ966959,GQ168633,FJ984401,FJ984397,GQ168661,GQ457487,GQ200237,CY057042,CY057051,HM567960,HM568096,CY057046,AF222033,AF222030,AF222028,AF222031,AF222034,AF222029,CY022477,CY022429,CY039917,M81707,CY039925,GU052267,EU798782,EU798787,EU798786,EU798785,EU798781,EU798784,EU798780,EU798783,GU086081,FJ986620,FJ986621,AF455675,EF556199,EF556201,AF389118,CY033577,EF467821,CY009444,AY289928,EU124177,CY058487,KF009550,JN899402,CY030230,CY030232,AF085416,AF085417,AF085414,AF085415,AF085413,L11132,GU186622,CY117235,L11137,CY116835,L11135,CY101555,CY116891,AF290442,AF290440,AF290441,AF290439,CY014710,L11129,L11134,CY032253,L11133,CY125846,CY031595,CY036815,CY032237,L11126,L11125,CY033473,AB558466,J04325,CY015073,AB558465,AF194991,AF194990,EF597263,CY015115,CY022637,CY024746,KC815859,KC815875,KC815867,KC815853,CY021397,CY034720,DQ997325,EU717855,EU717857,HM172455,EU717851,EU373737,EU373734

**Accession number of M1 segment:**

CY015116,GU052217,CY130079,GQ404592,CY024747,JN244129,JN244132,JN244133,JN244127,EU743190,CY005305,CY101556,GU052253,CY014844,CY117236,CY101483,EU743427,EU743434,JN244142,GU053164,CY005828,CY130047,CY087777,JX569043,CY130055,CY087825,CY014664,JN244135,CY014628,CY005553,CY129999,Z26860,AF285886,AY619976,AY619967,CY005907,M63527,GU053111,HQ244410,CY067673,CY067681,CY024819,AY611525,AY646079,GU052939,CY022678,EF015552,AY340091,AB438944,GQ404575,HQ244418,AY648288,AY340089,AY340090,DQ021735,CY127254,CY014787,AB275286,JQ906588,AY633253,JF789605,KC815854,KC815870,CY095578,KC815862,CY021398,KC815878,GU086156,GU086124,CY022342,CY022398,GU086132,GU086140,GU086148,EU502886,GU086036,GU086012,GU086044,GU086052,AB434411,AF251406,CY095678,CY044366,CY130119,CY039992,CY022478,CY024934,CY022430,CY039918,DQ139322,CY061744,GU052268,CY039926,AB434403,CY112886,CY039080,AF400770,GU086100,EU273779,EU273780,EU273781,GU086092,AF400770,CY000802,CY002785,CY114374,CY031796,GU086108,GU086116,M55475,M55474,AY664685,AB256749,AF222822,AF156459,AY664686,AY664690,AF255364,GU052136,AJ278648,AF084282,AF255365,AY664693,AF250482,AF046090,AF036358,AF115286,AF255366,AF046082,AJ278647,AJ278646,AF255363,CY009445,CY033647,CY033578,EF467824,AF389121,DQ874879,FJ200421,FJ200429,CY110944,CY110952,CY014501,CY019403,CY019411,CY019419,CY019427,CY019435,CY014514,CY016293,CY020662,CY021390,CY016301,EU277834,CY016277,DQ529297,CY020678,CY020670,CY020694,CY020702,CY020710,EU148405,EU148373,CY016940,CY016948,CY016924,CY016932,CY016908,EU148357,EU148437,EU148445,EU148381,EU148389,EU148397,CY020646,EF670484,HM172130,CY016285,CY016900,CY017028,CY017180,CY020654,AY609315,AY737298,AB450626,DQ083675,DQ083661,AB450627,AB450629,DQ083688,AB450630,EF593105,EF541449,DQ094263,DQ094259,CY028701,CY029938,CY034199,CY028709,CY028717,CY033170,CY034207,CY036696,CY036704,CY036712,CY094808,EU268222,EF541448,CY014225,CY014421,CY014180,HM172123,CY034723,HM172459,GQ122447,HM172124,AY737292,DQ366333,AY950237,AY950239,AY950240,AY950241,AY684709,AY770077,DQ094265,CY029485,GQ122453,GQ122441,GQ122458,CY014299,EU743087,FJ610095,AB434419,GU086084,L37795,CY015074,GU086028,EU798798,EU798799,EU798804,EU798800,EU798801,EU798802,EU798803,EU798807,EU798805,EU798806,EU399752,EU850625,EU015989,FJ374518,EU850622,M63537,CY014649,GU052382,CY101127,GU052204,CY014680,CY087753,CY130031,CY014651,M63526,GU186778,CY116892,CY015054,AY653194,CY015104,GU051966,CY025254,CY022987,M63534,DQ280204,GU052015,CY089638,CY130087,CY014695,DQ870897,CY077642,L25831,AF250485,EU182256,AF474049,AF144306,M12699,CY022654,GU086020,AF251430,CY005839,GU052676,AJ416630,AY950238,DQ997226,CY116836,EU743419,GU051855,CY014711,GU052691,CY014672,GQ176135,GU052191,CY005625,CY025126,CY025118,CY022638,DQ067438,AY619959,EF597284,M33045,JQ356892,EPI440696,KC885959,KC853764,AGI60298,AGJ51957,AGJ72864,EPI439493,EPI439497,EPI440688,EPI439506,KC464601,JN653660,JN869533,JQ356891,EPI440680,GU086060,GU086068,GU086076,AB434379,AB434339,CY077937,AB434347,AB434363,AB434291,AB434299,EU478802,AB434371,AB434307,CY010581,JX860685,AM920745,AB434331,AB434323,CY009373,AB434315,FJ415612,EF101750,CY116383,JX963610,JX963596,JX860692,JX860700,CY020502,JN375222,JN375223,JN375229,JN375230,JN375231,JN375224,JN375225,JN375226,JN375228,JN375227,HM568019,CY057951,JQ070795,JN638729,JN655558,JQ070780,JQ070787,CY041614,JN655534,HM567979,HM568083,HM568099,HM567963,HM567987,HM567971,HM568043,HM568003,HM568035,JN375210,JN375212,JN375214,JN375217,JN375218,JN375219,JN375220,JN375221,JN375211,GQ117078,GQ457502,GQ200236,GQ457495,GQ168649,FJ984348,GQ232061,GQ457504,GQ168863,GQ117085,GQ168866,GQ457475,GQ168859,FJ984398,FJ984395,GQ221796,GQ117055,FJ984381,GQ117090,GQ117050,GQ117031,JF915185,FJ969513,FJ966954,KC781720,FJ966962,CY121681,KC781788,FJ969527,FJ966975,FJ969537,FJ969532,FJ969518,GQ463205,GQ149657,GQ149690,GQ162179,GQ379812,GQ379817,GQ463208,GQ162192,GQ162188,GQ379824,GQ149624,GQ149642,GQ162178,GQ149679,GQ149629,GQ162175,GQ149638,GQ303341,FJ998211,JN617977,CY061808,CY061736,CY061776,CY061792,CY061800,CY061728,CY061752,CY061760,FJ981617,GQ221800,GQ377073,GQ457485,FJ981608,JN375213,JN375215,JQ290167,JQ290183,CY031341,EU188798,CY064977,CY031391,CY058490,AF255370,M63531,CY032238,JN596876,X59240,CY009301,AF251391,EU273778,AF348191,CY002097,AF348190,AF348194,CY033474,CY031596,CY125847,CY036816,CY032254,AF348189,AF348188,AF348192,AF348193,AF348197,AF348195,AF348196,CY113678,CY033607,CY003721,CY003737,U65577,CY113366,CY121310,CY112397,CY121294,AF401293,FJ970920,AF038273,AF038274,AF251398,CY112934,CY088486,DQ849010,EU620769,CY114382,CY031800,DQ849021,EU097899,CY034117,EU100611,CY039088,CY031812,CY035023,EU116043,CY112910,CY121374,EU097882

**Accession number of M2 segment:**

CY009445,AY768953,AY768952,CY033647,CY033578,EF467824,DQ874879,AY768951,AF389121,CY010581,JQ070795,JN638729,GQ463205,GQ149657,GQ149690,GQ162179,GQ379812,GQ162178,JQ290167,JQ290183,JN375222,GQ457475,GQ117090,GQ162188,CY061752,CY061760,JQ070780,JQ070787,CY041614,JN655534,CY057951,HM567979,HM568083,HM568099,HM567963,HM567987,HM568019,HM567971,HM568043,HM568003,HM568035,JN375210,JN375212,JN375223,JN375229,JN375230,JN375231,JN375224,JN375225,JN375226,JN375228,JN375227,JN375214,JN375217,JN375218,JN375219,JN375220,JN375221,JN375211,GQ117078,GQ457502,GQ200236,GQ457495,GQ168649,GQ232061,FJ984348,GQ457504,GQ117085,GQ168863,GQ168866,GQ168859,FJ984395,FJ984398,GQ117055,GQ221796,FJ984381,GQ117050,GQ117031,JF915185,FJ969513,FJ966954,KC781720,FJ966962,CY121681,KC781788,FJ969527,FJ966975,FJ969537,FJ969532,FJ969518,GQ379817,GQ463208,GQ162192,GQ379824,GQ149624,GQ149642,GQ149679,GQ149629,GQ162175,GQ149638,GQ303341,FJ998211,JN617977,CY061808,CY061736,CY061776,CY061792,CY061800,CY061728,FJ981617,GQ221800,GQ377073,GQ457485,FJ981608,JN375213,JN375215,CY025254,CY022987,AB434371,AB434339,CY077937,AB434307,AB434331,AB434323,AB434315,AB434299,AB434291,AB434347,AB434379,CY020502,JX860692,JX963610,JX860685,JX963596,JX860700,AB434363,GU086060,GU086068,GU086076,FJ415612,EU478802,CY116383,DQ280204,CY089638,GU052015,CY130087,CY014695,EU015989,EU399752,CY095678,FJ374518,GU086084,EU850622,EU850625,CY022342,CY022398,GU086156,GU086124,GU086132,GU086140,GU086148,GU086044,EU502886,GU086052,GU086020,GU086028,GU086012,GU086036,AB434419,CY044366,CY039992,CY130119,CY024934,AB434403,AB434411,CY061744,DQ139322,CY022478,CY039918,CY022430,GU052268,CY039926,JN869533,KC464601,JQ356891,JQ356892,KC885959,KC853764,AGI60299,AGJ51956,AGJ72865,EPI439497,EPI440680,EPI440696,EPI439506,EPI439493,EPI440688,FJ200421,FJ200429,AB450629,AB450626,DQ083661,DQ094265,EF593105,DQ094263,CY029938,AB450627,AB450630,EF541449,DQ094259,CY028701,CY034199,CY028709,CY028717,CY033170,CY034207,CY036696,CY036704,CY036712,CY094808,EU268222,EF541448,M63534,AF156459,EU743087,AJ278648,CY019411,CY019419,CY019427,CY019435,CY019403,CY014514,CY014501,AJ278646,AF255363,AF250482,AJ278647,AF255364,AF255366,AF084282,AF046090,AF036358,AF115286,AF255365,AF046082,GU052136,M55474,M55475,CY116892,FJ610095,GU052676,CY005839,CY034723,CY015074,CY005625,GU052691,CY110944,CY110952,CY130031,CY014651,CY087753,M63537,GU052382,CY014649,CY014225,CY014421,CY014180,CY024747,CY014672,GU052191,GQ176135,AB256749,AF144306,GU052204,CY014680,HM172123,HM172124,AY609315,EU182256,CY014299,AY737298,AY737292,HM172459,GQ122447,AY950237,AY950239,AY950240,AY950241,AY684709,AY770077,GQ122453,GQ122441,GQ122458,JN244142,JN244135,AY653194,HM172130,CY020646,CY016900,CY020654,CY016924,CY016940,CY016948,CY016932,CY016908,EF670484,CY016277,CY016285,CY017028,CY017180,CY020694,CY020702,CY020710,CY016301,CY016293,CY021390,CY020678,CY020670,CY020662,GU186778,M63526,L37795,CY015054,CY005907,M63527,AB275286,CY014628,CY117236,CY067673,CY067681,GU051855,AY619976,AY619967,CY024819,EU743190,CY101483,JN244129,JN244127,AY611525,AY646079,AY648288,JQ906588,AB438944,DQ067438,AY950238,DQ997226,CY130055,CY014664,CY087825,CY101127,CY101556,AF250485,CY022638,CY005828,CY087777,CY130047,AF474049,CY015116,GU052217,CY130079,CY014711,L25831,EU743419,EU743427,EU743434,GU051966,CY015104,JN244132,JN244133,CY005305,GU052253,CY014844,CY077642,GU053164,JX569043,DQ870897,CY005553,CY116836,CY129999,M12699,Z26860,AY619959,GU053111,GU052939,CY022678,CY022654,CY025126,CY025118,CY127254,CY014787,KC815862,CY021398,KC815878,KC815854,KC815870,GU086108,GU086116,CY114382,EU100611,CY034117,CY039088,CY035023,CY121374,CY112910,DQ487329,CY114374,CY112934,CY088486,CY002785,CY000802,AF038274,GU086092,CY113678,CY033607,AF038273,CY003721,AF255370,CY003737,U65577,CY113366,CY121310,CY112397,CY121294,CY112886,AF401293,GU086100,EU116043,AF348194,AF348195,AF348197,AF348191,CY033474,CY032238,X59240,AF348190,AF348196,AF348192,AF348189,AF348188,AF348193,CY002097,M63531,CY125847,CY036816,CY031596,CY032254,CY031341,CY058490,EU188798

**Accession number of NA segment:**

GQ149681,GQ149640,CY061775,CY061791,CY061799,CY061807,CY067743,CY067742,CY067752,CY067722,CY067723,CY067724,CY067751,JN375174,JN375176,JN375175,JN375178,CY041615,CY057952,HM567978,HM568082,HM568098,HM567986,HM567970,HM568042,HM568002,HM568034,CY067725,CY067726,CY067738,CY067739,CY067740,CY067746,CY067747,CY067748,JN375181,JN375182,JN375183,JN375184,JN375185,GQ200238,GQ168648,GQ457505,FJ984350,GQ117081,GQ117084,FJ984362,GQ168627,GQ168660,FJ984357,GQ117099,GQ323479,GQ323567,GQ162189,GQ149672,GQ149670,GQ379820,GQ149631,JN617976,CY061735,CY061727,CY061751,CY061759,JN375177,JN375179,CY010582,CY025255,CY022988,AJ412690,AB434290,AJ410880,EF101756,AB434306,AB434314,EU296604,EU296606,CY116382,GU086067,GU086075,GU086059,FJ415611,JN375186,JN375187,JN375188,JN375189,JN375190,JN375191,JN375192,JN375193,JN375195,JN375194,JX963606,JX860687,JX860695,JX860679,JX963612,AB434298,AF250366,CY005686,CY034722,HM172180,HM172187,DQ094281,CY033171,EF541475,CY036697,CY034208,CY036705,EU118141,CY036713,DQ094287,GU086019,GU086027,DQ280202,CY022343,CY022399,AB434402,CY024935,D31946,M27970,AF250363,CY044367,CY130120,CY039993,DQ280259,CY022479,CY039919,CY022431,GU052269,CY039927,AJ518104,KF009551,CY058489,EU124136,AF250357,CY033579,CY009446,EF467823,AF389120,EU743420,EU743428,EU743435,GU186779,CY014993,EU429796,GQ176134,CY014673,AB292667,AB472063,EF015553,AY340079,AY340077,AB438943,AY340078,JN244223,CY014788,CY127255,CY067672,CY067680,CY024820,EPI440684,EPI440692,AGJ72863,KC885958,AGJ51955,EPI439487,KC853765,AGI60300,EPI440700,EPI439509,EPI439500,JF789604,HQ244409,GU060484,HQ244417,AF285887,CY110943,CY110951,EU429795,AB288846,CY014681,AY207549,CY101128,AB295612,GU052383,CY101484,CY089639,CY130088,CY014696,AY207553,AB292665,GQ921324,AY611526,AY646080,AY648289,AY619975,AY619966,DQ870890,AB276110,DQ870896,CY101557,CY117237,JQ906584,CY024748,AY664704,KC464600,JQ356876,JQ356877,JN653644,JN869532,AJ404629,AJ404628,AY664712,AY664709,AY664705,AB256748,AB472016,AB434346,EU296614,AB434362,EU296608,AB434338,GU052218,CY130080,AB288335,EU429794,M24740,AB278601,FJ200416,FJ200424,L06574,AB275285,CY005402,L06575,AY633254,L06585,GU186623,CY130048,EU429793,CY087778,CY014660,AB289344,AY207528,X52226,AB434330,AB434322,EU296602,EU296600,AF250481,AF102656,AF084272,GU052137,AF084271,AF036357,AF102663,AF028708,GU052144,AF046081,AF046089,AGF69137,AGF69140,AGF69139,KC781784,GQ377078,GQ117028,GQ221789,GQ117077,GQ160593,GQ221795,GQ117053,FJ984383,GQ323542,GQ168632,GQ117048,GQ168670,JF915186,FJ966084,FJ969517,FJ984386,CY121682,HM138502,GQ377050,GQ377079,GQ149659,GQ223113,GQ149650,GQ162169,GQ149688,GQ149656,GQ149644,GQ149622,GQ149664,GQ162187,GQ162201,FJ998214,FJ981614,GQ377071,GQ221799,FJ966981,GQ323512,GQ457486,GQ221813,GQ117118,GQ117105,GQ221802,FJ966956,KC781721,FJ971075,HM567962,CY067717,CY067718,CY067719,CY067720,CY067721,CY067729,CY067731,CY067732,CY067734,CY067735,CY067736,CY067737,CY067741,CY067749,CY067750,CY067715,CY067716,CY067728,CY067730,CY067733,CY067744,CY067745,HM568018,CY067727,GU086091,FJ970921,AJ457966,CY121375,CY112911,AY531036,CY000803,EU273785,CY001501,EF556204,FJ374514,EF556200,EU798822,EU798827,EU798825,EU798826,AF251396,U71143,AF038263,AJ457945,CY112887,CY039081,AJ291403,AF038264,EU798824,EU798820,EU798823,EU798821,AF251404,AF251428,AF455691,CY095677,CY028702,CY029939,CY034200,CY028710,CY028718,CY094809,AB450610,AB450607,AB450596,EU268221,EF541470,AB450601,AB450598,AY649383,EF593104,AB450602,AB450604,AB450606,AB450605,AB450608,AM183680,DQ366332,CY014298,GQ122451,GQ122439,GQ122457,GQ122445,CY019402,CY014515,CY014502,CY019410,CY019418,CY019426,CY019434,CY014422,AF144304,AY737299,HM172173,EU148398,EU148406,CY016901,EF619973,EU717856,CY020647,CY020655,CY016909,CY016925,CY016949,CY016941,CY016933,CY016286,CY017029,CY017181,CY020695,CY020703,CY020711,CY016278,DQ529296,CY020679,EU277835,CY016302,CY016294,CY020663,CY021391,CY020671,AY653195,AY609314,HM172463,EF670483,AY737291,CY029484,AY950245,DQ997220,AY684708,AY770078,AY950244,AY950246,AY950247,AY950248,AY619960,AF250362,AB271114,KC815851,CY021399,KC815869,KC815861,CY095579,KC815880,KC815884,KC815885,KC815877,KC815882,KC815881,KC815883,AB271116,AB470661,CY022679,CY025119,AJ416627,CY022655,CY025127,AB470663,AB470669,EU139833,EU798818,EU798819,GU086035,GU086051,GU086011,GU086043,EU502888,AB434418,CY005626,CY116893,D00713,AB434410,CY032239,CY125848,CY036817,CY031597,CY033475,AB124657,AF348187,AF348186,AF348185,AF348184,CY005554,CY032255,AY209895,CY022639,CY130056,AB295602,CY087826,CY014665,AJ574907,CY015117,EU182255,AF474039,CY015075,CY130032,CY087754,AB296073,CY005908,CY116837,CY130000,EU743021,CY005306,GU052692,FJ610096,CY005840,GU052677,CY009374,AM920743,CY020503,JQ290187,JQ290171,JQ070767,JQ070775,JN866185,JN655533,JQ070799,JN638732,JN655557,JQ070791,JN992752,JQ070783,GU937748,EU399753,AJ412697,AB434370,AB434378,EU296618,AJ293934,GU086139,EU273782,CY009302,GU086123,GU086131,GU086155,AF251389,AB124659,CY002098,DQ139321,CY061743,U42770,U43427,CY113679,CY033608,U42635,CY003722,CY003738,CY121311,CY121295,CY113367,U42633,CY112398,GU086083,EF556202,U51247,CY081429,GQ293082,CY039089,CY116578,CY035024,EU199420,CY112935,CY088485,CY002786,CY033648,CY114383,EF541463,EU103981,EU620764,CY034118,EU146841,CY114375,EF512561,EF541462,EU273784,DQ487331,EU273783,EU116042

**Accession number of NP segment:**

CY017182,AY653196,AY664724,AY770081,HM172224,HM172467,EF593103,CY014423,DQ099774,AB450584,AB450581,AY609313,AY737297,AY950251,AY950253,AY950254,AY950255,AY684707,AB450580,AB450583,DQ099772,DQ099768,CY028703,CY029940,CY034201,CY028711,CY028719,CY033172,CY034209,CY036698,CY036706,CY029483,EU268220,EF541459,CY019401,CY019409,CY019417,CY019425,CY019433,CY014242,CY014516,CY014503,CY014252,GQ122407,CY014297,CY036714,CY094810,GQ122408,GQ122406,GQ122409,CY014178,M30768,CY015076,CY110950,CY110942,CY015055,GU186780,CY014994,EU743191,CY087755,CY130033,CY014652,CY005841,FJ610097,CY067679,CY024749,M27298,Z26855,CY067671,M63774,GU051968,CY015105,M63778,M63784,M30762,DQ870889,DQ870895,EU743022,GU053166,CY014789,JN244154,CY015118,JN244144,AF144303,M63780,GU052205,CY014682,CY022656,CY025128,CY025120,HQ541732,M63773,DQ067440,EU084948,CY005555,EU743131,CY101129,CY014674,GQ176133,CY014712,M63781,JN244157,JN244158,CY005687,M63783,CY087827,CY130057,CY014666,CY005829,CY033580,CY047401,EF467822,CY009447,AF389119,DQ874877,CY047398,CY058488,AF255749,AF251399,CY047417,EU116041,EU273787,EU273788,FJ970922,EU273786,GU086090,CY121376,DQ487330,CY112912,AB019359,AB019360,CY000804,AF038258,AF038259,CY039082,CY112888,CY114376,CY002787,CY039090,CY035025,CY047397,CY033649,CY034119,CY088484,CY112936,EU620768,CY114384,JN596875,CY032256,AY210068,M22577,L07367,CY113368,CY112399,CY121296,CY121312,U71147,CY003723,CY113680,CY003739,CY033609,AF348180,AF348183,AF348181,CY125849,CY031598,CY036818,CY032240,CY033476,CY002099,AF072545,GU086154,GU086138,EU273789,CY009303,GU086122,GU086146,AF251392,GU086130,FJ200420,FJ200428,AF251407,AB434377,AB434361,AB434345,JN375138,JN375140,JN375139,FJ969536,GQ303342,JN375150,JN375151,JN375152,JN375153,JN375154,JN375155,JN375156,JN375157,JN375158,JN375159,CY061806,GQ149648,GQ117098,FJ984396,JN375149,HM568001,HM567985,GQ200235,GQ377056,GQ149639,JN617975,CY041616,CY057953,HM567977,HM568081,HM568097,HM567961,HM568017,AF222777,AF222778,AF222774,AF222773,AF222770,AF222772,AF222771,AF222775,L46850,CY022432,CY039920,L46849,CY039928,AF222768,AF222769,CY022480,GU086042,GU086018,GU086026,GU086010,GU086034,GU086050,HM172237,AY664731,CY089640,CY014697,CY130089,M27521,GU052016,AF156403,AY664728,AB256747,GQ921323,CY005403,AF028710,AJ289873,AJ289871,AF255742,AF250480,AJ289872,AF255743,AF255744,AF115284,AF036359,AF098620,AF255745,AF098623,AF046092,AJ291400,AF084276,AF098621,GU052145,AF046084,AF057293,AF098618,AF098619,AF098617,M30747,M21937,M24660,M24556,M22576,M24557,EPI439491,JN869531,KC464599,KC885957,KF001510,AGJ51954,AGJ72862,EPI439496,EPI440678,EPI440686,EPI439505,JQ356879,EPI440694,JQ356880,DQ366331,CY034721,AY664723,AY737290,HM172228,CY020648,CY016902,CY020656,EU277836,CY016303,CY016295,CY020664,CY021392,CY020680,CY020672,CY016934,CY016279,CY020696,CY020704,CY020712,EU148367,EU148391,EU148447,EU148439,CY016910,CY016287,CY016942,CY016950,CY016926,EU148359,EU148399,CY017030,CY087779,CY130049,GU052219,CY130081,M63776,M30769,CY005909,CY127256,CY022680,D00050,M14921,CY116838,CY130001,JF789603,GU060483,HQ244416,M30760,CY014650,GU052384,CY022640,CY005627,JQ906580,CY095580,KC815852,KC815860,KC815868,CY021400,KC815876,EU743436,EU743421,EU743429,AY611527,AY646081,AY648290,CY024821,CY101558,CY005307,CY014845,CY117238,CY101485,GU051856,AF285888,AY619974,AY619968,AY619958,AY342427,AB438942,AY342425,AY950252,DQ997221,AF079571,AB275284,HQ244408,CY116894,GU052255,DQ997327,AB434337,GU086058,CY010583,FJ415617,GU086074,GU086066,CY025256,CY022989,AM920744,AF385293,CY077935,JX963595,JX860683,JX963614,JX860691,JX860699,AB434329,AB434321,AB434305,AB434313,CY020504,CY009375,CY116381,AB434297,AB434289,AB434369,HM567969,HM568041,HM568033,JN375142,JN375145,JN375146,JN375147,JN375148,FJ984352,GQ117080,GQ117083,FJ984363,FJ984358,GQ149673,GQ149678,GQ149682,GQ149628,GQ149637,CY061734,CY061774,CY061790,CY061798,CY061726,CY061758,JN375141,JN375143,GQ232062,GQ149667,CY121683,GQ117104,GQ160592,GQ117074,GQ117087,FJ966953,KC781722,FJ966961,GQ149660,GQ149655,GQ149685,GQ162184,GQ149645,GQ149619,GQ117117,GQ117052,KC780063,FJ984366,GQ457484,FJ984384,GQ117045,GQ117025,JF915187,FJ966083,FJ969512,GQ338390,GQ338345,FJ998217,FJ981618,FJ966979,FJ966967,FJ981609,EU798842,EU798847,EU798845,EU798846,EU798844,EU798841,EU798840,EU798843,EU399754,AF251431,GU086082,EU015990,EU850621,EU850624,FJ374515,CY095676,GU937747,JQ070761,JQ290165,JQ290181,JQ070793,JN638727,JN655552,JN992748,JQ070785,JQ070778,JN866179,JN655536,EU798838,AF455699,EU798839,M76608,AB434409,AB434417,DQ139324,DQ280201,EU502887,CY061742,AB434401,M63754,M76606,CY044368,CY130121,CY039994,CY022344,CY022400,M63765,CY024936,AF222776

**Accession number of NS1 segment:**

M16623,GU052694,AF144307,CY005842,GU052678,AF474059,EU743023,FJ610098,GQ176132,CY014675,CY014846,J02105,GU052220,CY130082,U96740,AY619957,CY005308,AY633256,CY005556,CY022657,CY025129,CY025121,AB275287,GU052942,CY022681,U96737,CY089641,CY014698,M80959,CY130090,AB434340,AB434308,AB434316,EF101751,AB434420,DQ280200,AB434412,CY044369,CY039995,CY130122,AB434404,CY022345,CY022401,CY024937,AB434380,CY061753,CY061761,GQ377090,GQ117106,GQ117030,GQ457489,GQ117054,FJ984382,GQ168869,GQ168853,JF915191,FJ969514,FJ966086,KC781719,FJ971074,KC781782,CY121684,FJ969528,FJ969538,FJ969533,FJ969519,GQ149658,GQ149687,GQ379814,GQ379818,GQ149621,GQ149643,GQ379816,GQ149626,GQ149680,GQ149635,FJ998220,FJ981620,FJ966966,FJ981611,JN375258,JN375259,JN375260,JN375261,JN375262,JN375263,JN375264,JN375265,JN375266,JN375267,JN375253,JN375254,JN375255,JN375256,JN375257,CY061737,CY061729,CY041617,CY057954,HM567980,HM568084,HM568100,HM567964,HM567988,HM568020,HM567972,HM568044,HM568004,HM568036,JN375250,GQ200239,FJ984349,GQ232063,GQ168862,GQ168868,FJ984361,FJ984356,GQ168872,GQ377098,FJ984399,JN617978,CY061809,CY061777,CY061793,CY061801,JN375249,JN375251,JN375247,JN375248,JN375246,AB434348,GU086045,GU086029,EU502889,GU086021,GU086013,GU086037,GU086053,EU116044,DQ139323,CY061745,AB434372,EU399755,EU850623,EU850626,GU086085,EU015988,FJ374516,EU798864,EU798860,EU798861,EU798863,EU798862,EU798867,EU798865,EU798866,EU798858,EU798859,AF455707,JQ070794,JN638728,JN655553,JQ070786,JQ070779,JN992753,GU937750,JN866180,JQ290166,JQ070762,JQ290182,JQ070770,JN655538,CY022481,AY619979,CY095679,AF251432,AF251408,CY039921,CY022433,AY619978,DQ280255,CY039929,GU052271,AB434332,AB434324,AB434300,GU086101,GU086077,GU086061,GU086069,CY116384,AJ344033,JX963611,JX860693,JX963597,JX860684,JX860701,AB434364,AB434292,CY025257,M80963,CY022990,AJ344029,CY010584,CY009376,AJ344034,CY020505,AM920746,CY077938,AJ344024,AJ278649,AF256176,AF250483,AF256177,AJ404735,AF256178,AF115288,AF098574,AF256179,AJ404736,AF046091,AF036360,AF084285,AF098573,CY009448,EF467817,CY003724,CY003740,CY112937,CY088487,AM502797,AF256183,CY113369,CY121313,CY112400,CY121297,AF038278,CY000805,CY034120,CY039091,CY035026,CY114377,EU620770,CY121377,CY112913,EU097912,DQ487332,CY002788,CY033650,CY114385,EU097932,AF038279,CY113681,CY033610,AF251400,D30667,EU273792,AF400773,AF400773,CY112889,CY039083,EU273791,EU273793,GU086093,FJ970923,JN596877,CY032257,AY210151,EU273790,GU086157,CY009304,GU086125,GU086133,GU086149,GU086141,CY002100,AF348198,AF348201,AF348205,AF348206,EU403423,EU403422,EU403421,D10571,M34829,AF348204,AF348200,AF348199,AF348202,AF348203,CY033477,CY125850,CY031599,CY036819,CY032241,AF098576,AF098570,AF098571,GU052138,AF098572,GU052146,AF098569,AF046083,KC464602,JN653676,AGJ72866,KF001511,KC885960,AGJ51959,EPI439494,EPI439499,EPI440679,EPI440687,EPI440695,EPI439510,AY664743,AY664750,AY664747,AB256750,AF222824,JQ356894,JQ356895,AY664742,JN869534,GU086117,GU086109,M80965,M33046,FJ200422,FJ200430,M60799,V01101,CY005404,CY015077,CY110945,CY110953,AJ410589,AJ410593,L37798,CY015056,GU186781,CY014995,U96745,GU052206,CY014683,CY116895,M25375,JN244261,JN244259,AF250502,EF583621,AY611528,AY646082,AY648291,CY130034,CY014653,CY087756,L25830,CY077646,U85376,CY015106,GU051969,CY024822,DQ021611,DQ067441,CY087828,CY014667,CY130058,CY006039,GU052385,JQ906592,CY117239,CY101486,CY101130,AF285889,AY950259,DQ997222,EF597375,CY015119,CY130050,CY014661,CY087780,EF015555,AY342422,AY342424,AB438945,CY014629,EU743422,EU743437,GU051857,JN244258,JN244257,CY024750,CY005628,Z26864,KC815863,KC815879,GU052256,CY130002,CY127257,CY067682,AY619973,AY619965,CY014713,CY101559,JN244268,CY022641,GU053167,JN244265,M25376,CY116839,M80945,CY005910,GU053113,HQ244411,CY067674,GU060485,HQ244419,CY014790,JF789606,KC815855,CY021401,KC815871,CY095581,HM172471,CY016927,CY017031,CY016943,CY016951,CY016911,CY016288,CY017183,CY016903,EU148360,EU148376,EU148384,EU148400,CY020649,CY020657,EF619974,CY020697,CY020705,CY020713,CY020673,CY016935,EU148408,EU277837,EU148440,EU148448,CY016280,DQ529294,CY016304,CY016296,CY020665,CY021393,CY020681,EU148368,EU148392,GQ122452,GQ122446,CY014300,GQ122440,GQ122459,CY014268,CY014517,CY014256,CY014181,CY014424,CY014504,CY019404,CY019412,CY019420,CY019428,CY019436,AY653197,AY609316,CY029486,AY737300,HM172287,HM172286,HM172303,AY737285,DQ366334,CY034724,AY950258,AY950260,AY950261,AY950262,AY770080,AY684710,CY036715,CY094811,EF593106,AB450645,EU268223,EF541458,DQ083650,DQ083623,AB450642,AB450643,DQ083637,AB450646,CY034210,CY036707,CY036699,AY770615,CY033173,AY770619,CY028704,CY029941,CY034202,CY028720,AY770621,CY028712,CY064978,CY058491,DQ874880,CY033581,AF389122

**Accession number of NS2 segment:**

EU743023,AF474059,AF144307,CY005556,AB275287,CY005842,GU052678,GU052694,FJ610098,GQ176132,CY014675,GU052942,CY022681,CY022657,CY025129,CY025121,CY014846,AY619957,J02105,CY130082,GU052220,CY005308,U96740,DQ870898,JQ356894,DQ280200,AB434372,AB434380,GU086085,EU850623,EU015988,EU850626,FJ374516,CY061745,AB434340,CY061777,CY061793,CY061801,JN375248,JN375246,JN375251,CY061737,CY061729,HM568044,GQ377090,GQ117106,KC781719,FJ971074,CY041617,CY057954,HM567980,HM568084,HM568100,HM567964,HM567988,HM568020,HM567972,HM568004,HM568036,JN375247,JN375258,JN375259,JN375260,JN375261,JN375262,JN375263,JN375264,JN375265,JN375266,JN375267,JN375250,JN375253,JN375254,JN375255,JN375256,JN375257,GQ457489,GQ200239,FJ984349,GQ232063,GQ168862,GQ168868,FJ984361,FJ984356,FJ984399,GQ377098,GQ168872,GQ117054,FJ984382,GQ168869,GQ168853,GQ117030,JF915191,FJ966086,FJ969514,KC781782,CY121684,FJ969538,FJ969528,FJ969519,FJ969533,GQ149687,GQ149658,GQ379814,GQ379818,GQ149643,GQ379816,GQ149621,GQ149626,GQ149680,GQ149635,FJ998220,JN617978,CY061809,CY061753,CY061761,FJ981620,FJ966966,FJ981611,JN375249,GU086045,EU116044,EU502889,GU086021,GU086013,GU086037,GU086053,GU086029,AB434348,GU052271,CY039929,AY619979,CY022481,CY039921,CY022433,EU798861,EU798864,EU798860,EU798863,EU399755,AF455707,EU798862,EU798867,EU798865,EU798866,AY619978,DQ280255,AF251432,EU798858,EU798859,AF251408,CY095679,JN866180,JQ070794,JN638728,JN655553,JQ070762,JQ290166,JQ290182,JQ070770,GU937750,JN655538,JQ070786,JN992753,JQ070779,AB434420,AB434412,AB434308,AB434316,AB434404,CY044369,CY039995,CY130122,CY022345,CY024937,CY022401,JQ356895,CY058491,CY089641,U96737,CY014698,CY130090,CY033581,AF389122,EF467817,CY009448,DQ874880,GU086101,AB434332,AB434324,CY016911,CY016943,CY016951,CY016927,CY016288,CY017031,CY017183,EU148448,CY016935,CY020649,CY016280,CY016304,CY016296,CY020665,CY021393,CY020681,CY020673,CY016903,CY020657,CY020697,CY020705,CY020713,EU148384,AY609316,HM172471,AY684710,AY770080,HM172303,CY094811,CY029486,CY036715,AY770621,AY770615,CY028704,CY029941,CY034202,CY028712,CY028720,CY033173,CY034210,CY036699,CY036707,AB450642,AY737300,EF593106,AB450643,AB450645,AB450646,EU268223,EF541458,CY014300,GQ122452,GQ122446,GQ122440,GQ122459,CY019404,CY019412,CY019420,CY019428,CY019436,CY014517,CY014504,CY014268,CY014256,CY014424,CY014181,CY034724,AY950260,AY950261,AY950262,HM172287,HM172286,AY737285,FJ200422,FJ200430,CY116384,AJ344033,GU086061,GU086069,GU086077,CY020505,CY010584,CY025257,CY022990,CY077938,CY009376,AJ344029,AB434364,AB434292,AJ344034,AB434300,AY664743,AY664750,JN596877,CY032257,AY210151,CY015077,GU052146,AF046083,AF098569,AF098571,GU052138,AF098572,AF098570,CY033477,GU086157,CY003724,CY003740,CY002100,CY009304,GU086125,GU086133,GU086141,GU086149,CY088487,CY112937,AM502797,AF256183,FJ970923,CY113681,CY033610,CY114377,AF038278,CY121377,CY112913,DQ487332,CY000805,CY002788,CY033650,CY113369,CY121313,CY112400,CY121297,CY039083,CY112889,CY114385,CY034120,AF038279,CY039091,CY035026,GU086093,AF251400,M34829,AF348206,CY032241,CY125850,CY031599,CY036819,D10571,AF348204,AF348205,AF348198,AF348203,AF348202,AF348199,AF348200,AF348201,AY664747,AB256750,CY110945,CY110953,AY653197,AF250502,JN244265,AY664742,CY117239,U96745,GU086109,GU086117,JN869534,KC885960,KF001511,AGJ51958,KC464602,JN653676,AGJ72867,L37798,AY950259,DQ997222,M25375,CY024750,CY116895,GU052206,CY014683,CY015056,GU186781,CY014995,EF597375,CY015119,CY101486,V01101,Z26864,JN244257,JN244258,CY101559,GU052256,GU051857,EU743422,EU743437,JN244268,CY022641,GU053167,M60799,M25376,CY116839,CY130002,AF285889,AY619973,AY619965,CY005910,GU053113,HQ244411,CY067674,CY067682,AB438945,GU060485,HQ244419,CY014790,JQ906592,KC815855,KC815879,KC815863,CY095581,CY021401,KC815871,EF583621,CY130058,CY014667,CY087828,DQ067441,AY611528,AY646082,AY648291,CY127257,AY950258,CY024822,CY101130,JF789606,CY014713,CY005628,U85376,CY015106,GU051969,CY130050,CY005404,CY006039,GU052385,JN244259,L25830,CY077646,CY087780,CY014661,CY014629,AF098574,AJ404736,AF036360,AF046091,AF256178,AF256179,AF098573,AF098576,AF084285,AJ278649,AF256176,AF250483,AJ404735,AF256177,AF115288,CY014653,CY130034,CY087756

**Accession number of PA segment:**

JN992754,JQ070788,JQ070781,JQ070796,JN638730,JQ290168,JQ070764,JQ070772,JQ290184,JN866182,AF251409,AF251433,EU798878,EU798879,CY095674,JQ356883,KC885955,AGJ51952,KC464597,KF001509,AGJ72860,EPI439490,EPI439498,EPI440681,U71139,CY113370,CY121314,CY112401,CY113682,CY033611,CY121298,EU273797,AF037429,EU273795,CY114378,CY002789,CY112938,CY088482,EU273796,AF037428,EU116039,CY112890,CY039084,CY000806,CY121378,CY112914,EU097809,DQ487327,AF348175,AF348174,CY003725,CY003741,CY036820,M26079,CY031600,CY125851,CY033478,CY002101,CY032242,M26076,AJ404636,AF257192,AJ404637,AF257191,AF250478,AF257194,AF098609,AF098608,AF098611,AJ289874,AF084267,AF257193,AF115294,AF084268,GU052139,AF098607,AF098606,AF098605,GU052147,AF046087,AF098604,AB256745,GU086080,FJ374517,CY061804,HM567999,HM567975,HM567967,JN375085,JN375083,JN375084,JN375086,JN375087,GQ457490,GQ457472,CY061748,CY061756,JN375066,JN375068,JN375067,FJ998223,EPI440697,EPI439503,EPI440689,JN869530,JQ356882,FJ610099,HM172320,AF144302,X17223,HM172313,HM172475,AY770082,CY019399,CY019407,CY019415,CY019423,CY019431,CY014418,CY014498,CY014519,CY014328,CY014330,AY737295,CY014169,EU148409,HM172319,GQ122438,GQ122456,DQ366329,AY653198,AY737288,CY016904,CY020650,CY020658,EU148449,CY016281,EU148393,EU148369,EU148385,EU148361,EU148377,EU148401,CY020698,CY020706,CY020714,EU277838,CY016912,CY016952,EU148441,CY016936,CY016289,CY017184,CY016928,CY014654,M26083,AB274965,CY022658,CY025122,CY025130,CY130083,JX978285,GU051970,AY950266,DQ997223,AY703831,AY342420,AB438940,EF015556,AY342418,GU052943,CY022682,HQ244406,JN244184,JN244179,JF789601,JN244168,JN244166,AY609311,CY021402,CY095582,KC815858,KC815866,KC815874,KC815850,GU060481,HQ244414,CY024751,CY005557,CY089642,CY130091,CY014699,AJ243994,CY116840,CY130003,CY005688,CY127258,CY101131,CY117240,CY101487,M26084,GU053168,CY014791,CY005911,EU743192,CY014714,JQ906572,EU743438,EU743423,EU743430,CY116896,CY005629,DQ870887,CY101560,HQ541733,GU051858,CY067677,CY024823,FJ969539,JF915188,FJ969515,FJ966081,KC781786,CY121685,FJ966977,FJ969529,KC780062,GQ377053,FJ984368,GQ200264,GQ457496,FJ966957,KC781716,FJ966964,GQ149653,GQ149661,GQ149686,GQ463206,GQ149646,GQ149620,JN375077,JN375072,JN375073,JN375074,JN375075,JN375076,JN375078,JN375079,JN375080,JN375081,HM568015,HM568095,HM568079,CY061732,CY061724,CY061772,CY061788,CY061796,CY041618,CY057955,HM567959,HM567983,HM568039,HM568031,JN375070,FJ984354,GQ200259,GQ168865,FJ984359,FJ984400,GQ117049,GQ117029,GQ149676,GQ379813,GQ149627,GQ149636,CY097780,FJ981619,FJ969524,FJ981610,FJ966970,JN375069,JN375071,AF455715,EU798882,EU798887,EU798885,EU798886,EU798884,EU798881,EU798883,EU798880,EU399756,EU015991,CY061740,FJ200419,FJ200427,AB434415,GU086024,EU502890,GU086008,GU086032,GU086016,GU086048,GU086040,AB434407,DQ139326,AB434399,CY022346,CY022402,CY024938,CY044370,CY130123,CY039996,DQ280258,CY022482,CY022434,CY039922,CY039930,DQ280254,CY010585,AB434295,CY009377,AB434359,AB434327,AB434319,AB434303,AB434311,FJ415616,JX860690,JX963609,JX860682,JX963594,JX860698,CY025258,CY022991,AJ311465,AJ312836,CY116379,AB434367,AB434287,CY020506,CY077933,AB434335,AB434343,AB434375,GU086064,GU086072,GU086056,DQ280199,CY033582,CY009449,EF467820,AF389117,DQ874875,CY058486,JN596874,CY032258,AY209991,AF257198,AF251393,GU086136,CY009305,GU086128,EU273794,AF251401,EU620767,CY035027,CY033651,CY114386,EU097833,CY034121,CY016944,CY017032,CY016305,CY020674,CY016297,CY020666,CY021394,CY020682,CY029482,CY014295,GQ122444,GQ122450,CY034211,AY684705,AB450534,AB450535,AB450538,AY950265,AY950267,AY950268,AY950269,EU268218,AB450537,DQ099788,DQ099790,DQ099787,CY028705,CY029942,CY034203,CY028713,CY028721,CY033174,CY036700,CY036708,CY094812,CY036716,CY110940,CY110948,CY005843,GU052679,CY015078,DQ997328,CY034719,AF285890,DQ067442,GU052207,CY014684,AY619972,AY619964,AY619956,GQ921321,AY648292,AY616764,AY646083,EU743024,GU052695,CY014676,GQ176131,GU052257,CY014996,GU186782,CY015057,EU084947,GU053114,CY022642,CY005309,CY005820,EU743132,CY067669,CY014668,CY130059,CY087829,CY087781,CY005830,CY130051,CY087757,CY130035

**Accession number of PA-X segment:**

AB434415,EU502890,GU086016,GU086008,GU086032,GU086040,GU086048,GU086024,CY022346,CY022402,CY024938,CY044370,CY039996,CY130123,DQ280258,DQ139326,GU052272,CY039930,CY022434,CY039922,DQ280254,CY022482,AB434407,AB434399,CY033582,CY009449,EF467820,DQ874875,AF389117,CY064973,CY058486,CY032258,AY209991,AX399726,CY033478,CY036820,CY125851,CY031600,M26079,AF348175,CY032242,AF348174,CY002789,CY114378,CY088482,CY112938,EU620767,CY033651,CY114386,EU097833,CY034121,CY035027,CY039092,CY009305,GU086128,GU086136,EU273794,AF251393,EU273797,U71139,CY002101,AF037428,AF257198,CY000806,CY112401,CY113682,CY033611,CY003725,CY003741,CY121378,CY112914,EU097809,DQ487327,CY113370,CY121314,CY121298,CY112890,CY039084,AF037429,EU273795,EU273796,AF251401,EU116039,AB434335,AB434367,CY009377,AB434327,AB434319,EF101755,AB434359,AB434303,AB434311,AB434295,AB434343,AB434375,CY010585,GU086072,GU086056,GU086064,AB434287,AJ312836,CY116379,CY020506,AJ311465,AJ311204,CY025258,CY022991,CY077933,JX860682,JX963594,JX860698,FJ415616,JX963609,JX860690,FJ200419,FJ200427,DQ280199,AF222820,AF156445,AY664766,AB256745,FJ374517,CY061740,GU086080,EU015991,JN653596,AY770082,DQ366329,CY016281,DQ529295,HM172475,AY653198,CY094812,CY014498,CY014418,CY014328,CY019399,CY019407,CY019415,CY019423,CY019431,CY014519,CY014330,CY014169,EU148369,EU148393,EU148385,EU148377,EU148401,EU148361,EU148441,EU148449,EU148409,CY014295,AY737295,EF619979,AY737288,CY020650,CY016904,CY020658,CY016928,CY016936,HM172319,CY016305,CY016297,CY020666,CY021394,CY020682,CY020674,CY016912,CY016952,CY016944,EU277838,GQ122444,AY950265,AY950267,AY950268,AY950269,AY684705,CY016289,CY029482,GQ122450,CY017032,CY017184,CY020698,CY020706,CY020714,GQ122438,GQ122456,CY036700,CY036708,CY036716,DQ099788,DQ099790,DQ099787,CY028705,CY029942,CY034203,CY028713,CY028721,CY033174,CY034211,EU268218,EF473503,AB450535,AB450534,AB450537,AB450538,EF593101,GQ457472,JN375066,JN375068,JN375067,CY061748,CY061756,CY061804,JN375085,JN375083,JN375084,JN375086,JN375087,GU053168,CY117240,M26084,CY005911,FJ969539,JN375072,JN375073,JN375074,JN375075,JN375076,JN375077,JN375078,JN375079,JN375080,JN375081,CY130091,GQ200264,GQ457496,FJ966957,KC781716,KC781716,FJ966964,CY041618,CY057955,CY005688,CY095674,EF015556,AY342418,AY342420,JN375070,GQ457490,FJ984354,GQ200259,GQ168865,FJ984359,FJ984400,GQ117049,GQ117029,JF915188,FJ969515,FJ966081,CY121685,CY121685,KC781786,KC781786,FJ969529,FJ966977,KC780062,KC780062,GQ377053,FJ984368,GQ149653,GQ149661,GQ463206,GQ149686,GQ149676,GQ149646,GQ149620,GQ149627,GQ379813,GQ149636,FJ998223,CY097780,CY061732,CY061772,CY061788,CY061796,CY061724,FJ981619,FJ969524,FJ966970,FJ981610,JN375069,JN375071,DQ997328,AF144302,GQ200264,EU798884,EU798880,EU798881,EU798883,EU798882,EU798887,EU798885,EU798886,CY015057,CY014996,GU186782,CY034719,KC464597,JQ356883,KC885955,KF001509,JQ356882,JN869530,AF455715,GU052139,AF098607,GU052147,AF046087,AF098604,AF098606,AF098605,AJ404636,AF257192,AF250478,AJ404637,KC780062,AF257191,AF098608,AF098609,AF098611,AJ289874,AF046095,AF036361,AF257194,AF084267,AF115294,AF257193,AF084268,AY664761,FJ610099,EU399756,AJ243992,X17223,GU052679,CY005843,CY005820,EU743024,CY015078,CY110940,CY110948,GU052695,AY609311,CY014684,GU052207,AF285890,CY022658,CY025122,CY025130,AY664762,CY130083,JX978285,EU084947,CY130051,CY005830,CY087781,CY005309,AY950266,DQ997223,GQ921321,JN244171,JN244170,GU937746,JN866182,JQ070796,JN655555,JN638730,JQ070781,JN992754,JQ070788,JQ070764,JQ290168,JQ070772,JQ290184,JN655535,AF251409,AF251433,EF597410,CY005629,GU052257,CY022642,EU743132,AY619972,AY619964,CY014654,CY087757,CY130035,GU051970,GU052194,CY014676,GQ176131,CY067669,CY130059,CY014668,CY087829,CY127258,JF789601,HQ244406,JN244184,JN244168,JN244166,JN244179,CY095582,KC815858,KC815874,CY021402,KC815866,KC815850,KC815850,KC815858,KC815866,KC815874,AY646083,CY101487,EU743192,AY619956,GU053168,CY117240,M26084,CY005911,AY616764,AY648292,CY014791,DQ870887,DQ870893,CY024823,CY089642,CY101560,CY067677,HQ541733,CY014699,CY130091,AY703831,EU743430,EU743438,EU743423,CY101131,EU798878,EU798879,JQ906572,CY005688,CY095674,EF015556,AY342418,AY342420,AB438940,CY116896,CY014714,CY077647,GU052943,CY022682,CY024751,CY005557,CY116840,AJ243994,CY130003,GU053114,GU060481,HQ244414,M26083,AB274965

**Accession number of PB1 segment:**

CY061747,CY061755,FJ981616,FJ969526,FJ969522,GU086079,FJ374513,EU015992,AF258823,AF251397,AF251390,EU273798,GU086127,GU086135,CY002102,CY009306,CY033652,CY114387,EU097801,CY034122,EU273801,CY039093,CY035028,EU620766,CY002790,CY114379,CY088481,CY112939,CY113683,CY033612,CY112402,CY003726,CY003742,CY113371,CY121315,CY121299,AF037423,AF037422,U71131,CY039085,CY112891,CY000807,EU273799,EU273800,CY121379,CY112915,EU097800,DQ487328,FJ970925,EU116037,EU399757,EU798899,EU798898,GU937745,AF455723,JQ290170,JQ290186,JQ070774,JQ070790,JQ070782,JQ070798,JN638731,JN655554,JN655539,JN866184,EU798904,EU798900,EU798901,EU798903,EU798907,EU798906,EU798902,EU798905,CY101132,DQ139327,AB434414,DQ280260,CY022347,CY022403,GU086047,GU086031,GU086039,EU502891,GU086015,GU086023,GU086007,CY022483,CY039931,CY022435,CY039923,AB434406,AB434398,DQ280198,CY024939,CY044371,CY130124,CY039997,DQ280257,DQ280256,DQ280253,CY058485,GU086063,AB434366,FJ200418,FJ200426,AB434334,CY009378,AJ311460,GU086071,GU086055,CY020507,CY077932,CY010586,FJ415614,CY116378,JX860680,JX963592,JX860696,JX963607,JX860688,AB434286,AB434294,AB434326,AB434318,AF389116,DQ874874,CY033583,CY009450,EF467819,AB434358,AB434374,AB256744,CY061739,HM568094,CY057956,JN375037,JN375038,JN375040,JN375041,JN375042,JN375043,JN375044,JN375045,JN375046,JN375048,JN375049,CY014662,CY087782,CY130052,EU743133,CY024752,DQ870886,DQ870892,JN244202,JN244203,CY022659,CY025131,CY025123,CY077648,CY067676,CY005689,GU186783,CY014997,CY014715,GU051859,CY067668,AF285891,AY616765,JQ906568,AF348173,AF348172,CY015107,CY014669,CY130060,CY087830,CY095583,KC815873,KC815865,CY021403,KC815849,KC815857,GU052208,CY014685,EU743439,GU060480,HQ244413,CY005558,CY024824,AY648293,AY646084,GQ921320,AY619971,AY619963,CY117241,HQ541734,CY005630,CY087758,CY130036,CY014655,GU053169,CY014792,CY116841,M25926,CY130004,CY022683,GU052944,CY130084,GU052221,EU182314,AY619955,CY014847,M25925,CY005912,CY022643,HQ244405,JF789600,EU743193,CY089643,CY014700,AF251405,AF251429,CY095673,AJ404635,AF258817,AF250477,AJ404634,AF258816,AF046094,GU052140,AF046085,AF084264,AF036362,AJ404633,AF258819,GU052148,AF258818,AF115292,AB434302,AB434310,GU052696,CY006041,GU052680,FJ610100,AB434342,AF144301,DQ067443,JQ356888,JQ356889,EPI440699,EPI439489,KC885961,KF001508,AGJ51960,AGJ72859,EPI439501,EPI440683,KC464596,JN869529,CY025259,CY022992,HM172404,CY034718,AY737294,AY653199,HM172388,HM172390,AY684704,CY094813,CY036717,CY014419,CY014323,CY014176,AY950275,AY950276,AY950274,AY609310,CY036709,CY029481,AB450522,AY737287,DQ138152,CY034204,CY034212,CY036701,DQ366328,AY950272,DQ138156,DQ138158,CY028706,CY029943,CY028714,CY028722,JN375050,JN375051,JN375047,GQ168860,CY061731,CY061723,GQ168854,JN375034,JN375033,JN375035,HM567974,HM567966,HM567998,JN375031,JN375032,JN375030,GQ117075,FJ984353,CY041619,HM568078,HM567958,HM567982,HM568014,HM568038,HM568030,GQ168884,GQ396747,GQ200234,GQ168875,GQ457491,GQ168867,GQ168864,GQ338347,GQ168887,GQ160544,GQ377081,GQ377083,GQ117088,GQ117046,GQ117026,JF915189,GQ377049,FJ966080,FJ966958,KC781717,FJ966965,KC781787,CY121686,GQ323558,FJ966978,FJ969531,KC780064,FJ984367,GQ377054,GQ303345,GQ339879,GQ149652,GQ463207,GQ149675,GQ379819,GQ162176,GQ162186,GQ339878,GQ149683,GQ162167,GQ149633,FJ998226,JN617972,JN617973,CY061803,CY061771,CY061787,CY061795,M25933,GU052017,CY130092,AB274964,EF015557,AY340085,AB438939,AY340083,EU743424,GU052258,CY116897,CY101488,CY127259,CY005310,CY101561,EU743431,CY033175,EU268217,EF467810,AB450518,AB450519,AB450521,CY016929,CY017033,EU148394,EU148450,EU148410,EU148362,EU148378,EU148386,EU148402,CY016913,CY016945,EU148442,CY016290,CY017185,CY016953,EU148370,CY020651,CY020659,CY016905,HM172479,CY020699,CY020707,CY020715,CY020683,CY016298,CY020667,CY016937,EU277839,CY016306,CY021395,CY020675,CY016282,CY014294,GQ122443,CY014499,CY019398,CY019406,CY019430,CY019414,CY019422,CY014331,CY014513,CY014327,GQ122449,GQ122437,GQ122455,EU743092,JN244194,JN596873,CY032259,AY210009,CY033479,CY036821,CY031601,CY125852,M25935,CY032243,CY110939,CY110947,CY005821,GU052386,CY015079,JN244197,GQ176130,CY014677,GU052195,DQ997322

**Accession number of PB1_F2 segment:**

CY033583,CY009450,EF467819,CY064972,CY058485,CY022347,CY022403,CY044371,CY130124,CY039997,CY024939,CY130052,CY014662,CY087782,CY015107,FJ200418,FJ200426,CY110939,CY110947,CY025259,CY022992,CY010586,CY009378,CY020507,CY077932,FJ415614,CY116378,JQ906568,CY034718,CY014294,CY016929,CY017033,CY016953,CY016913,CY016290,CY017185,CY016945,CY014419,CY014323,CY014176,CY029481,EF619976,CY016937,CY020651,CY016905,CY020659,CY021395,CY016306,CY016298,CY020667,CY020683,CY020675,CY020699,CY020707,CY020715,EU277839,CY016282,EF593100,EF467810,CY019398,CY019406,CY019414,CY019422,CY019430,CY014327,CY014331,CY014513,CY014499,CY036717,CY094813,CY034212,CY036701,CY036709,CY034204,CY029943,GQ122443,CY028706,CY028714,CY028722,CY033175,GQ122449,GQ122437,GQ122455,EPI440699,JQ356889,JQ356888,EPI439489,EPI439501,EPI440683,CY005558,CY005630,AB274964,CY101132,CY014715,CY022643,HQ244405,CY116897,CY077648,CY095583,KC815865,KC815873,KC815857,KC815849,CY021403,CY022683,CY022659,CY025131,CY025123,GU060480,HQ244413,JF789600,AB438939,CY024752,CY014997,FJ374513,CY061739,JN866184,JQ070798,JN655554,JN638731,JN992755,JQ070782,JQ070790,JQ070766,JQ290170,JQ290186,JQ070774,JN655539,EU798904,EU798900,EU798901,EU798903,EU798902,EU798907,EU798906,CY095673,EU798898,EU798899,FJ970925,CY039093,CY035028,EU620766,CY033652,CY114387,CY034122,CY121379,CY112915,DQ487328,CY000807,CY039085,CY112891,CY088481,CY112939,CY114379,CY002790,CY009306,CY002102,CY003726,CY003742,CY113683,CY033612,CY113371,CY121315,CY112402,CY121299,CY014685,EU743092,EU743193,CY005689,HQ541734,CY067668,CY067676,CY024824,CY006041,FJ610100,EU182314,EU743424,EU743431,EU743439,GQ921320,GQ176130,CY014677,CY005821,JN596873,CY032259,CY033479,CY032243,CY036821,CY031601,CY125852,CY130084,CY015079,CY127259,CY117241,CY089643,CY014700,CY130092,CY116841,CY130004,CY087758,CY014655,CY130036,CY087830,CY130060,CY014669,EU743133,CY014792,CY101488,CY014847,CY005912,CY005310,CY101561

**Accession number of PB2 segment:**

EPI440682,EPI440690,EPI439495,KF001507,EPI439504,CY089644,CY014701,CY130093,JN244111,CY014998,GU186784,CY015058,CY005405,DQ997323,CY014670,CY087831,CY130061,KC815864,CY130053,CY005831,GU051972,CY067675,DQ366327,CY022684,CY022660,CY025124,CY025132,CY087759,CY130037,GU060479,HQ244412,GU052222,CY130085,CY022644,CY024825,HQ541735,EU743194,CY015108,AY619962,AY619970,EU084946,CY101489,CY101562,AY342413,EF015558,EU743440,AY653193,AF285892,EF597480,CY015120,EU743425,EU743432,CY005690,EU743134,CY014793,CY005311,AB438938,AY342414,GU052209,CY014686,CY005822,GU052196,GQ176129,CY014678,CY014848,CY057957,GQ200233,GQ200260,GQ200222,FJ998206,GQ200240,CY041620,HM567965,HM567997,GQ117076,GQ200263,FJ984351,GQ377076,GQ200258,GQ200254,GQ200232,GQ377099,GQ377080,GQ160545,GQ168879,GQ117089,GQ117047,GQ117027,JF915190,FJ966079,FJ969516,FJ966955,KC781783,CY121687,FJ969530,FJ966976,FJ984387,EPI439508,EPI439508,CY087783,AB434413,DQ280197,DQ139325,GU086038,GU086046,EU502892,GU086014,GU086022,GU086006,GU086030,CY022404,CY022348,CY024940,CY044372,CY130125,AB434405,AB434397,CY022484,CY022436,CY039924,CY039932,AB256743,AF222816,CY058484,AF251402,M73521,AY209935,JN596872,CY032260,CY031602,CY036822,CY125853,M73524,AF348171,AF348170,CY033480,CY032244,AF258842,AF251394,CY009307,GU086134,EU273802,EU273805,CY039094,CY035029,CY002791,CY114380,EU620765,CY033653,CY114388,CY034123,CY112940,CY088480,FJ970926,CY000808,EU273803,EU273804,AF037416,CY112916,CY121380,DQ486029,CY039086,KC780061,GQ377051,FJ984365,GQ162180,GQ379815,GQ200202,CY098505,GQ162168,GQ162198,GQ200201,GQ149632,CY097778,JN617971,FJ969525,GQ168885,AF455731,CY095672,EU798922,EU798927,EU798926,EU798920,EU798924,EU798921,EU798923,EU798925,EU798918,EU798919,EU116038,DQ874873,CY033584,CY009451,AF389115,EF467818,HM172483,CY014293,HM172406,DQ997225,CY014420,CY014520,CY014175,CY014322,CY014332,CY014500,CY014329,AY609309,GQ122442,GQ122448,GQ122436,GQ122454,AY737286,CY029480,HM172430,CY094814,HM172428,AB450506,AB450502,CY036718,AY684703,AB450505,CY034213,AY770084,EU268216,EF467807,CY028723,AB450503,EF593099,AY950281,AY950282,AY950279,AY950283,DQ138177,CY036702,CY036710,CY028707,DQ138178,CY034205,DQ138173,CY029944,CY028715,CY033176,CY015080,CY025260,CY022993,CY101133,AY619954,AF144300,EU743093,CY110938,CY110946,JQ356886,JQ356885,JN869528,EPI439488,KC885962,AGJ51961,EPI440698,KC464595,AGJ72858,GU051860,CY005913,HQ244404,CY067667,JQ906564,CY116898,GU053116,CY127260,GQ921319,CY117242,AY616766,AY648294,CY024753,CY077649,CY005631,CY005559,CY116842,CY130005,JF789599,CY014716,AB274963,KC815848,KC815872,KC815856,CY021404,CY095584,AB434325,AB434317,AB434301,AB434309,AB434333,AB434373,AB434341,AB434365,AB434293,AB434285,CY116377,AJ311459,CY112892,CY002103,AF037417,CY113684,CY033613,U71135,CY003743,CY003727,CY113372,CY121316,CY121300,CY112403,M73515,CY020508,CY010587,AF251410,AF251434,EU015993,GU086078,EU399758,JQ070797,JN638726,JQ070777,JQ070789,JN655532,JQ290169,JQ290185,FJ374512,JN375000,JN375001,JN375002,JN375003,JN375004,JN375006,JN375007,JN375008,JN375009,JN375005,JN375011,JN375012,JN375013,JN375014,JN375015,GQ396746,JN374995,JN374996,JN374994,KC781718,FJ966963,HM568093,CY061802,CY061770,CY061786,CY061794,JN374998,JN374997,JN374999,HM568029,HM568013,HM568077,HM568037,HM567957,HM567981,GQ162177,HM567973,AF258838,AF036363,AF084261,GU052141,AF258837,AF115290,AJ404632,GU052149,AJ404630,AF258835,AJ404631,AF258836,AF250476,FJ200417,FJ200425,AB434357,GU086062,GU086070,GU086054,FJ415615,JX860689,JX963608,JX860681,JX860697,JX963593,CY009379,CY005844,GU052681,GU052697,FJ610101,JN244108,AY737293,M73523,CY016307,CY016299,CY020668,CY021396,CY020684,CY020676,CY020700,CY020708,CY020716,EU277840,CY016283,DQ529292,CY034717,CY016906,CY020652,CY020660,EU148363,EU148379,EU148403,EU148395,EU148371,CY016930,CY017034,CY016914,CY016954,CY016946,CY016938,CY017186,CY016291,EU148451,EU148411,EU148443
