# Supplementary material for: Novel approach for identification of influenza virus host range and zoonotic transmissible sequences by determination of host-related associative positions in viral genome segments
Source: BMC Genomics. 2016 Nov 16;17:925. doi: 10.1186/s12864-016-3250-9 (PMC5112743; doi:10.1186/s12864-016-3250-9)
Supplement: Additional file 2: — Includes whole references of strains that were used in this study. (DOCX 72 kb) [file 12864_2016_3250_MOESM2_ESM.docx]

| **Strain** | **References** |
| --- | --- |
| A/chicken/Italy/312/1997 | [[1](#_ENREF_1), [2](#_ENREF_2)] |
| A/FPV/Weybridge | [[3](#_ENREF_3), [4](#_ENREF_4)] |
| A/duck/Chiba/11/2007 | [[5](#_ENREF_5)] |
| A/duck/Czech/1956 | [[6](#_ENREF_6)] |
| A/chicken/California/431/2000 | [[7](#_ENREF_7), [8](#_ENREF_8)] |
| A/chicken/Puebla/28159-474/1995 | [[9](#_ENREF_9)] |
| A/mallard/New York/6874/1978 | [[10](#_ENREF_10)] |
| A/duck/Tsukuba/168/2005 | [[11](#_ENREF_11)] |
| A/mallard/Gurjev/263/1982 | [[12](#_ENREF_12)] |
| A/duck/Australia/341/1983 | [[1](#_ENREF_1), [13](#_ENREF_13)] |
| A/duck/Tsukuba/41/2005 | [[5](#_ENREF_5)] |
| A/chicken/Nigeria/1071-1/2007 | [[14](#_ENREF_14)] |
| A/duck/Hong Kong/3174/99 | [[15](#_ENREF_15)] |
| A/Guinea Fowl/Italy/330/97 | [[16](#_ENREF_16)] |
| A/goose/Hong Kong/W217/97 | [[15](#_ENREF_15)] |
| A/GuineaFowl/HongKong/NT184/03 | [[17](#_ENREF_17)] |
| A/chicken/Nigeria/1071-5/2007 | [[14](#_ENREF_14)] |
| A/chicken/Nigeria/1071-29/2007 | [[10](#_ENREF_10)] |
| A/duck/Germany/1972 | [[18](#_ENREF_18)] |
| A/chicken/Nigeria/1071-4/2007 | [[10](#_ENREF_10)] |
| A/chicken/Nigeria/1071-3/2007 | [[10](#_ENREF_10)] |
| A/chicken/Nigeria/1071-10/2007 | [[10](#_ENREF_10)] |
| A/chicken/Nigeria/1071-7/2007 | [[10](#_ENREF_10)] |
| A/parrot/Northern Ireland/VF-73-67/73 | [[19](#_ENREF_19)] |
| A/duck/Tsukuba/212/2006 | [[5](#_ENREF_5)] |
| A/duck/Tsukuba/63/2005 | [[11](#_ENREF_11)] |
| A/mallard/Tennessee/11464/85 | [[20](#_ENREF_20)] |
| A/duck/Memphis/928/1974 | [[10](#_ENREF_10)] |
| A/duck/Bavaria/1/77 | [[20](#_ENREF_20)] |
| A/chicken/Nigeria/1071-30/2007 | [[14](#_ENREF_14)] |
| A/GuineaFowl/HongKong/NT101/03 | [[17](#_ENREF_17)] |
| A/chicken/Nigeria/1071-9/2007 | [[14](#_ENREF_14)] |
| A/Chicken/HongKong/NT142/03 | [[17](#_ENREF_17)] |
| A/turkey/Oregon/1971 | [[21](#_ENREF_21)] |
| A/duck/New Zealand/31/1976 | [[22](#_ENREF_22)] |
| A/duck/Hong Kong/1037-2/98 | [[15](#_ENREF_15)] |
| A/duck/Alberta/78/1976 | [[10](#_ENREF_10)] |
| A/Chicken/Italy/367/97 | [[16](#_ENREF_16)] |
| A/guillemot/Sweden/3/00 | [[23](#_ENREF_23)] |
| A/turkey/England/647/77 | [[19](#_ENREF_19)] |
| A/duck/Tsukuba/30/2007 | [[11](#_ENREF_11)] |
| A/chicken/Puebla/8623-607/1994 | [[24](#_ENREF_24)] |
| A/Chicken/HongKong/WF126/03 | [[17](#_ENREF_17)] |
| A/turkey/Minnesota/1661/1981 | [[20](#_ENREF_20)] |
| A/Goose/Guangdong/1/96 | [[25](#_ENREF_25)] |
| A/mallard/ALB/124/1991 | [1] |
| A/teal/Italy/3931-38/2005 | [[26](#_ENREF_26)] |
| A/duck/Hubei/10/2010 | [[27](#_ENREF_27)] |
| A/turkey/Ontario/6118/1968 | [1] |
| A/turkey/Massachusetts/3740/1965 | [26] |
| A/chicken/Nigeria/1047-30/2006 | [26] |
| A/chicken/Chis/15224/1997 | [1] |
| A/chicken/Nigeria/1047-62/2006 | [[26](#_ENREF_26)] |
| A/Duck/Alberta/60/1976 | [[28](#_ENREF_28)] |
| A/duck/England/1/1956 | [1] |
| A/mallard/Alberta/353/1988 | [1] |
| A/duck/Hong Kong/273/1978 | [26] |
| A/duck/Czechoslovakia/1956 | [28] |
| A/chicken/Nigeria/1047-8/2006 | [26] |
| A/chicken/Nigeria/1047-34/2006 | [26] |
| A/chick/Pennsylvania/1/1983 | [[29](#_ENREF_29)] |
| A/ruddy turnstone/New Jersey/260/1990 | [26] |
| A/duck/Hubei/2/2010 | [[27](#_ENREF_27)] |
| A/turkey/Wisconsin/1/1966 | [1] |
| A/chicken/Laos/P0171/2007 | [[30](#_ENREF_30)] |
| A/chicken/Yokohama/aq144/2001 | [[31](#_ENREF_31)] |
| A/herring gull/DE/677/1988 | [[18](#_ENREF_18)] |
| A/Teal/Hong Kong/W312/97 | [[32](#_ENREF_32)] |
| A/duck/Italy/775/2004 | [26] |
| A/fowl/Dobson/1927 | [1] |
| A/chicken/Nigeria/1047-54/2006 | [26] |
| A/duck/Hong Kong/24/1976 | [1] |
| A/chicken/Germany/n/1949 | [[33](#_ENREF_33)] |
| A/duck/Ukraine/1/63 | [[34](#_ENREF_34)] |
| A/red knot/Delaware/541/1988 | [26] |
| A/mallard/Ontario/56/1976 | [26] |
|  |  |
|  |  |
| A/turkey/Ontario/7732/1966 | [[35](#_ENREF_35)] |
| A/duck/Manitoba/1/1953 | [[36](#_ENREF_36)] |
| A/teal/Iceland/29/1980 | [[36](#_ENREF_36)] |
| A/Chicken/Hong Kong/G23/97 | [[37](#_ENREF_37)] |
| A/duck/england/1/1962 | [[36](#_ENREF_36)] |
| A/FPV/Rostock/1934 | [[38](#_ENREF_38)] |
| A/swine/Italy/2/1979 | [36] |
| A/Chukkar/MN/14951-7/1998 | [[39](#_ENREF_39)] |
| A/turkey/Ontario/7732/1966 | [36] |
| A/duck/NY/191255-59/2002 | [39] |
| A/swine/Ontario/42729A/01 | [[40](#_ENREF_40)] |
| A/duck/Australia/749/1980 | [[41](#_ENREF_41)] |
| A/turkey/Minnesota/833/1980 | [[1](#_ENREF_1)] |
| A/swine/Saskatchewan/18789/02 | [[40](#_ENREF_40)] |
| A/swine/Ontario/01911-1/99 | [[42](#_ENREF_42)] |
| A/swine/Ontario/K01477/01 | [[40](#_ENREF_40)] |
| A/duck/Hong Kong/7/1975 | [[43](#_ENREF_43)] |
| A/Swine/Ohio/891/01 | [[44](#_ENREF_44)] |
| A/Swine/Nebraska/209/98 | [[45](#_ENREF_45)] |
| A/Swine/Minnesota/593/99 | [[45](#_ENREF_45)] |
| A/mallard/New York/6750/1978 | [[18](#_ENREF_18)] |
| A/Mallard/NY/6750/1978 | [[46](#_ENREF_46)] |
| A/chicken/Pennsylvania/1/1983 | [[47](#_ENREF_47)] |
| A/duck/AUS/749/1980 | [[47](#_ENREF_47)] |
|  |  |
|  |  |
|  |  |
| A/Guangdong/07/2005 | [[48](#_ENREF_48)] |
| A/Beijing/262/95 | [[49](#_ENREF_49)] |
| A/California/05/2009(H1N1) | [[50](#_ENREF_50)] |
| A/NT/60/68 | [[51](#_ENREF_51)] |
| A/Kansas/13/2009 | [[52](#_ENREF_52)] |
| A/USSR/90/77 | [[53](#_ENREF_53)] |
| A/Solomon Islands/3/2006 | [[54](#_ENREF_54)] |
| A/Zhejiang/DTID-ZJU01/2013 | [[55](#_ENREF_55)] |
| A/Ontario/1252/2007 | [[56](#_ENREF_56)] |
| A/Hong Kong/482/97 | [[57](#_ENREF_57)] |
| A/Beijing/32/1992 | [[58](#_ENREF_58)] |
| A/Hong Kong/156/97 | [[59](#_ENREF_59)] |
| A/Moscow/10/99 | [[60](#_ENREF_60)] |
| A/Hong Kong/5/83 | [[61](#_ENREF_61)] |
| A/Krasnodar/101/1959 | [[18](#_ENREF_18)] |
| A/Hong Kong/481/97 | [[62](#_ENREF_62)] |
| A/New York/141/1999 | [[63](#_ENREF_63)] |
|  |  |
|  |  |
| A/Nanchang/933/95 | [[64](#_ENREF_64)] |
| A/mallard/Alberta/279/98 | [[65](#_ENREF_65)] |
| a/swine/new jersey/11/76 | [[61](#_ENREF_61)] |
| A/Idaho/4/95 | [64] |
| A/Bayern/7/95 | [[66](#_ENREF_66)] |
| A/swine/Belgium/1/83 | [[20](#_ENREF_20)] |
| A/Beijing/353/89 | [[60](#_ENREF_60)] |
| A/Shiga/25/97 | [[60](#_ENREF_60)] |
| A/Beijing/32/92 | [[64](#_ENREF_64)] |
| A/duck/Hokkaido/83/2004 | [[67](#_ENREF_67)] |
| A/duck/Hokkaido/W73/2007 | [[67](#_ENREF_67)] |
| A/Fort Monmouth/1/47-MA | [[68](#_ENREF_68)] |
| A/Swine/England/195852/92 | [[69](#_ENREF_69)] |
| A/duck/Alberta/35/76 | [[20](#_ENREF_20)] |
| A/Duck/Hokkaido/8/80 | [[70](#_ENREF_70)] |
| A/duck/Zhejiang/12/2011 | [[71](#_ENREF_71)] |
| A/Puerto Rico/8/34 | [[72](#_ENREF_72)] |
| A/Puerto Rico/8/34/Mount Sinai | [[73](#_ENREF_73)] |
| A/gull/Maryland/704/1977 | [[1](#_ENREF_1)] |
| A/Swine/Colorado/1/77 | [[10](#_ENREF_10)] |
|  |  |
|  |  |
|  |  |
| A/chicken/Nakhon Sawan/NIAH01502/2004 | [[74](#_ENREF_74)] |
| A/chicken/Nonthaburi/NIAH2879/2004 | [[74](#_ENREF_74)] |
| A/chicken/Samutprakan/NIAH6604/2004 | [[74](#_ENREF_74)] |
| A/Chicken/Hong Kong/y388/97 | [[75](#_ENREF_75)] |
| A/chicken/Viet Nam/1/2004 | [[76](#_ENREF_76)] |
| A/chicken/Netherlands/03010132/03 | [[77](#_ENREF_77)] |
| A/Mallard/Sweden/91/02 | [[78](#_ENREF_78)] |
| A/ruddy turnstone/DE/1538/2000 | [[79](#_ENREF_79)] |
| A/chicken/Hubei/14/2004 | [[76](#_ENREF_76)] |
| A/Chicken/Hong Kong/220/97 | [[80](#_ENREF_80)] |
| A/mallard/Netherlands/12/00 | [[81](#_ENREF_81)] |
| A/Netherlands/33/03 | [81] |
| A/Duck/Hong Kong/p46/97 | [[75](#_ENREF_75)] |
| A/chicken/Suphanburi/NIAH7618/2004 | [74] |
| A/chicken/Thailand/CH-2/2004 | [[82](#_ENREF_82)] |
| A/Chicken/Hong Kong/258/97 | [[83](#_ENREF_83)] |
| A/turkey/Turkey/1/2005 | [[84](#_ENREF_84)] |
| A/chicken/Niger/2130-7/2006 | [[85](#_ENREF_85)] |
| A/Goose/Hong Kong/w355/97 | [75] |
| A/Chicken/Hong Kong/786/97 | [[86](#_ENREF_86)] |
| A/chicken/Niger/2130-8/2006 | [85] |
| A/chicken/British Columbia/CN-7/2004 | [[87](#_ENREF_87)] |
| A/chicken/Angthong/NIAH8334/2004 | [74] |
| A/chicken/Nakhon Sawan/NIAH01503/2004 | [74] |
| A/Chicken/Hong Kong/915/97 | [86] |
| A/Chicken/Hong Kong/728/97 | [86] |
| A/chicken/Yunnan/207/2004 | [[88](#_ENREF_88)] |
| A/duck/East Java/UT1046/2004 | [[89](#_ENREF_89)] |
| A/chicken/Kalasin/NIAH316/2004 | [74] |
| A/chicken/Sudan/1784-7/2006 | [[90](#_ENREF_90)] |
| A/chicken/Egypt/2253-1/2006 | [90] |
| A/ruddy turnstone/Delaware Bay/220/1995 | [[26](#_ENREF_26)] |
| A/quail/Yogjakarta/UT1023/2004 | [89] |
| A/chicken/Italy/4746/1999 | [[26](#_ENREF_26)] |
| A/Chicken/Guangdong/174/04 | [[91](#_ENREF_91)] |
| A/chicken/Vietnam/24/2004 | [26] |
| A/blue-winged teal/Ohio/566/2006 | [26] |
| A/chicken/Gansu/44/2004 | [[92](#_ENREF_92)] |
| A/turkey/Egypt/2253-2/2006 | [[90](#_ENREF_90)] |
| A/turkey/Ivory Coast/4372-3/2006 | [[90](#_ENREF_90)] |
| A/chicken/Italy/1082/1999 | [26] |
| A/chicken/Guangdong/191/04 | [91] |
| A/chicken/British Columbia/GSC_human_B/04 | [[93](#_ENREF_93)] |
| A/chicken/East Kalimantan/UT1035/2004 | [89] |
| A/turkey/Ivory Coast/4372-2/2006 | [[90](#_ENREF_90)] |
| A/GSC_chicken_B/British Columbia/04 | [93] |
| A/chicken/Suphanburi/NIAH7540/2004 | [74] |
| A/chicken/Netherlands/1/03 | [81] |
| A/chicken/Sudan/2115-12/2006 | [[90](#_ENREF_90)] |
| A/chicken/Guangxi/12/2004 | [[94](#_ENREF_94)] |
| A/duck/Egypt/2253-3/2006 | [[90](#_ENREF_90)] |
| A/duck/Niger/914/2006 | [26] |
| A/quail/Yogjakarta/UT1075/2004 | [89] |
| A/chicken/Hunan/41/2004 | [[92](#_ENREF_92)] |
| A/chicken/Jiangxi/25/2004 | [92] |
| A/chicken/NaraThiwat/NIAH1703/2004 | [74] |
| A/chicken/Hubei/327/2004 | [[95](#_ENREF_95)] |
| A/chicken/British Columbia/04 | [93] |
| A/chicken/Italy/4789/1999 | [26] |
| A/quail/Italy/4992/1999 | [26] |
| A/blue-winged teal/Guatemala/CIP049-01/2008 | [[96](#_ENREF_96)] |
| A/Netherlands/219/03 | [81] |
| A/turkey/Minnesota/1/1988 | [[1](#_ENREF_1)] |
| A/chicken/Guangdong/178/04 | [91] |
| A/Thailand/16/2004 | [[97](#_ENREF_97)] |
| A/Hong Kong/1073/99 | [[98](#_ENREF_98)] |
| A/Hong Kong/1074/99 | [98] |
| A/chicken/Sudan/2115-9/2006 | [[90](#_ENREF_90)] |
| A/chicken/Nigeria/957-20/2006 | [26] |
| A/chicken/Kohn Kaen/NIAH330/2004 | [74] |
| A/Hong Kong/1/68 | [[99](#_ENREF_99)] |
| A/turkey/Ivory Coast/4372-4/2006 | [90] |
| A/chicken/Sudan/1784-8/2006 | [90] |
| A/chicken/Sudan/1784-10/2006 | [90] |
| A/chicken/Nigeria/641/2006 | [26] |
| A/blue-winged teal/Guatemala/CIP049-02/2008 | [96] |
| A/chicken/Anhui/39/2004 | [[92](#_ENREF_92)] |
|  |  |
|  |  |
|  |  |
| A/New York/08/2009 | [[100](#_ENREF_100)] |
| A/swine/Chonburi/06CB2/2006 | [[101](#_ENREF_101)] |
| A/Swine/Wisconsin/238/97 | [[102](#_ENREF_102)] |
| A/Ohio/02/2007 | [[103](#_ENREF_103)] |
| A/Swine/Wisconsin/235/97 | [102] |
| A/Texas/06/2009 | [[50](#_ENREF_50)] |
| A/California/05/2009 | [[104](#_ENREF_104)] |
| A/swine/Chonburi/05CB1/2005 | [101] |
| A/Thailand/271/2005 | [[105](#_ENREF_105)] |
| A/swine/Thailand/HF6/2005 | [[106](#_ENREF_106)] |
| A/Swine/Wisconsin/125/97 | [102] |
| A/Ohio/01/2007 | [[103](#_ENREF_103)] |
| A/Texas/07/2009 | [[107](#_ENREF_107)] |
| A/Texas/09/2009 | [100] |
| A/swine/Saraburi/NIAH13021/2005 | [[108](#_ENREF_108)] |
| A/swine/Chachoengsao/NIAH587/2005 | [101] |
| A/Mexico/4482/2009 | [100] |
| A/swine/Ratchaburi/NIAH874/2005 | [108] |
| A/California/04/2009 | [100] |
| A/swine/Chonburi/NIAH589/2005 | [108] |
| A/Brisbane/59/2007 | [[109](#_ENREF_109)] |
| A/New York/20/2009 | [100] |
| A/swine/Ratchaburi/NIAH1481/2000 | [108] |
| A/Colorado/03/2009 | [100] |
| A/Ohio/07/2009 | [100] |
| A/Texas/04/2009 | [100] |
| A/swine/Chachoengsao/2003 | [108] |
| A/swine/Zhejiang/1/2007 | [[110](#_ENREF_110)] |
| A/New York/31/2009 | [100] |
| A/Texas/05/2009 | [100] |
| A/swine/Ratchaburi/NIAH59/2004 | [108] |
| A/Swine/Zhejiang/1/2004 | [[111](#_ENREF_111)] |
| A/swine/Ratchaburi/NIAH550/2003 | [108] |
| A/Texas/08/2009 | [100] |
| A/swine/Chonburi/NIAH9469/2004 | [108] |
| A/swine/Shanghai/1/2007 | [110] |
| A/New York/23/2009 | [100] |
| A/Arizona/02/2009 | [100] |
| A/swine/Guangxi/13/2006 | [110] |
| A/swine/Nakhon pathom/NIAH586-1/2005 | [108] |
| A/swine/Chonburi/NIAH977/2004 | [108] |
| A/swine/Shanghai/1/2005 | [[112](#_ENREF_112)] |
| A/South Carolina/09/2009 | [100] |
|  |  |
|  |  |
|  |  |
| A/swine/Guangdong/211/2006 | [[113](#_ENREF_113)] |
| A/swine/Guangdong/811/2006 | [113] |
| A/swine/Korea/JL01/2005 | [[114](#_ENREF_114)] |
| A/swine/Ukkel/1/1984 | [[10](#_ENREF_10)] |
| A/swine/Hokkaido/2/81 | [[108](#_ENREF_108)] |
| A/swine/Shaanxi/s2/2012 | [[115](#_ENREF_115)] |
| A/swine/Ehime/1/80 | [[116](#_ENREF_116)] |
| A/SW/MN/34893/01 | [[117](#_ENREF_117)] |
| A/Swine/Wisconsin/458/98 | [[102](#_ENREF_102)] |
| A/swine/Korea/PZ7/2006 | [114] |
| A/swine/England/690421/95 | [[118](#_ENREF_118)] |
| A/swine/Netherlands/3/80 | [[20](#_ENREF_20)] |
| A/swine/Iowa/15/1930 | [[119](#_ENREF_119)] |
| A/swine/Shaanxi/s1/2011 | [115] |
| A/swine/Shandong/106/2007 | [113] |
| A/swine/Scotland/410440/94 | [118] |
| A/Swine/Wisconsin/464/98 | [102] |
| A/swine/England/438207/94 | [118] |
| A/swine/Korea/CAS08/2005 | [114] |
| A/swine/Korea/Asan04/2006 | [102] |
| A/swine/Shaanxi/s6/2012 | [115] |
| A/swine/Shaanxi/sf/2011 | [115] |
| A/swine/Shaanxi/s3/2012 | [115] |
| A/swine/England/283902/93 | [[69](#_ENREF_69)] |
| A/Swine/Iowa/15/30 | [[116](#_ENREF_116)] |
| A/Swine/Wisconsin/166/97 | [102] |
| A/swine/Korea/JL02/2005 | [114] |
| A/swine/England/17394/96 | [118] |
| A/swine/Korea/PZ14/2006 | [114] |
| A/Swine/Wisconsin/163/97 | [102] |
| A/Swine/Wisconsin/457/98 | [102] |
| A/swine/Korea/JL04/2005 | [114] |
| A/Swine/Wisconsin/164/97 | [102] |
| A/Swine/Wisconsin/168/97 | [102] |
| A/swine/Wisconsin/1/61 | [20] |
| A/swine/Netherlands/12/85 | [[41](#_ENREF_41)] |
| A/Swine/Wisconsin/136/97 | [102] |
| A/swine/Korea/Hongsong2/2004 | [114] |
| A/swine/Korea/CAN01/2004 | [114] |
| A/swine/England/117316/86 | [69] |
| A/swine/Guangdong/Z5/2003 | [[120](#_ENREF_120)] |
| A/swine/England/72685/96 | [118] |
| A/swine/Korea/PZ4/2006 | [114] |
| A/swine/Schleswig-Holstein/1/93 | [69] |
| A/swine/Guangdong/176/2009 | [[121](#_ENREF_121)] |
| A/swine/Guangdong/106/2009 | [121] |
| A/swine/Guangdong/114/2009 | [121] |
| A/swine/Ehime/1/1980 | [108] |
| A/Swine/Indiana/1726/1988 | [[122](#_ENREF_122)] |
| A/swine/Alberta/56626/03 | [[123](#_ENREF_123)] |
| A/Swine/Guangdong/165/06 | [[124](#_ENREF_124)] |
| A/swine/Saitama/1996 | [108] |
| A/Swine/Guangdong/164/06 | [124] |
| A/swine/Ontario/00130/97 | [[45](#_ENREF_45)] |
| A/swine/Anhui/01/2006 | [[125](#_ENREF_125)] |
| A/swine/Chibi/01/2005 | [125] |
| A/swine/Niigata/1/1977 | [108] |
| A/Swine/Guangdong/166/06 | [124] |
| A/Swine/Heilongjiang/1/05 | [124] |
| A/swine/Ille et Vilaine/1455/1999 | [[126](#_ENREF_126)] |
| A/swine/Guangdong/213/2009 | [121] |
| A/swine/Guangdong/221/2009 | [121] |

**References:**

1. Obenauer, J.C., et al., *Large-scale sequence analysis of avian influenza isolates.* Science, 2006. **311**(5767): p. 1576-1580.

2. Duan, L., et al., *Characterization of low-pathogenic H5 subtype influenza viruses from Eurasia: implications for the origin of highly pathogenic H5N1 viruses.* Journal of virology, 2007. **81**(14): p. 7529-7539.

3. Markushin, S., et al., *Nucleotide sequence of RNA segment 7 and the predicted amino sequence of M1 and M2 proteins of FPV/Weybridge (H7N7) and WSN (H1N1) influenza viruses.* Virus research, 1988. **10**(2): p. 263-271.

4. Karginov, V., et al., *[Comparative analysis of primary structure of M-genes in remantadine-resistant and remantadine-sensitive strains of influenza virus A/FPV/Weybridge (H7N7) strains].* Bioorganicheskaia khimiia, 1987. **13**(12): p. 1638-1643.

5. Tsukamoto, K., et al., *SYBR Green Based Real-Time Reverse Transcription PCR for Typing and Subtyping of All Hemagglutinin and Neuraminidase genes of Avian Influenza Viruses and Comparison to Standard Serological Subtyping Tests.* Journal of clinical microbiology, 2011: p. JCM. 01195-11.

6. Wagner, R., M. Matrosovich, and H.-D. Klenk, *Functional balance between haemagglutinin and neuraminidase in influenza virus infections.* Reviews in medical virology, 2002. **12**(3): p. 159-166.

7. Webby, R.J., et al., *Reassortment and interspecies transmission of North American H6N2 influenza viruses.* Virology, 2002. **295**(1): p. 44-53.

8. Woolcock, P., D. Suarez, and D. Kuney, *Low-pathogenicity avian influenza virus (H6N2) in chickens in California, 2000-02.* Avian diseases, 2003. **47**(s3): p. 872-881.

9. Lee, C.-W., D.A. Senne, and D.L. Suarez, *Effect of vaccine use in the evolution of Mexican lineage H5N2 avian influenza virus.* Journal of virology, 2004. **78**(15): p. 8372-8381.

10. Bean, W., et al., *Evolution of the H3 influenza virus hemagglutinin from human and nonhuman hosts.* Journal of virology, 1992. **66**(2): p. 1129-1138.

11. Tsukamoto, K., et al., *Broad detection of diverse H5 and H7 hemagglutinin genes of avian influenza viruses by real-time reverse transcription-PCR using primer and probe sets containing mixed bases.* Journal of clinical microbiology, 2010. **48**(11): p. 4275-4278.

12. Kawaoka, Y., et al., *Molecular characterization of a new hemagglutinin, subtype H14, of influenza A virus.* Virology, 1990. **179**(2): p. 759-767.

13. RÖHM, C., et al., *Characterization of a novel influenza hemagglutinin, H15: criteria for determination of influenza A subtypes.* Virology, 1996. **217**(2): p. 508-516.

14. Monne, I., et al., *Reassortant avian influenza virus (H5N1) in poultry, Nigeria, 2007.* Emerg Infect Dis, 2008. **14**(4): p. 637-640.

15. Chin, P., et al., *Molecular evolution of H6 influenza viruses from poultry in Southeastern China: prevalence of H6N1 influenza viruses possessing seven A/Hong Kong/156/97 (H5N1)-like genes in poultry.* Journal of virology, 2002. **76**(2): p. 507-516.

16. Donatelli, I., et al., *Characterization of H5N2 influenza viruses from Italian poultry.* Journal of General Virology, 2001. **82**(3): p. 623-630.

17. Choi, Y., et al., *Continuing evolution of H9N2 influenza viruses in Southeastern China.* Journal of virology, 2004. **78**(16): p. 8609-8614.

18. Schäffr, J.R., et al., *Origin of the pandemic 1957 H2 influenza A virus and the persistence of its possible progenitors in the avian reservoir.* Virology, 1993. **194**(2): p. 781-788.

19. Banks, J., et al., *Phylogenetic analysis of H7 haemagglutinin subtype influenza A viruses.* Archives of virology, 2000. **145**(5): p. 1047-1058.

20. Ito, T., et al., *Molecular basis for the generation in pigs of influenza A viruses with pandemic potential.* Journal of virology, 1998. **72**(9): p. 7367-7373.

21. Orlich, M., et al., *Structural variation occurring in the hemagglutinin of influenza virus A/turkey/Oregon/71 during adaptation to different cell types.* Virology, 1990. **176**(2): p. 531-538.

22. Donis, R.O., et al., *Distinct lineages of influenza virus H4 hemagglutinin genes in different regions of the world.* Virology, 1989. **169**(2): p. 408-417.

23. Wallensten, A., et al., *Multiple gene segment reassortment between Eurasian and American lineages of influenza A virus (H6N2) in Guillemot (Uria aalge).* Archives of virology, 2005. **150**(8): p. 1685-1692.

24. Garcia, M., et al., *Heterogeneity in the haemagglutinin gene and emergence of the highly pathogenic phenotype among recent H5N2 avian influenza viruses from Mexico.* Journal of General Virology, 1996. **77**(7): p. 1493-1504.

25. Xu, X., et al., *Genetic characterization of the pathogenic influenza A/Goose/Guangdong/1/96 (H5N1) virus: similarity of its hemagglutinin gene to those of H5N1 viruses from the 1997 outbreaks in Hong Kong.* Virology, 1999. **261**(1): p. 15-19.

26. Simonsen, L., et al., *The niaid influenza genome sequencing project*, in *National Institute of Allergy and Infectious Diseases, NIH*. 2008, Springer. p. 109-113.

27. Yao, Y., et al., *Characterization of low-pathogenic H6N6 avian influenza viruses in central China.* Archives of virology, 2013. **158**(2): p. 367-377.

28. Nobusawa, E., et al., *Comparison of complete amino acid sequences and receptor-binding properties among 13 serotypes of hemagglutinins of influenza A viruses.* Virology, 1991. **182**(2): p. 475-485.

29. Ohuchi, M., et al., *Mutations at the cleavage site of the hemagglutinin alter the pathogenicity of influenza virus A/chick/Penn/83 (H5N2).* Virology, 1989. **168**(2): p. 274-280.

30. Boltz, D.A., et al., *Emergence of H5N1 avian influenza viruses with reduced sensitivity to neuraminidase inhibitors and novel reassortants in Lao People's Democratic Republic.* Journal of General Virology, 2010. **91**(4): p. 949-959.

31. Mase, M., et al., *Characterization of H9N2 influenza A viruses isolated from chicken products imported into Japan from China.* Epidemiology and infection, 2007. **135**(03): p. 386-391.

32. Hoffmann, E., et al., *Characterization of the influenza A virus gene pool in avian species in southern China: was H6N1 a derivative or a precursor of H5N1?* Journal of Virology, 2000. **74**(14): p. 6309-6315.

33. Feldmann, H., et al., *The structure of serotype H10 hemagglutinin of influenza A virus: comparison of an apathogenic avian and a mammalian strain pathogenic for mink.* Virology, 1988. **165**(2): p. 428-437.

34. Saito, T., Y. Kawaoka, and R.G. Webster, *Phylogenetic analysis of the N8 neuraminidase gene of influenza A viruses.* Virology, 1993. **193**(2): p. 868-876.

35. Philpott, M., et al., *Hemagglutinin mutations related to attenuation and altered cell tropism of a virulent avian influenza A virus.* Journal of virology, 1990. **64**(6): p. 2941-2947.

36. Gorman, O., et al., *Evolution of influenza A virus nucleoprotein genes: implications for the origins of H1N1 human and classical swine viruses.* Journal of Virology, 1991. **65**(7): p. 3704-3714.

37. Guan, Y., et al., *Molecular characterization of H9N2 influenza viruses: were they the donors of the “internal” genes of H5N1 viruses in Hong Kong?* Proceedings of the National Academy of Sciences, 1999. **96**(16): p. 9363-9367.

38. Mocke, K. and C. Scholtissek, *Extragenic and intragenic suppression of a transport mutation in the hemagglutinin gene of an influenza A virus as revealed by backcross and sequence determination.* Virology, 1987. **158**(1): p. 112-117.

39. Lee, C.-w., et al., *Characterization of recent H5 subtype avian influenza viruses from US poultry.* Avian pathology, 2004. **33**(3): p. 288-297.

40. Karasin, A.I., et al., *Characterization of avian H3N3 and H1N1 influenza A viruses isolated from pigs in Canada.* Journal of clinical microbiology, 2004. **42**(9): p. 4349-4354.

41. Guan, Y., et al., *Emergence of avian H1N1 influenza viruses in pigs in China.* Journal of virology, 1996. **70**(11): p. 8041-8046.

42. Karasin, A.I., et al., *Isolation and characterization of H4N6 avian influenza viruses from pigs with pneumonia in Canada.* Journal of virology, 2000. **74**(19): p. 9322-9327.

43. Yasuda, J., et al., *Molecular evidence for a role of domestic ducks in the introduction of avian H3 influenza viruses to pigs in southern China, where the A/Hong Kong/68 (H3N2) strain emerged.* Journal of General Virology, 1991. **72**(8): p. 2007-2010.

44. Karasin, A.I., et al., *Genetic characterization of H1N2 influenza A viruses isolated from pigs throughout the United States.* Journal of clinical microbiology, 2002. **40**(3): p. 1073-1079.

45. Karasin, A.I., et al., *Genetic characterization of H3N2 influenza viruses isolated from pigs in North America, 1977–1999: evidence for wholly human and reassortant virus genotypes.* Virus research, 2000. **68**(1): p. 71-85.

46. Naffakh, N., et al., *Genetic analysis of the compatibility between polymerase proteins from human and avian strains of influenza A viruses.* Journal of General Virology, 2000. **81**(5): p. 1283-1291.

47. Kawaoka, Y., C.W. Naeve, and R.G. Webster, *Is virulence of H5N2 influenza viruses in chickens associated with loss of carbohydrate from the hemagglutinin?* Virology, 1984. **139**(2): p. 303-316.

48. Sun, L., et al., *Genetic correlation between H3N2 human and swine influenza viruses.* Journal of Clinical Virology, 2009. **44**(2): p. 141-144.

49. Lee, M.-S. and C.-F. Yang, *Cross-reactive H1N1 antibody responses to a live attenuated influenza vaccine in children: implication for selection of vaccine strains.* Journal of Infectious Diseases, 2003. **188**(9): p. 1362-1366.

50. Peiris, J.M., L.L. Poon, and Y. Guan, *Emergence of a novel swine-origin influenza A virus (S-OIV) H1N1 virus in humans.* Journal of Clinical Virology, 2009. **45**(3): p. 169-173.

51. Huddleston, J. and G. Brownlee, *The sequence of the nucleoprotein gene of human influenza A virus, strain A/NT/60/68.* Nucleic acids research, 1982. **10**(3): p. 1029-1038.

52. Cox, C.M., et al., *Swine influenza virus A (H3N2) infection in human, Kansas, USA, 2009.* Emerging infectious diseases, 2011. **17**(6): p. 1143-1145.

53. Kaverin, N.V., et al., *Intergenic HA–NA interactions in influenza A virus: postreassortment substitutions of charged amino acid in the hemagglutinin of different subtypes.* Virus research, 2000. **66**(2): p. 123-129.

54. Whittle, J.R., et al., *Broadly neutralizing human antibody that recognizes the receptor-binding pocket of influenza virus hemagglutinin.* Proceedings of the National Academy of Sciences, 2011. **108**(34): p. 14216-14221.

55. Chen, Y., et al., *Human infections with the emerging avian influenza A H7N9 virus from wet market poultry: clinical analysis and characterisation of viral genome.* The Lancet, 2013. **381**(9881): p. 1916-1925.

56. Bastien, N., et al., *Parotitis in a child infected with triple-reassortant influenza A virus in Canada in 2007.* Journal of clinical microbiology, 2009. **47**(6): p. 1896-1898.

57. Shaw, M., et al., *Molecular changes associated with the transmission of avian influenza a H5N1 and H9N2 viruses to humans*.* Journal of medical virology, 2002. **66**(1): p. 107-114.

58. Muster, T., et al., *Mucosal model of immunization against human immunodeficiency virus type 1 with a chimeric influenza virus.* Journal of virology, 1995. **69**(11): p. 6678-6686.

59. Gall, A., et al., *Universal primer set for amplification and sequencing of HA0 cleavage sites of all influenza A viruses.* Journal of clinical microbiology, 2008. **46**(8): p. 2561-2567.

60. Smith, D.J., et al., *Mapping the antigenic and genetic evolution of influenza virus.* Science, 2004.

61. Suzuki, Y., *Sialobiology of influenza: molecular mechanism of host range variation of influenza viruses.* Biological and Pharmaceutical Bulletin, 2005. **28**(3): p. 399-408.

62. Hiromoto, Y., et al., *Evolutionary characterization of the six internal genes of H5N1 human influenza A virus.* Journal of General Virology, 2000. **81**(5): p. 1293-1303.

63. Ghedin, E., et al., *Large-scale sequencing of human influenza reveals the dynamic nature of viral genome evolution.* Nature, 2005. **437**(7062): p. 1162-1166.

64. Fitch, W.M., et al., *Long term trends in the evolution of H (3) HA1 human influenza type A.* Proceedings of the National Academy of Sciences, 1997. **94**(15): p. 7712-7718.

65. Hatchette, T.F., et al., *Influenza A viruses in feral Canadian ducks: extensive reassortment in nature.* Journal of General Virology, 2004. **85**(8): p. 2327-2337.

66. Gregory, V., et al., *Human infection by a swine influenza A (H1N1) virus in Switzerland.* Archives of virology, 2003. **148**(4): p. 793-802.

67. Manzoor, R., et al., *Phylogenic analysis of the M genes of influenza viruses isolated from free-flying water birds from their Northern Territory to Hokkaido, Japan.* Virus Genes, 2008. **37**(2): p. 144-152.

68. Smeenk, C.A., et al., *Mutations in the hemagglutinin and matrix genes of a virulent influenza virus variant, A/FM/1/47-MA, control different stages in pathogenesis.* Virus research, 1996. **44**(2): p. 79-95.

69. Brown, I., et al., *Antigenic and genetic analyses of H1N1 influenza A viruses from European pigs.* Journal of General Virology, 1997. **78**(3): p. 553-562.

70. Kida, H., et al., *Antigenic and genetic conservation of H3 influenza virus in wild ducks.* Virology, 1987. **159**(1): p. 109-119.

71. Hai-bo, W., et al., *Sequence and phylogenetic analysis of H7N3 avian influenza viruses isolated from poultry in China in 2011.* Archives of virology, 2012. **157**(10): p. 2017-2021.

72. Meek, K., et al., *Nucleotide changes in sequential variants of influenza virus hemagglutinin genes and molecular structures of corresponding monoclonal antibodies specific for each variant.* Proceedings of the National Academy of Sciences, 1989. **86**(12): p. 4664-4668.

73. de Wit, E., et al., *Efficient generation and growth of influenza virus A/PR/8/34 from eight cDNA fragments.* Virus research, 2004. **103**(1): p. 155-161.

74. Uchida, Y., et al., *Molecular epidemiological analysis of highly pathogenic avian influenza H5N1 subtype isolated from poultry and wild bird in Thailand.* Virus research, 2008. **138**(1): p. 70-80.

75. Zhou, N.N., et al., *Rapid evolution of H5N1 influenza viruses in chickens in Hong Kong.* Journal of virology, 1999. **73**(4): p. 3366-3374.

76. Donis, R.O., *Evolution of H5N1 avian influenza viruses in Asia.* Emerging infectious diseases, 2005. **11**(10).

77. de Wit, E., et al., *Molecular determinants of adaptation of highly pathogenic avian influenza H7N7 viruses to efficient replication in the human host.* Journal of virology, 2010. **84**(3): p. 1597-1606.

78. Munster, V.J., et al., *Mallards and highly pathogenic avian influenza ancestral viruses, northern Europe.* Emerging infectious diseases, 2005. **11**(10): p. 1545-1551.

79. Spackman, E., et al., *Analytical validation of a real-time reverse transcription polymerase chain reaction test for Pan-American lineage H7 subtype avian influenza viruses.* Journal of veterinary diagnostic investigation, 2008. **20**(5): p. 612-616.

80. Suarez, D.L., et al., *Comparisons of highly virulent H5N1 influenza A viruses isolated from humans and chickens from Hong Kong.* Journal of Virology, 1998. **72**(8): p. 6678-6688.

81. Fouchier, R.A., et al., *Avian influenza A virus (H7N7) associated with human conjunctivitis and a fatal case of acute respiratory distress syndrome.* Proceedings of the National Academy of sciences of the United States of América, 2004. **101**(5): p. 1356-1361.

82. Puthavathana, P., et al., *Molecular characterization of the complete genome of human influenza H5N1 virus isolates from Thailand.* Journal of General Virology, 2005. **86**(2): p. 423-433.

83. Claas, E.C., et al., *Human influenza A H5N1 virus related to a highly pathogenic avian influenza virus.* The Lancet, 1998. **351**(9101): p. 472-477.

84. Xiong, X., et al., *Receptor binding by a ferret-transmissible H5 avian influenza virus.* Nature, 2013. **497**(7449): p. 392-396.

85. Terregino, C., et al., *Active surveillance for avian influenza viruses in wild birds and backyard flocks in Northern Italy during 2004 to 2006.* Avian Pathology, 2007. **36**(4): p. 337-344.

86. Matrosovich, M., et al., *The surface glycoproteins of H5 influenza viruses isolated from humans, chickens, and wild aquatic birds have distinguishable properties.* Journal of virology, 1999. **73**(2): p. 1146-1155.

87. Pasick, J., et al., *Intersegmental recombination between the haemagglutinin and matrix genes was responsible for the emergence of a highly pathogenic H7N3 avian influenza virus in British Columbia.* Journal of General Virology, 2005. **86**(3): p. 727-731.

88. Wang, J., et al., *Identification of the progenitors of Indonesian and Vietnamese avian influenza A (H5N1) viruses from southern China.* Journal of virology, 2008. **82**(7): p. 3405-3414.

89. Takano, R., et al., *Phylogenetic characterization of H5N1 avian influenza viruses isolated in Indonesia from 2003–2007.* Virology, 2009. **390**(1): p. 13-21.

90. Cattoli, G., et al., *Highly pathogenic avian influenza virus subtype H5N1 in Africa: a comprehensive phylogenetic analysis and molecular characterization of isolates.* PloS one, 2009. **4**(3): p. e4842.

91. Wan, X.-F., et al., *Genetic characterization of H5N1 avian influenza viruses isolated in southern China during the 2003–04 avian influenza outbreaks.* Archives of virology, 2005. **150**(6): p. 1257-1266.

92. Li, Y., et al., *Continued evolution of H5N1 influenza viruses in wild birds, domestic poultry, and humans in China from 2004 to 2009.* Journal of virology, 2010. **84**(17): p. 8389-8397.

93. Hirst, M., et al., *Novel avian influenza H7N3 strain outbreak, British Columbia.* Emerg Infect Dis, 2004. **10**(12): p. 2192-5.

94. Li, Y., et al., *Detection of Hong Kong 97-like H5N1 influenza viruses from eggs of Vietnamese waterfowl.* Archives of virology, 2006. **151**(8): p. 1615-1624.

95. Zhou, H., et al., *Genome-sequenee analysis of the pathogenic H5N1 avian influenza A virus isolated in China in 2004.* Virus Genes, 2006. **32**(1): p. 85-95.

96. González-Reiche, A.S., et al., *Influenza A viruses from wild birds in Guatemala belong to the North American lineage.* PLoS One, 2012. **7**(3): p. e32873.

97. Chen, L.-M., et al., *Genetic compatibility and virulence of reassortants derived from contemporary avian H5N1 and human H3N2 influenza A viruses.* PLoS Pathog, 2008. **4**(5): p. e1000072.

98. Lin, Y., et al., *Avian-to-human transmission of H9N2 subtype influenza A viruses: relationship between H9N2 and H5N1 human isolates.* Proceedings of the National Academy of Sciences, 2000. **97**(17): p. 9654-9658.

99. Russell, C.A., et al., *The global circulation of seasonal influenza A (H3N2) viruses.* Science, 2008. **320**(5874): p. 340-346.

100. Garten, R.J., et al., *Antigenic and genetic characteristics of swine-origin 2009 A (H1N1) influenza viruses circulating in humans.* science, 2009. **325**(5937): p. 197-201.

101. Chutinimitkul, S., et al., *Genetic characterization of H1N1, H1N2 and H3N2 swine influenza virus in Thailand.* Archives of virology, 2008. **153**(6): p. 1049-1056.

102. Olsen, C., et al., *Virologic and serologic surveillance for human, swine and avian influenza virus infections among pigs in the north-central United States.* Archives of virology, 2000. **145**(7): p. 1399-1419.

103. Shinde, V., et al., *Triple-reassortant swine influenza A (H1) in humans in the United States, 2005–2009.* New England Journal of Medicine, 2009. **360**(25): p. 2616-2625.

104. Forgie, S.E., et al., *Swine outbreak of pandemic influenza A virus on a Canadian research farm supports human-to-swine transmission.* Clinical Infectious Diseases, 2011. **52**(1): p. 10-18.

105. Komadina, N., et al., *Genetic analysis of two influenza A (H1) swine viruses isolated from humans in Thailand and the Philippines.* Virus genes, 2007. **35**(2): p. 161-165.

106. Sreta, D., et al., *Pathogenesis of swine influenza virus (Thai isolates) in weanling pigs: an experimental trial.* Virology journal, 2009. **6**(1): p. 1.

107. Sheu, T., et al., *Update: drug susceptibility of swine-origin influenza A (H1N1) viruses, April 2009.* Morbidity and Mortality Weekly Report, 2009. **58**.

108. Takemae, N., et al., *Genetic diversity of swine influenza viruses isolated from pigs during 2000 to 2005 in Thailand.* Influenza and other respiratory viruses, 2008. **2**(5): p. 181-189.

109. Barr, I., et al., *Adamantane resistance in influenza A (H1) viruses increased in 2007 in South East Asia but decreased in Australia and some other countries.* Antiviral research, 2008. **80**(2): p. 200-205.

110. Yu, H., et al., *Isolation and genetic characterization of avian-like H1N1 and novel ressortant H1N2 influenza viruses from pigs in China.* Biochemical and biophysical research communications, 2009. **386**(2): p. 278-283.

111. Qi, X. and C. Lu, *Genetic characterization of novel reassortant H1N2 influenza A viruses isolated from pigs in southeastern China.* Archives of virology, 2006. **151**(11): p. 2289-2299.

112. Qi, X., B. Pang, and C. Lu, *Genetic characterization of H1N1 swine influenza A viruses isolated in eastern China.* Virus Genes, 2009. **39**(2): p. 193-199.

113. Zhou, N.N., et al., *Genetic reassortment of avian, swine, and human influenza A viruses in American pigs.* Journal of virology, 1999. **73**(10): p. 8851-8856.

114. Pascua, P.N.Q., et al., *Seroprevalence and genetic evolutions of swine influenza viruses under vaccination pressure in Korean swine herds.* Virus research, 2008. **138**(1): p. 43-49.

115. Wang, J.-Y., et al., *Complete genome sequences of six avian-like H1N1 swine influenza viruses from northwestern China.* Genome announcements, 2013. **1**(1): p. e00098-12.

116. Sugita, S., et al., *Molecular evolution of hemagglutinin genes of H1N1 swine and human influenza A viruses.* Journal of molecular evolution, 1991. **32**(1): p. 16-23.

117. Choi, Y.K., et al., *Phylogenetic analysis of H1N2 isolates of influenza A virus from pigs in the United States.* Virus research, 2002. **87**(2): p. 173-179.

118. Brown, I., et al., *Multiple genetic reassortment of avian and human influenza A viruses in European pigs, resulting in the emergence of an H1N2 virus of novel genotype.* Journal of General Virology, 1998. **79**(12): p. 2947-2955.

119. Vincent, A.L., et al., *Evaluation of hemagglutinin subtype 1 swine influenza viruses from the United States.* Veterinary microbiology, 2006. **118**(3): p. 212-222.

120. Pan, C., et al., *High genetic and antigenic similarity between a swine H3N2 influenza A virus and a prior human influenza vaccine virus: A possible immune pressure-driven cross-species transmission.* Biochemical and biophysical research communications, 2009. **385**(3): p. 402-407.

121. Zhu, H., et al., *Novel reassortment of Eurasian Avian-like and pandemic/2009 influenza viruses in swine: infectious potential to humans.* Journal of virology, 2011: p. JVI. 05352-11.

122. Luoh, S.-M., M. McGregor, and V. Hinshaw, *Hemagglutinin mutations related to antigenic variation in H1 swine influenza viruses.* Journal of virology, 1992. **66**(2): p. 1066-1073.

123. Karasin, A.I., S. Carman, and C.W. Olsen, *Identification of human H1N2 and human-swine reassortant H1N2 and H1N1 influenza A viruses among pigs in Ontario, Canada (2003 to 2005).* Journal of clinical microbiology, 2006. **44**(3): p. 1123-1126.

124. Yu, H., et al., *Genetic evolution of swine influenza A (H3N2) viruses in China from 1970 to 2006.* Journal of Clinical Microbiology, 2008. **46**(3): p. 1067-1075.

125. Tu, J., et al., *Isolation and molecular characterization of equine H3N8 influenza viruses from pigs in China.* Archives of virology, 2009. **154**(5): p. 887-890.

126. Lycett, S., et al., *Estimating reassortment rates in co-circulating Eurasian swine influenza viruses.* Journal of General Virology, 2012. **93**(11): p. 2326-2336.
