# Supplementary material for: Novel approach for identification of influenza virus host range and zoonotic transmissible sequences by determination of host-related associative positions in viral genome segments
Source: BMC Genomics. 2016 Nov 16;17:925. doi: 10.1186/s12864-016-3250-9 (PMC5112743; doi:10.1186/s12864-016-3250-9)
Supplement: Additional file 3: Table S1. — Listing the rules extracted from HA protein of influenza A in identification of host ranges. (DOCX 21 kb) [file 12864_2016_3250_MOESM3_ESM.docx]

**Table S1.** Rules extracted from HA protein of influenza A in identification of host ranges

| **Class** | **Rule** | **Support** | **Confidence** | **Algorithm** |
| --- | --- | --- | --- | --- |
| Avian | Att444 = D | 30.861% | 100% | CBA |
| Avian | Att540 = R and Att9 = - | 22.255% | 100% | CBA |
| Avian | Att540 = R and Att10 = M | 21.513% | 100% | CBA |
| Avian | Att117 = N and Att15 = L | 17.953% | 100% | CBA |
| Avian | Att517= A and Att121 = G and Att286=I and Att433 =D and Att451=E | 11.869% | 100% | DT |
| Avian | Att223 = T and Att8 = M | 9.941% | 100% | CBA |
| Avian | Att194 = T and Att8 = - | 9.941% | 100% | CBA |
| Avian | Att344 = S | 9.050% | 100% | CBA |
| Avian | Att530 = R and Att13 = I | 7.864% | 100% | CBA |
| Avian | Att389 = L and Att11 = I | 7.418% | 100% | CBA |
| Avian | Att503 = N and Att15 = F | 7.122% | 100% | CBA |
| Avian | Att210 = I | 6.677% | 100% | CBA |
| Avian | Att591 = A and Att15 = F | 5.490% | 100% | CBA |
| Avian | Att42 = S | 5.341% | 100% | CBA |
| Avian | Att270 = V | 5.341% | 100% | CBA |
| Avian | Att277 = I and Att8 = M | 3.709% | 100% | CBA |
| Avian | Att316 = D and Att9 = N | 3.709% | 100% | CBA |
| Avian | Att30 = V | 3.561% | 100% | CBA |
| Avian | Att107 = R | 3.561% | 100% | CBA |
| Avian | Att117 = N and Att8 = M | 3.561% | 100% | CBA |
| Avian | Att508 = R and Att13 = I | 3.412% | 100% | CBA |
| Avian | Att389 = L and Att15 = S | 3.412% | 100% | CBA |
| Avian | Att19 = M | 3.264% | 100% | CBA |
| Avian | Att389 = L and Att9 = K | 3.116% | 100% | CBA |
| Avian | Att242 = S | 2.967% | 100% | CBA |
| Avian | Att223 = D | 2.671% | 100% | CBA |
| Avian | Att224 = E and Att15 = V | 2.522% | 100% | CBA |
| Avian | Att317 = V and Att12 = K | 1.929% | 100% | CBA |
| Avian | Att129 = I and Att10 = E | 1.187% | 100% | CBA |
| Avian | Att331 = E | 1.187% | 100% | CBA |
| Avian | Att508 = R and Att8 = - | 1.187% | 100% | CBA |
| Avian | Att158 = S and Att14 = V | 11.128% | 98.684% | CBA |
| Avian | Att35 = K | 13.64 | 97.872% | Ripper |
| Human | Att153= T and Att265= N and Att140= S and Att405= V | 21.068% | 100% | DT |
| Human | Att176 = K and Att9 = - | 8.605% | 100% | CBA |
| Human | Att194 = K and Att9 = K | 6.973% | 100% | CBA |
| Human | Att571 = I and Att13 = I | 5.341% | 100% | CBA |
| Human | Att173 = L and Att13 = I | 4.303% | 100% | CBA |
| Human | Att220 = V | 3.116% | 100% | CBA |
| Human | Att260 = V | 2.967% | 100% | CBA |
| Human | Att71 = V | 2.522% | 100% | CBA |
| Human | Att222 = N and Att8 = - | 2.522% | 100% | CBA |
| Human | Att434 = K and Att11 = A | 2.522% | 100% | CBA |
| Human | Att213 = M and Att8 = - | 2.374% | 100% | CBA |
| Human | Att19 = T and Att10 = M | 2.374% | 100% | CBA |
| Human | Att33 = M | 2.226% | 100% | CBA |
| Human | Att151 = A and Att9 = N | 2.226% | 100% | CBA |
| Human | Att510 = K and Att9 = K | 2.077% | 100% | CBA |
| Human | Att359 = K and Att12 = A | 2.077% | 100% | CBA |
| Human | Att68 = R and Att12 = K | 2.077% | 100% | CBA |
| Human | Att223 = A and Att14 = I | 2.077% | 100% | CBA |
| Human | Att223 = N and Att8 = M | 1.929% | 100% | CBA |
| Human | Att448 = I and Att10 = K | 1.929% | 100% | CBA |
| Human | Att317 = V and Att10 = E | 1.929% | 100% | CBA |
| Human | Att273 = S and Att8 = - | 1.780% | 100% | CBA |
| Human | Att168 = S and Att9 = K | 1.780% | 100% | CBA |
| Human | Att107 = N and Att12 = I | 1.780% | 100% | CBA |
| Human | Att163 = - and Att9 = M | 1.632% | 100% | CBA |
| Human | Att374 = G | 1.484% | 100% | CBA |
| Human | Att358 = S and Att8 = - | 1.335% | 100% | CBA |
| Human | Att207 = E and Att11 = V | 1.335% | 100% | CBA |
| Human | Att260 = X | 1.187% | 100% | CBA |
| Human | Att259 = E and Att10 = K | 1.187% | 100% | CBA |
| Human | Att16 = M and Att8 = - | 1.039% | 100% | CBA |
| Human | Att222 = E and Att10 = M | 1.039% | 100% | CBA |
| Swine | Att177 = N and Att9 = M | 12.908% | 100% | CBA |
| Swine | Att163 = R and Att9 = M | 11.424% | 100% | CBA |
| Swine | Att223 = T and Att11 = A | 6.825% | 100% | CBA |
| Swine | Att448 = I | 5.638% | 100% | CBA |
| Swine | Att52 = I and Att9 = M | 5.045% | 100% | CBA |
| Swine | Att234 = T and Att11 = A | 4.599% | 100% | CBA |
| Swine | Att331 = H and Att11 = A | 4.451% | 100% | CBA |
| Swine | Att324 = T and Att13 = L | 4.154% | 100% | CBA |
| Swine | Att222 = N and Att8 = - | 2.522% | 100% | CBA |
| Swine | Att455 = K and Att14 = L and Att235 = K | 2.373% | 100% | Ripper |
| Swine | Att260 = X | 1.187% | 100% | CBA |
| Swine | Att240 = S and Att11 = A | 24.036% | 98.182% | CBA |
|  | **Iteration 2** |  |  |  |
| Human | Att223 = T and Att16 = I | 10.569% | 100% | CBA |
| Human | Att227 = S and Att9 = K | 5.691% | 100% | CBA |
| Human | Att167 = N and Att13 = A | 4.878% | 100% | CBA |
| Human | Att386 = T and Att17 = L | 4.878% | 100% | CBA |
| Human | Att586 = V and Att227= S and Att510 = K | 4.065% | 100% | DT |
| Human | Att155 = T and Att260 = L | 6.504% | 100% | Ripper |
| Human | Att33 = G | 13.008% | 94.117% | Ripper |
| Human | Att95 = I | 8.943% | 91.666% | DT |
| Human | Att326 = K and Att127 = D | 12.195% | 83.333% | Ripper |
| Swine | Att226 = K | 4.049% | 100% | CBA |
| Swine | Att563 = I and Att13 = I | 3.239% | 100% | CBA |
| Swine | Att377 = S | 2.429% | 100% | CBA |
| Swine | Att313 = R and Att15 = I | 2.429% | 100% | CBA |
| Swine | Att19 = V and Att8 = - | 2.024% | 100% | CBA |
