# Supplementary material for: Novel approach for identification of influenza virus host range and zoonotic transmissible sequences by determination of host-related associative positions in viral genome segments
Source: BMC Genomics. 2016 Nov 16;17:925. doi: 10.1186/s12864-016-3250-9 (PMC5112743; doi:10.1186/s12864-016-3250-9)
Supplement: Additional file 4: Table S2. — Listing the rules extracted from M1 protein of influenza A in identification of host ranges. (DOCX 17 kb) [file 12864_2016_3250_MOESM4_ESM.docx]

**Table S2.** Rules extracted from M1 protein of influenza A in identification of host ranges

| **Class** | **Rule** | **Support** | **Confidence** | **Algorithm** |
| --- | --- | --- | --- | --- |
| Avian | Att232 = N | 17.885% | 100% | CBA |
| Avian | Att230 = R and Att115 = V | 17.500% | 100% | CBA |
| Avian | Att207 = N and Att142 = V | 17.308% | 100% | CBA |
| Avian | Att168 = I | 17.115% | 100% | CBA |
| Avian | Att224 = N and Att95 = R | 16.346% | 100% | CBA |
| Avian | Att59 = M | 6.731% | 100% | CBA |
| Avian | Att157 = S and Att144 = L | 4.231% | 100% | CBA |
| Avian | Att31 = I | 1.923% | 100% | CBA |
| Avian | Att234 = I and Att157 = S | 1.538% | 100% | CBA |
| Avian | Att239 = T and Att115 = V | 1.154% | 100% | CBA |
| Avian | Att214= Q and Att121 = T and Att143 = A and Att157 = S | 40.384 | 99.526% | DT |
| Avian | Att224 = N | 20.384% | 98.148% | Ripper |
| Human | Att224 = N and Att144 = L | 5.385% | 100% | CBA |
| Human | Att205 = I and Att15 = I | 4.808% | 100% | CBA |
| Human | Att218 = A and Att167 = T | 4.423% | 100% | CBA |
| Human | Att227 = T and Att101 = K | 3.462% | 100% | CBA |
| Human | Att140 = A and Att101 = K | 3.269% | 100% | CBA |
| Human | Att137 = A and Att15 = I | 2.692% | 100% | CBA |
| Human | Att214 = Q and Att116 = S | 2.692% | 100% | CBA |
| Human | Att31 = I | 1.923% | 100% | CBA |
| Human | Att219 = I and Att137 = A | 11.923% | 98.413% | CBA |
| Human | Att207 = N and Att116 = S | 20.769% | 98.182% | CBA |
| Human | Att157 = A | 5.577% | 93.548% | CBA |
| Swine | Att168 = T and Att116 = A and Att95 = K and Att227 = A | 7.692% | 100% | DT |
| Swine | Att214 = H and Att207 = S | 5.962% | 100% | CBA |
| Swine | Att30 = G | 5.769% | 100% | CBA |
| Swine | Att248 = I and Att116 = S | 5.769% | 100% | CBA |
| Swine | Att181 = I and Att139 = T | 2.885% | 100% | CBA |
| Swine | Att31 = I | 1.923% | 100% | CBA |
| Swine | Att252 = R | 1.538% | 100% | CBA |
| Swine | Att140 = A and Att15 = V | 1.346% | 100% | CBA |
| Swine | Att95 = K and Att230 = K and Att46 = L | 12.307% | 96.969% | Ripper |
