# Supplementary material for: Novel approach for identification of influenza virus host range and zoonotic transmissible sequences by determination of host-related associative positions in viral genome segments
Source: BMC Genomics. 2016 Nov 16;17:925. doi: 10.1186/s12864-016-3250-9 (PMC5112743; doi:10.1186/s12864-016-3250-9)
Supplement: Additional file 5: Table S3. — Listing the rules extracted from M2 protein of influenza A in identification of host ranges. (DOCX 18 kb) [file 12864_2016_3250_MOESM5_ESM.docx]

**Table S3.** Rules extracted from M2 protein of influenza A in identification of host ranges

| **Class** | **Rule** | **Support** | **Confidence** | **Algorithm** |
| --- | --- | --- | --- | --- |
| Avian | Att66 = A | 9.800% | 100% | CBA |
| Avian | Att89 = S and Att16 = E | 3.118% | 100% | CBA |
| Avian | Att77 = R and Att27 = A | 2.227% | 100% | CBA |
| Avian | Att55 = I and Att31 = S | 1.559% | 100% | CBA |
| Avian | Att44 = N | 1.336% | 100% | CBA |
| Avian | Att54 = C | 1.336% | 100% | CBA |
| Avian | Att18 = K and Att10 = L | 1.336% | 100% | CBA |
| Avian | Att21 = G and Att10 = L | 1.114% | 100% | CBA |
| Avian | Att27 = I and Att13 = S | 1.114% | 100% | CBA |
| Avian | Att77 = R and Att20 = S and Att69 = P and Att61 = R and Att16 = E | 41.202% | 98.930% | DT |
| Human | Att66 = A | 9.800% | 100% | CBA |
| Human | Att82 = S and Att78 = K | 6.236% | 100% | CBA |
| Human | Att13 = T | 3.341% | 100% | CBA |
| Human | Att14 = G and Att68 = M | 3.340% | 100% | DT |
| Human | Att18 = R and Att10 = L | 3.118% | 100% | CBA |
| Human | Att14 = E and Att27 = A | 2.673% | 100% | CBA |
| Human | Att14 = E and Att50 = F | 2.673% | 100% | CBA |
| Human | Att24 = E | 2.227% | 100% | CBA |
| Human | Att51 = V | 2.227% | 100% | CBA |
| Human | Att14 = G and Att95 = V | 1.336% | 100% | CBA |
| Human | Att65 = M | 1.114% | 100% | CBA |
| Human | Att55 = F and Att77 = Q | 23.608% | 98.148% | CBA |
| Human | Att28 = V and Att11 = I | 11.581% | 94.545% | CBA |
| Swine | Att77 = R and Att79 = K | 8.018% | 100% | CBA |
| Swine | Att55 = L and Att77 = Q | 5.791% | 100% | CBA |
| Swine | Att14 = E and Att27 = I | 2.673% | 100% | CBA |
| Swine | Att31 = N and Att10 = L | 2.004% | 100% | CBA |
| Swine | Att23 = G | 1.782% | 100% | CBA |
| Swine | Att97 = E and Att18 = R and Att12 = K | 1.782% | 100% | CBA |
| Swine | Att28 = I and Att10 = L | 1.336% | 100% | CBA |
| Swine | Att28 = I and Att50 = F | 1.336% | 100% | CBA |
| Swine | Att18 = R and Att27 = I | 1.336% | 100% | CBA |
| Swine | Att65 = M | 1.114% | 100% | CBA |
| Swine | Att54 = R and Att20 = N | 8.463% | 95.000% | CBA |
| Swine | Att60 = K and Att77 = Q | 25.612% | 86.466% | CBA |
