# Supplementary material for: Novel approach for identification of influenza virus host range and zoonotic transmissible sequences by determination of host-related associative positions in viral genome segments
Source: BMC Genomics. 2016 Nov 16;17:925. doi: 10.1186/s12864-016-3250-9 (PMC5112743; doi:10.1186/s12864-016-3250-9)
Supplement: Additional file 6: Table S4. — Listing the rules extracted from NA protein of influenza A in identification of host ranges. (DOCX 22 kb) [file 12864_2016_3250_MOESM6_ESM.docx]

**Table S4.** Rules extracted from NA protein of influenza A in identification of host ranges

| **Class** | **Rule** | **Support** | **Confidence** | **Algorithm** |
| --- | --- | --- | --- | --- |
| Avian | Att178 = A and Att415 =V and Att451 = R | 25.724% | 100% | Ripper |
| Avian | Att274 = K and Att22 = V | 21.295% | 100% | CBA |
| Avian | Att339 = N and Att22 = V | 20.954% | 100% | CBA |
| Avian | Att85 = L | 19.080% | 100% | CBA |
| Avian | Att456 = S and Att9 = P | 18.228% | 100% | CBA |
| Avian | Att111 = V | 18.058% | 100% | CBA |
| Avian | Att386 = N and Att9 = P | 15.673% | 100% | CBA |
| Avian | Att364 = Y and Att23 = I | 14.480% | 100% | CBA |
| Avian | Att282 = D and Att9 = P | 14.140% | 100% | CBA |
| Avian | Att74 = - and Att23 = I | 13.458% | 100% | CBA |
| Avian | Att23 = L | 10.733% | 100% | CBA |
| Avian | Att289 = I and Att304 = V | 8.688% | 100% | Ripper |
| Avian | Att61 = V | 8.518% | 100% | CBA |
| Avian | Att464 = N and Att17 = S | 8.348% | 100% | CBA |
| Avian | Att407 = S and Att304 = V | 7.155% | 100% | CBA |
| Avian | Att79 = I | 6.474% | 100% | CBA |
| Avian | Att322 = A | 6.303% | 100% | CBA |
| Avian | Att49 = N | 6.149 | 100% | Ripper |
| Avian | Att83 = K | 5.281% | 100% | CBA |
| Avian | Att97 = K | 3.748% | 100% | CBA |
| Avian | Att16 = L | 3.066% | 100% | CBA |
| Avian | Att53 = G and Att20 = C | 2.726% | 100% | CBA |
| Avian | Att410 = L and Att38 = I | 1.873% | 100% | Ripper |
| Avian | Att212 = S | 1.193% | 100% | CBA |
| Avian | Att50 = S and Att19 = V | 1.193% | 100% | CBA |
| Human | Att300 = G and Att207 = I | 8.006% | 100% | Ripper |
| Human | Att280 = T | 5.451% | 100% | CBA |
| Human | Att23 = T | 4.940% | 100% | CBA |
| Human | Att356 = M and Att19 = I | 4.600% | 100% | CBA |
| Human | Att24 = G and Att21 = I | 4.259% | 100% | CBA |
| Human | Att50 = Y | 3.407% | 100% | CBA |
| Human | Att323 = N and Att20 = S | 3.066% | 100% | CBA |
| Human | Att73 = T | 2.896% | 100% | CBA |
| Human | Att234 = E | 2.896% | 100% | CBA |
| Human | Att32 = V and Att19 = I | 2.896% | 100% | CBA |
| Human | Att260 = S and Att19 = I | 2.896% | 100% | CBA |
| Human | Att51 = K | 2.726% | 100% | CBA |
| Human | Att411 = R and Att19 = I | 2.726% | 100% | CBA |
| Human | Att11 = K | 2.555% | 100% | CBA |
| Human | Att288 = Y | 2.555% | 100% | CBA |
| Human | Att95 = N and Att13 = I | 2.215% | 100% | CBA |
| Human | Att38 = V | 1.874% | 100% | CBA |
| Human | Att213 = E | 1.874% | 100% | CBA |
| Human | Att475 = G and Att16 = I | 1.874% | 100% | CBA |
| Human | Att371 = Y and Att18 = S | 1.874% | 100% | CBA |
| Human | Att91 = A and Att19 = V | 1.874% | 100% | CBA |
| Human | Att274 = R and Att21 = M | 1.874% | 100% | CBA |
| Human | Att96 = I and Att9 = P | 1.704% | 100% | CBA |
| Human | Att201 = A and Att17 = S | 1.704% | 100% | CBA |
| Human | Att76 = T | 1.363% | 100% | CBA |
| Human | Att107 = G | 1.363% | 100% | CBA |
| Human | Att201 = A and Att16 = L | 1.363% | 100% | CBA |
| Human | Att84 = V and Att19 = I | 1.363% | 100% | CBA |
| Human | Att478 = D and Att19 = I | 1.363% | 100% | CBA |
| Human | Att138 = H and Att20 = S | 1.363% | 100% | CBA |
| Human | Att389 = K | 1.193% | 100% | CBA |
| Human | Att62 = T and Att20 = S | 1.193% | 100% | CBA |
| Human | Att410 = T | 1.022% | 100% | CBA |
| Human | Att454 = R and Att14 = I | 1.022% | 100% | CBA |
| Human | Att25 = M | 25.894% | 98.701% | CBA |
| Swine | Att61 = I and Att260 = N and Att364 =N | 19.0.8% | 100% | Ripper |
| Swine | Att85 = V and Att20 = C | 6.985% | 100% | CBA |
| Swine | Att301 = V and Att20 = C | 6.133% | 100% | CBA |
| Swine | Att394 = I and Att21 = L | 5.622% | 100% | CBA |
| Swine | Att454 = Q and Att486 = D and Att387= S | 3.577% | 100% | Ripper |
| Swine | Att279 = V and Att21 = L | 2.555% | 100% | CBA |
| Swine | Att371 = Y and Att20 = C | 1.533% | 100% | CBA |
| Swine | Att107 = G | 1.363% | 100% | CBA |
| Swine | Att246 = I and Att44 = R | 1.363% | 100% | Ripper |
| Swine | Att61 = I and Att106 = I | 12.265% | 90% | Ripper |
|  | **Iteration 2** |  |  |  |
| Human | Att352 = R and Att28 = F | 13.816% | 100% | CBA |
| Human | Att53 = S and Att350 = Y | 11.184% | 100% | CBA |
| Human | Att52 = S and Att19 = V | 9.868% | 100% | CBA |
| Human | Att361 = S | 7.237% | 100% | CBA |
| Human | Att53 = G and Att22 = T | 6.579% | 100% | CBA |
| Human | Att112 = H and Att23 = I | 6.579% | 100% | CBA |
| Human | Att266 = R and Att28 = F | 4.605% | 100% | CBA |
| Human | Att50 = I | 2.632% | 100% | CBA |
| Human | Att387 = L and Att26 = I | 15.132% | 95.833% | CBA |
| Human | Att478 = G and Att26 = V | 6.579% | 90.909% | CBA |
| Swine | Att155 = R and Att52 = N and Att30 = M | 3.448% | 90.000% | Ripper |
| Swine | Att326 = T | 1.532% | 100% | Ripper |
