# Supplementary material for: Novel approach for identification of influenza virus host range and zoonotic transmissible sequences by determination of host-related associative positions in viral genome segments
Source: BMC Genomics. 2016 Nov 16;17:925. doi: 10.1186/s12864-016-3250-9 (PMC5112743; doi:10.1186/s12864-016-3250-9)
Supplement: Additional file 7: Table S5. — Listing the rules extracted from NP protein of influenza A in identification of host ranges. (DOCX 20 kb) [file 12864_2016_3250_MOESM7_ESM.docx]

**Table S5.** Rules extracted from NP protein of influenza A in identification of host ranges

| **Class** | **Rule** | **Support** | **Confidence** | **Algorithm** |
| --- | --- | --- | --- | --- |
| Avian | Att383 = S and Att142 = L | 23.166% | 100% | CBA |
| Avian | Att412 = I and Att40 = S | 17.181% | 100% | CBA |
| Avian | Att357 = R and Att456 = N | 12.355% | 100% | CBA |
| Avian | Att111 = M and Att357 = R | 11.776% | 100% | CBA |
| Avian | Att390 = R and Att414 = I | 6.950% | 100% | CBA |
| Avian | Att276 = I | 3.089% | 100% | CBA |
| Avian | Att356 = A | 1.931% | 100% | CBA |
| Avian | Att379 = T and Att200 = V | 1.931% | 100% | CBA |
| Avian | Att349 = V and Att478 = A | 1.931% | 100% | CBA |
| Avian | Att58 = Y and Att478 = A | 1.931% | 100% | DT |
| Avian | Att200 = I and Att216 = D | 1.544% | 100% | CBA |
| Avian | Att504 = S and Att412 = V | 1.544% | 100% | CBA |
| Avian | Att242 = K and Att107 = N | 1.351% | 100% | CBA |
| Avian | Att457 = S | 1.158% | 100% | CBA |
| Avian | Att105 = R and Att142 = L | 42.857% | 99.552% | CBA |
| Avian | Att295 = Y and Att383 = N | 21.622% | 99.115% | CBA |
| Avian | Att429 = A and Att83 = R | 22.780% | 98.333% | CBA |
| Avian | Att450 = I and Att383 = N | 20.077% | 98.113% | CBA |
| Avian | Att195 = M and Att383 = N | 19.884% | 98.095% | CBA |
| Human | Att106 = V and Att59 = D | 6.757% | 100% | CBA |
| Human | Att223 = V and Att106 = V | 6.757% | 100% | CBA |
| Human | Att431 = I and Att37 = K | 6.371% | 100% | CBA |
| Human | Att359 = L | 4.633% | 100% | CBA |
| Human | Att412 = V | 4.440% | 100% | CBA |
| Human | Att390 = G and Att111 = M | 4.440% | 100% | CBA |
| Human | Att107 = N and Att44 = R | 2.703% | 100% | CBA |
| Human | Att432 = L | 2.317% | 100% | CBA |
| Human | Att295 = Y and Att27 = D | 1.931% | 100% | CBA |
| Human | Att351 = G and Att115 = I | 1.544% | 100% | CBA |
| Human | Att58 = H and Att39 = I | 1.351% | 100% | CBA |
| Human | Att410 = G and Att69 = V | 1.351% | 100% | CBA |
| Human | Att363 = R and Att22 = G | 1.158% | 100% | CBA |
| Human | Att363 = K and Att37 = K | 10.231% | 98.148% | Ripper |
| Human | Att59 = D | 17.568% | 97.849% | CBA |
| Human | Att58 = Q | 4.247% | 95.652% | Ripper |
| Swine | Att383 = S and Att125 = V | 7.336% | 100% | CBA |
| Swine | Att504 = N and Att195 = I | 7.336% | 100% | CBA |
| Swine | Att504 = N and Att27 = D | 7.336% | 100% | CBA |
| Swine | Att106 = V and Att59 = D | 6.757% | 100% | CBA |
| Swine | Att377 = M and Att390 = K | 5.985% | 100% | CBA |
| Swine | Att504 = N and Att436 = S | 5.405% | 100% | CBA |
| Swine | Att329 = V | 4.826% | 100% | CBA |
| Swine | Att195 = M and Att105 = K | 4.440% | 100% | DT |
| Swine | Att349 = I | 3.861% | 100% | CBA |
| Swine | Att44 = R and Att479 = S | 3.861% | 100% | CBA |
| Swine | Att378 = E and Att429 = T | 3.475% | 100% | CBA |
| Swine | Att502 = F | 2.317% | 100% | CBA |
| Swine | Att431 = V and Att363 = R | 2.124% | 100% | CBA |
| Swine | Att40 = G and Att412 = V | 1.931% | 100% | CBA |
| Swine | Att83 = R and Att462 = M | 1.737% | 100% | CBA |
| Swine | Att479 = S and Att107 = N | 1.351% | 100% | CBA |
| Swine | Att479 = S and Att478 = A | 1.351% | 100% | CBA |
| Swine | Att498 = S | 1.158% | 100% | CBA |
| Swine | Att203 = I and Att24 = D | 1.158% | 100% | CBA |
|  | **Iteration2** |  |  |  |
| Human | Att488= N and Att276=V and Att120= E and Att456= S and Att458= R  and Att358 = V | 12.741% | 100% | DT |
| Swine | Att122 = V | 2.336% | 100% | CBA |
| Swine | Att295 = Y and Att27 = D | 4.672% | 100% | CBA |
| Swine | Att324 = S | 1.869% | 100% | CBA |
| Swine | Att192 = I and Att58 = H | 1.869% | 100% | CBA |
