# Supplementary material for: Novel approach for identification of influenza virus host range and zoonotic transmissible sequences by determination of host-related associative positions in viral genome segments
Source: BMC Genomics. 2016 Nov 16;17:925. doi: 10.1186/s12864-016-3250-9 (PMC5112743; doi:10.1186/s12864-016-3250-9)
Supplement: Additional file 8: Table S6. — Listing the rules extracted from NS1 protein of influenza A in identification of host ranges. (DOCX 20 kb) [file 12864_2016_3250_MOESM8_ESM.docx]

**Table S6.** Rules extracted from NS1 protein of influenza A in identification of host ranges

| **Class** | **Rule** | **Support** | **Confidence** | **Algorithm** |
| --- | --- | --- | --- | --- |
| Avian | Att118 = K and Att53 = D | 22.351% | 100% | CBA |
| Avian | Att200 = I and Att74 = D | 21.773% | 100% | CBA |
| Avian | Att200 = I and Att91 = T | 21.580% | 100% | CBA |
| Avian | Att187 = L and Att129 = T | 15.414% | 100% | CBA |
| Avian | Att229 = E and Att48 = N | 14.451% | 100% | CBA |
| Avian | Att77 = L and Att27 = M | 7.129% | 100% | CBA |
| Avian | Att214 = P and Att27 = M | 7.129% | 100% | CBA |
| Avian | Att199 = I and Att18 = V | 6.744% | 100% | CBA |
| Avian | Att199 = N and Att55 = R | 5.395% | 100% | CBA |
| Avian | Att199 = N and Att127 = D | 5.202% | 100% | CBA |
| Avian | Att228 = V and Att81 = I | 5.010% | 100% | CBA |
| Avian | Att145 = T and Att87 = P | 5.010% | 100% | CBA |
| Avian | Att147 = V and Att127 = D | 5.010% | 100% | CBA |
| Avian | Att187 = L and Att98 = I | 4.817% | 100% | CBA |
| Avian | Att155 = D and Att53 = D | 4.432% | 100% | CBA |
| Avian | Att207 = G | 4.046% | 100% | CBA |
| Avian | Att112 = T and Att48 = N | 3.661% | 100% | CBA |
| Avian | Att93 = I | 2.890% | 100% | CBA |
| Avian | Att139 = T and Att14 = F | 2.890% | 100% | CBA |
| Avian | Att114 = P and Att106 = I | 2.119% | 100% | CBA |
| Avian | Att141 = N and Att112 = A | 2.119% | 100% | CBA |
| Avian | Att63 = L and Att24 = D | 1.927% | 100% | CBA |
| Avian | Att196 = I and Att56 = T | 1.927% | 100% | CBA |
| Avian | Att231 = E and Att56 = A | 1.734% | 100% | CBA |
| Avian | Att230 = S and Att86 = T | 1.734% | 100% | CBA |
| Avian | Att232 = V and Att86 = T | 1.734% | 100% | CBA |
| Avian | Att114 = P and Att103 = L | 1.541% | 100% | CBA |
| Avian | Att208 = C and Att74 = D | 1.349% | 100% | CBA |
| Avian | Att211 = G | 1.156% | 100% | CBA |
| Avian | Att59 = C and Att4 = N | 1.156% | 100% | CBA |
| Avian | Att111 = M and Att25 = Q | 1.156% | 100% | CBA |
| Avian | Att112 = A and Att26 = E | 30.829% | 99.379% | CBA |
| Avian | Att27 = M | 9.249% | 97.959% | CBA |
| Avian | Att211 = D and Att25 = Q | 35.645% | 97.368% | CBA |
| Avian | Att228 = V and Att130 = I | 7.129% | 97.368% | CBA |
| Human | Att173 = G and Att48 = N | 8.863% | 100% | CBA |
| Human | Att114 = P and Att59 = R | 7.707% | 100% | CBA |
| Human | Att125 = I and Att78 = R | 6.358% | 100% | CBA |
| Human | Att213 = R and Att56 = A | 5.973% | 100% | CBA |
| Human | Att233 = R and Att78 = K | 5.202% | 100% | CBA |
| Human | Att214 = S and Att87 = P | 5.202% | 100% | CBA |
| Human | Att84 = A and Att27 = L | 4.817% | 100% | CBA |
| Human | Att114 = P and Att53 = N | 4.432% | 100% | CBA |
| Human | Att206 = G and Att67 = R | 4.239% | 100% | CBA |
| Human | Att155 = G and Att77 = L | 3.854% | 100% | CBA |
| Human | Att224 = M and Att103 = L | 3.661% | 100% | CBA |
| Human | Att173 = E | 3.276% | 100% | CBA |
| Human | Att95 = L and Att90 = I | 3.083% | 100% | CBA |
| Human | Att93 = I | 2.890% | 100% | CBA |
| Human | Att227 = - and Att27 = M | 2.890% | 100% | CBA |
| Human | Att174 = K and Att77 = F | 2.890% | 100% | CBA |
| Human | Att216 = F and Att81 = - | 2.890% | 100% | CBA |
| Human | Att111 = I and Att18 = V | 2.505% | 100% | CBA |
| Human | Att91 = A and Att87 = P | 1.927% | 100% | CBA |
| Human | Att79 = K | 1.734% | 100% | CBA |
| Human | Att139 = V and Att81 = I | 1.734% | 100% | CBA |
| Human | Att85 = L | 1.541% | 100% | CBA |
| Human | Att117 = V and Att112 = A | 1.541% | 100% | CBA |
| Human | Att218 = S and Att18 = I | 1.156% | 100% | CBA |
| Human | Att131 = V and Att5 = T | 16.570% | 98.851% | CBA |
| Human | Att211 = N and Att112 = I | 15.992% | 97.647% | CBA |
| Swine | Att114 = S and Att26 = G | 12.331% | 100% | CBA |
| Swine | Att48 = S and Att26 = G | 11.175% | 100% | CBA |
| Swine | Att127 = E and Att114 = S | 8.285% | 100% | CBA |
| Swine | Att219 = E and Att114 = S | 8.285% | 100% | CBA |
| Swine | Att180 = V and Att48 = S | 7.900% | 100% | CBA |
| Swine | Att207 = S and Att44 = K | 7.707% | 100% | CBA |
| Swine | Att131 = V and Att125 = I | 7.322% | 100% | CBA |
| Swine | Att114 = S and Att60 = V | 6.166% | 100% | CBA |
| Swine | Att207 = S and Att76 = T | 5.973% | 100% | CBA |
| Swine | Att199 = A and Att84 = V | 5.202% | 100% | CBA |
| Swine | Att230 = P and Att103 = F | 5.202% | 100% | CBA |
| Swine | Att216 = F and Att131 = T | 4.817% | 100% | CBA |
| Swine | Att223 = E | 4.239% | 100% | CBA |
| Swine | Att228 = T and Att10 = Q | 3.468% | 100% | CBA |
| Swine | Att93 = I | 2.890% | 100% | CBA |
| Swine | Att145 = N and Att44 = K | 2.505% | 100% | CBA |
| Swine | Att77 = F and Att27 = L | 1.734% | 100% | CBA |
| Swine | Att67 = K and Att41 = K | 1.541% | 100% | CBA |
| Swine | Att194 = I and Att7 = S | 1.349% | 100% | CBA |
| Swine | Att182 = V and Att77 = F | 1.349% | 100% | CBA |
