# Supplementary material for: Novel approach for identification of influenza virus host range and zoonotic transmissible sequences by determination of host-related associative positions in viral genome segments
Source: BMC Genomics. 2016 Nov 16;17:925. doi: 10.1186/s12864-016-3250-9 (PMC5112743; doi:10.1186/s12864-016-3250-9)
Supplement: Additional file 9: Table S7. — Listing the rules extracted from NS2 protein of influenza A in identification of host ranges. (DOCX 20 kb) [file 12864_2016_3250_MOESM9_ESM.docx]

**Table S7.** Rules extracted from NS2 protein of influenza A in identification of host ranges

| **Class** | **Rule** | **Support** | **Confidence** | **Algorithm** |
| --- | --- | --- | --- | --- |
| Avian | Att60 = I | 16.486% | 100% | CBA |
| Avian | Att22 = A | 15.835% | 100% | CBA |
| Avian | Att55 = F and Att49 = V | 10.629% | 100% | Ripper |
| Avian | Att32 = I and Att14 = V | 9.978% | 100% | CBA |
| Avian | Att115 = A and Att6 = V | 7.809% | 100% | CBA |
| Avian | Att57 = S and Att22 = E | 6.508% | 100% | CBA |
| Avian | Att57 = S and Att40 = I | 6.074% | 100% | CBA |
| Avian | Att89 = V and Att3 = S | 3.254% | 100% | CBA |
| Avian | Att44 = L and Att32 = I | 1.952% | 100% | CBA |
| Avian | Att107 = L and Att89 = I and Att3 = P | 1.952% | 100% | CBA |
| Avian | Att52 = I | 1.302% | 100% | CBA |
| Avian | Att52 = V and Att6 = V | 1.302% | 100% | CBA |
| Avian | Att47 = K and Att40 = L | 1.302% | 100% | CBA |
| Avian | Att83 = M and Att6 = V | 1.085% | 100% | CBA |
| Avian | Att92 = S and Att32 = I | 1.085% | 100% | CBA |
| Avian | Att89 = T and Att60 = S | 1.085% | 100% | CBA |
| Avian | Att88 = K and Att70 = S | 37.310% | 96.089% | CBA |
| Human | Att60 = I and Att14 = V | 8.677% | 100% | CBA |
| Human | Att57 = S and Att14 = L | 4.555% | 100% | CBA |
| Human | Att107 = F and Att57 = S | 4.338% | 100% | CBA |
| Human | Att14 = K | 3.471% | 100% | CBA |
| Human | Att22 = A and Att7 = L | 3.471% | 100% | CBA |
| Human | Att48 = T and Att14 = T | 3.037% | 100% | CBA |
| Human | Att86 = K and Att32 = I | 2.820% | 100% | CBA |
| Human | Att40 = L and Att22 = E | 2.603% | 100% | CBA |
| Human | Att89 = I and Att22 = E | 1.952% | 100% | CBA |
| Human | Att60 = I and Att52 = T | 1.735% | 100% | CBA |
| Human | Att89 = E | 1.085% | 100% | CBA |
| Human | Att40 = V and Att6 = V | 1.085% | 100% | CBA |
| Human | Att67 = D and Att60 = S | 1.085% | 100% | CBA |
| Human | Att55 = F and Att49 = V | 10.412% | 97.959% | Ripper |
| Human | Att115 = A and Att36 = E | 25.162% | 97.478% | Ripper |
| Human | Att83 = M and Att6 = M | 18.438% | 97.701% | CBA |
| Swine | Att63 = E and Att48 = A | 8.677% | 100% | CBA |
| Swine | Att107 = L and Att86 = K | 8.243% | 100% | CBA |
| Swine | Att86 = K and Att48 = A | 8.026% | 100% | CBA |
| Swine | Att27 = G | 6.725% | 100% | CBA |
| Swine | Att57 = Y and Att48 = A | 6.725% | 100% | CBA |
| Swine | Att70 = G and Att52 = T | 4.772% | 100% | CBA |
| Swine | Att70 = G and Att49 = L | 4.121% | 100% | Ripper |
| Swine | Att32 = I and Att26 = K | 2.3865 | 100% | DT |
| Swine | Att113 = M | 2.169% | 100% | CBA |
| Swine | Att29 = S | 1.735% | 100% | CBA |
| Swine | Att60 = N and Att48 = A and Att3 = P | 1.735% | 100% | CBA |
| Swine | Att107 = L and Att89 = T | 1.302% | 100% | CBA |
| Swine | Att89 = E | 1.085% | 100% | CBA |
| Swine | Att63 = E and Att14 = M | 27.332% | 90.647% | CBA |
