# Supplementary material for: Novel approach for identification of influenza virus host range and zoonotic transmissible sequences by determination of host-related associative positions in viral genome segments
Source: BMC Genomics. 2016 Nov 16;17:925. doi: 10.1186/s12864-016-3250-9 (PMC5112743; doi:10.1186/s12864-016-3250-9)
Supplement: Additional file 10: Table S8. — Listing the rules extracted from PA protein of influenza A in identification of host ranges. (DOCX 21 kb) [file 12864_2016_3250_MOESM10_ESM.docx]

**Table S8.** Rules extracted from PA protein of influenza A in identification of host ranges

| **Class** | **Rule** | **Support** | **Confidence** | **Algorithm** |
| --- | --- | --- | --- | --- |
| Avian | Att129 = T | 18.486% | 100% | CBA |
| Avian | Att400 = Q | 4.009% | 100% | CBA |
| Avian | Att101 = D and Att20 = A | 4.009% | 100% | CBA |
| Avian | Att272 = E | 3.563% | 100% | CBA |
| Avian | Att322 = V and Att66 = G | 3.118% | 100% | CBA |
| Avian | Att545 = V | 2.895% | 100% | CBA |
| Avian | Att70 = A and Att37 = S | 2.673% | 100% | CBA |
| Avian | Att409 = N and Att37 = S | 2.673% | 100% | DT |
| Avian | Att348 = L and Att55 = D | 2.673% | 100% | CBA |
| Avian | Att140 = T | 2.227% | 100% | CBA |
| Avian | Att85 = T and Att20 = T | 2.227% | 100% | CBA |
| Avian | Att201 = V | 1.559% | 100% | CBA |
| Avian | Att261 = M | 1.559% | 100% | CBA |
| Avian | Att387 = V and Att28 = L | 1.559% | 100% | CBA |
| Avian | Att210 = A | 1.336% | 100% | CBA |
| Avian | Att535 = Y | 1.336% | 100% | CBA |
| Avian | Att682 = N | 1.336% | 100% | CBA |
| Avian | Att423 = M and Att14 = V | 1.336% | 100% | CBA |
| Avian | Att208 = A | 1.114% | 100% | CBA |
| Avian | Att237 = K and Att20 = A | 1.114% | 100% | CBA |
| Avian | Att99 = R and Att44 = V | 1.114% | 100% | CBA |
| Avian | Att409 = S and Att216 = D | 43.207% | 97.979% | Ripper |
| Human | Att337 = A and Att58 = S | 10.468% | 100% | CBA |
| Human | Att269 = R and Att57 = Q | 5.791% | 100% | CBA |
| Human | Att437 = H and Att28 = L | 4.454% | 100% | CBA |
| Human | Att332 = P and Att28 = L | 4.232% | 100% | CBA |
| Human | Att256 = Q and Att57 = Q | 3.786% | 100% | CBA |
| Human | Att388 = D | 3.563% | 100% | CBA |
| Human | Att337 = T and Att63 = I | 3.341% | 100% | CBA |
| Human | Att224 = P | 3.118% | 100% | CBA |
| Human | Att272 = N and Att65 = S | 3.118% | 100% | CBA |
| Human | Att581 = L | 2.673% | 100% | CBA |
| Human | Att409 = N and Att37 = S | 2.673% | 100% | DT |
| Human | Att28 = S | 2.450% | 100% | CBA |
| Human | Att465 = V | 2.450% | 100% | CBA |
| Human | Att140 = T | 2.227% | 100% | CBA |
| Human | Att379 = L | 2.227% | 100% | CBA |
| Human | Att252 = K | 2.004% | 100% | CBA |
| Human | Att70 = V and Att65 = S | 2.004% | 100% | CBA |
| Human | Att226 = I and Att38 = I | 1.782% | 100% | CBA |
| Human | Att394 = N and Att63 = I | 1.782% | 100% | CBA |
| Human | Att712 = T and Att58 = S | 1.559% | 100% | CBA |
| Human | Att361 = R and Att61 = I | 1.559% | 100% | CBA |
| Human | Att62 = M | 1.336% | 100% | CBA |
| Human | Att550 = I and Att29 = K | 1.336% | 100% | CBA |
| Human | Att208 = A | 1.114% | 100% | CBA |
| Human | Att287 = S | 1.114% | 100% | CBA |
| Human | Att609 = R | 1.114% | 100% | CBA |
| Human | Att395 = G and Att14 = V | 1.114% | 100% | CBA |
| Human | Att204 = K and Att337 = A | 28.285% | 98.449% | Ripper |
| Human | Att367= K and Att346= Q and Att204= K | 35.857% | 98.170% | DT |
| Human | Att356 = R and Att55 = D | 20.490% | 97.872% | CBA |
| Swine | Att208 = K | 10.022% | 100% | CBA |
| Swine | Att85 = N | 6.904% | 100% | CBA |
| Swine | Att387 = V and Att20 = T | 6.682% | 100% | CBA |
| Swine | Att241 = Y and Att66 = G | 6.459% | 100% | CBA |
| Swine | Att263 = E | 6.236% | 100% | CBA |
| Swine | Att277 = F and Att62 = I | 6.236% | 100% | CBA |
| Swine | Att226 = F | 6.013% | 100% | CBA |
| Swine | Att689 = S | 6.013% | 100% | CBA |
| Swine | Att254 = S and Att28 = P | 4.009% | 100% | CBA |
| Swine | Att328 = R | 3.786% | 100% | CBA |
| Swine | Att388 = S and Att20 = T | 3.563% | 100% | CBA |
| Swine | Att350 = S and Att20 = A | 3.341% | 100% | CBA |
| Swine | Att581 = L | 2.673% | 100% | CBA |
| Swine | Att224 = P and Att58 = G | 2.450% | 100% | CBA |
| Swine | Att140 = T | 2.227% | 100% | CBA |
| Swine | Att66 = S and Att30 = I | 1.559% | 100% | CBA |
| Swine | Att323 = V and Att70 = V | 1.559% | 100% | CBA |
| Swine | Att441 = I | 1.336% | 100% | CBA |
| Swine | Att570 = I | 1.336% | 100% | CBA |
| Swine | Att379 = L and Att20 = A | 1.336% | 100% | CBA |
| Swine | Att208 = A | 1.114% | 100% | CBA |
| Swine | Att346 = K | 1.114% | 100% | CBA |
