# Supplementary material for: Novel approach for identification of influenza virus host range and zoonotic transmissible sequences by determination of host-related associative positions in viral genome segments
Source: BMC Genomics. 2016 Nov 16;17:925. doi: 10.1186/s12864-016-3250-9 (PMC5112743; doi:10.1186/s12864-016-3250-9)
Supplement: Additional file 11: Table S9. — Listing the rules extracted from PA-X protein of influenza A in identification of host ranges. (DOCX 21 kb) [file 12864_2016_3250_MOESM11_ESM.docx]

**Table S9.** Rules extracted from PA-X protein of influenza A in identification of host ranges

| **Class** | **Rule** | **Support** | **Confidence** | **Algorithm** |
| --- | --- | --- | --- | --- |
| Avian | Att250 = Q and Att193 = N | 18.710% | 100% | CBA |
| Avian | Att129 = T | 18.065% | 100% | CBA |
| Avian | Att204 = N and Att215 = Q | 12.830% | 100% | Ripper |
| Avian | Att101 = D and Att85 = T | 5.591% | 100% | CBA |
| Avian | Att214 = S and Att209 = E | 4.516% | 100% | CBA |
| Avian | Att241 = A and Att210 = Q | 2.796% | 100% | CBA |
| Avian | Att221 = R and Att100 = I | 2.366% | 100% | CBA |
| Avian | Att140 = T | 2.151% | 100% | CBA |
| Avian | Att85 = T and Att20 = T | 2.151% | 100% | CBA |
| Avian | Att248 = R and Att66 = G | 1.935% | 100% | CBA |
| Avian | Att184 = S and Att86 = V | 1.935% | 100% | CBA |
| Avian | Att210 = L and Att195 = R | 1.720% | 100% | CBA |
| Avian | Att222 = I and Att20 = A | 1.505% | 100% | CBA |
| Avian | Att235 = G | 1.290% | 100% | CBA |
| Avian | Att212 = E and Att20 = A | 1.290% | 100% | CBA |
| Avian | Att70 = A and Att37 = S | 1.290% | 100% | CBA |
| Avian | Att99 = R and Att44 = V | 1.290% | 100% | CBA |
| Avian | Att195 = R and Att61 = T | 1.290% | 100% | CBA |
| Avian | Att176 = Y and Att99 = G | 1.290% | 100% | CBA |
| Avian | Att100 = A and Att55 = D | 1.075% | 100% | CBA |
| Avian | Att250 = Q and Att199 = R | 33.763% | 99.367% | CBA |
| Avian | Att236 = S and Att101 = D | 8.172% | 97.436% | CBA |
| Avian | Att193 = N and Att29 = K | 19.570% | 96.809% | CBA |
| Human | Att204 = N and Att215 = Q | 12.830% | 100% | Ripper |
| Human | Att215 = Q and Att207 = S | 7.957% | 100% | CBA |
| Human | Att207 = L and Att28 = S | 4.905% | 100% | Ripper |
| Human | Att210 = Q and Att57 = Q | 4.086% | 100% | CBA |
| Human | Att227 = R and Att220 = H | 3.226% | 100% | CBA |
| Human | Att227 = R and Att62 = I | 3.011% | 100% | CBA |
| Human | Att236 = L and Att184 = S | 3.011% | 100% | CBA |
| Human | Att28 = S | 2.796% | 100% | CBA |
| Human | Att228 = T and Att227 = R | 2.366% | 100% | CBA |
| Human | Att140 = T | 2.151% | 100% | CBA |
| Human | Att100 = I and Att58 = S | 1.935% | 100% | CBA |
| Human | Att199 = K and Att63 = I | 1.935% | 100% | CBA |
| Human | Att193 = S and Att86 = V | 1.935% | 100% | CBA |
| Human | Att244 = A and Att221 = Q | 1.935% | 100% | CBA |
| Human | Att244 = A and Att224 = Q | 1.935% | 100% | CBA |
| Human | Att70 = V and Att65 = S | 1.720% | 100% | CBA |
| Human | Att227 = I and Att100 = V | 1.720% | 100% | CBA |
| Human | Att127 = I and Att65 = S | 1.505% | 100% | CBA |
| Human | Att220 = R and Att218 = A | 1.505% | 100% | CBA |
| Human | Att235 = G | 1.290% | 100% | CBA |
| Human | Att247 = P and Att58 = S | 1.290% | 100% | CBA |
| Human | Att218 = A and Att99 = E | 1.290% | 100% | CBA |
| Human | Att251 = K and Att212 = E | 1.290% | 100% | CBA |
| Human | Att221 = Q and Att104 = K | 1.075% | 100% | CBA |
| Human | Att213 = S and Att42 = L | 19.355% | 97.826% | CBA |
| Human | Att85 = I | 18.279% | 97.701% | DT |
| Human | Att250 = R and Att61 = I | 5.161% | 96.000% | CBA |
| Human | Att85 = A | 8.679% | 95.833% | DT |
| Swine | Att85 = N | 6.667% | 100% | CBA |
| Swine | Att204 = G and Att101 = E | 6.452% | 100% | CBA |
| Swine | Att99 = E | 6.237% | 100% | CBA |
| Swine | Att55 = N and Att20 = T | 6.022% | 100% | CBA |
| Swine | Att221 = Q | 5.806% | 100% | CBA |
| Swine | Att225 = V | 5.806% | 100% | CBA |
| Swine | Att224 = Q | 5.376% | 100% | CBA |
| Swine | Att63 = I and Att55 = N | 5.376% | 100% | CBA |
| Swine | Att65 = P and Att55 = N | 5.376% | 100% | CBA |
| Swine | Att208 = K | 5.161% | 100% | CBA |
| Swine | Att215 = L and Att55 = N | 5.161% | 100% | CBA |
| Swine | Att115 = D | 4.946% | 100% | CBA |
| Swine | Att204 = D and Att101 = G | 4.301% | 100% | CBA |
| Swine | Att196 = G | 3.226% | 100% | CBA |
| Swine | Att101 = E and Att20 = T | 2.581% | 100% | CBA |
| Swine | Att140 = T | 2.151% | 100% | CBA |
| Swine | Att193 = S and Att86 = V | 1.935% | 100% | CBA |
| Swine | Att207 = L and Att193 = N | 1.935% | 100% | CBA |
| Swine | Att66 = S and Att30 = I | 1.720% | 100% | CBA |
| Swine | Att220 = R and Att219 = F | 1.505% | 100% | CBA |
| Swine | Att204 = N and Att70 = V | 1.290% | 100% | CBA |
| Swine | Att215 = Q and Att58 = G | 1.075% | 100% | CBA |
| Swine | Att250 = Q and Att212 = V | 1.075% | 95.833% | CBA |
| Swine | Att213 = S and Att14 = V | 19.785% | 91.089% | CBA |
