# Supplementary material for: Novel approach for identification of influenza virus host range and zoonotic transmissible sequences by determination of host-related associative positions in viral genome segments
Source: BMC Genomics. 2016 Nov 16;17:925. doi: 10.1186/s12864-016-3250-9 (PMC5112743; doi:10.1186/s12864-016-3250-9)
Supplement: Additional file 12: Table S10. — Listing the rules extracted from PB1 protein of influenza A in identification of host ranges. (DOCX 20 kb) [file 12864_2016_3250_MOESM12_ESM.docx]

**Table S10.** Rules extracted from PB1 protein of influenza A in identification of host ranges

| **Class** | **Rule** | **Support** | **Confidence** | **Algorithm** |
| --- | --- | --- | --- | --- |
| Avian | Att621 = Q and Att149 = I | 19.383% | 100% | CBA |
| Avian | Att638 = E and Att14 = V | 18.722% | 100% | CBA |
| Avian | Att113 = I and Att215 = K | 15.198% | 100% | CBA |
| Avian | Att584 = R and Att758 = Q | 4.185% | 100% | CBA |
| Avian | Att584 = R and Att200 = I | 3.524% | 100% | CBA |
| Avian | Att435 = I and Att257 = A | 3.084% | 100% | CBA |
| Avian | Att581 = E and Att654 = N | 1.982% | 100% | CBA |
| Avian | Att694 = S | 1.762% | 100% | CBA |
| Avian | Att397 = I and Att302 = V | 1.762% | 100% | CBA |
| Avian | Att397 = I and Att744 = L | 1.762% | 100% | CBA |
| Avian | Att397 = I and Att754 = G | 1.762% | 100% | CBA |
| Avian | Att384 = P | 1.322% | 100% | CBA |
| Avian | Att567 = H | 1.322% | 100% | CBA |
| Avian | Att621 = Q and Att587 = T | 1.322% | 100% | CBA |
| Avian | Att581 = E and Att576 = I | 1.322% | 100% | CBA |
| Avian | Att211 = R and Att375 = D | 1.101% | 100% | CBA |
| Avian | Att741 = A and Att586 = R | 1.101% | 100% | CBA |
| Avian | Att374 = A and Att14 = V | 21.145% | 98.969% | CBA |
| Avian | Att398 = D and Att215 = K | 17.401% | 97.531% | CBA |
| Avian | Att375 = N | 34.802% | 87.293% | CBA |
| Avian | Att430 = R | 41.850% | 84.821% | CBA |
| Human | Att339 = I and Att400 = A | 4.405% | 100% | CBA |
| Human | Att158 = N and Att400 = A | 4.185% | 100% | CBA |
| Human | Att54 = K and Att584 = H | 3.744% | 100% | CBA |
| Human | Att200 = V and Att102 = L | 3.084% | 100% | CBA |
| Human | Att375 = N and Att172 = D | 3.084% | 100% | CBA |
| Human | Att619 = N | 2.863% | 100% | CBA |
| Human | Att374 = A and Att573 = A | 2.863% | 100% | CBA |
| Human | Att368 = V | 2.643% | 100% | CBA |
| Human | Att213 = N and Att114 = I | 2.643% | 100% | CBA |
| Human | Att642 = S and Att433 = R | 2.643% | 100% | CBA |
| Human | Att401 = V | 2.423% | 100% | CBA |
| Human | Att525 = V | 2.423% | 100% | CBA |
| Human | Att517 = V and Att298 = V | 1.982% | 100% | CBA |
| Human | Att430 = R and Att108 = I | 1.762% | 100% | CBA |
| Human | Att121 = R | 1.542% | 100% | CBA |
| Human | Att621 = Q and Att667 = T | 1.542% | 100% | CBA |
| Human | Att682 = V | 1.322% | 100% | CBA |
| Human | Att175 = D and Att76 = N | 1.322% | 100% | CBA |
| Human | Att149 = I and Att591 = I | 1.101% | 100% | CBA |
| Human | Att214 = R and Att584 = H | 1.101% | 100% | CBA |
| Human | Att12 = I | 20.044% | 97.849% | CBA |
| Human | Att75 = E and Att586 = R | 4.405% | 95.238% | CBA |
| Human | Att654 = S and Att353 = R | 3.304% | 93.750% | CBA |
| Human | Att654 = S and Att756 = P | 2.863% | 92.857% | CBA |
| Human | Att157 = A and Att400 = A | 5.507% | 92.593% | CBA |
| Swine | Att386 = R and Att152 = L | 10.132% | 100% | CBA |
| Swine | Att211 = K and Att52 = R | 9.471% | 100% | CBA |
| Swine | Att342 = I and Att383 = D | 8.370% | 100% | CBA |
| Swine | Att12 = V and Att517 = V | 6.388% | 100% | CBA |
| Swine | Att642 = N and Att433 = R | 4.626% | 100% | CBA |
| Swine | Att581 = D and Att257 = A | 3.744% | 100% | CBA |
| Swine | Att678 = N | 1.101% | 100% | CBA |
| Swine | Att736 = K and Att517 = V | 23.348% | 91.379% | CBA |
|  | **Iteration 2** |  |  |  |
| Avian | Att741 = A and Att14 = A | 33.813% | 100% | CBA |
| Avian | Att433 = R | 3.597% | 100% | CBA |
| Avian | Att121 = R | 2.158% | 100% | CBA |
| Avian | Att632 = A | 2.158% | 100% | CBA |
| Human | Att744 = T | 2.898% | 100% | CBA |
