# Supplementary material for: Novel approach for identification of influenza virus host range and zoonotic transmissible sequences by determination of host-related associative positions in viral genome segments
Source: BMC Genomics. 2016 Nov 16;17:925. doi: 10.1186/s12864-016-3250-9 (PMC5112743; doi:10.1186/s12864-016-3250-9)
Supplement: Additional file 13: Table S11. — Listing the rules extracted from PB1-F2 protein of influenza in identification of host ranges. (DOCX 20 kb) [file 12864_2016_3250_MOESM13_ESM.docx]

**Table S11.** Rules extracted from PB1-F2 protein of influenza A in identification of host ranges

| **Class** | **Rule** | **Support** | **Confidence** | **Algorithm** |
| --- | --- | --- | --- | --- |
| Avian | Att73 = K and Att65 = K and Att69 = Q and Att67 = P | **55.660** | 100% | Decision Tree |
| Avian | Att59 = K and Att28 = Q | 48.585% | 100% | CBA |
| Avian | Att59 = K and Att47 = S | 47.170% | 100% | CBA |
| Avian | Att76 = V and Att35 = S | 45.283% | 100% | CBA |
| Avian | Att79 = R and Att35 = S | 44.811% | 100% | Ripper |
| Avian | Att69 = Q and Att59 = K and Att2 = E | 42.925% | 100% | CBA |
| Avian | Att60 = Q and Att45 = I | 40.094% | 100% | CBA |
| Avian | Att55 = I and Att47 = S | 37.736% | 100% | CBA |
| Avian | Att81 = K and Att55 = I and Att1 = M | 37.264% | 100% | CBA |
| Human | Att48 = P and Att6 = D and Att3 = Q | 21.226% | 100% | CBA |
| Human | Att55 = I and Att49 = V and Att2 = E | 18.868% | 100% | CBA |
| Human | Att57 = Y and Att28 = Q and Att2 = E | 17.925% | 100% | CBA |
| Human | Att50 = D and Att27 = I | 16.509% | 100% | CBA |
| Human | Att57 = Y and Att50 = G and Att1 = M | 12.736% | 100% | CBA |
| Human | Att6 = D and Att3 = Q and Att2 = G | 12.264% | 100% | CBA |
| Human | Att76 = A and Att29 = K | 11.321% | 100% | CBA |
| Human | Att75 = H and Att20 = R | 7.075% | 100% | CBA |
| Human | Att89 = I and Att37 = Q | 7.075% | 100% | CBA |
| Human | Att57 = Y and Att50 = D | 6.604% | 100% | CBA |
| Human | Att84 = S and Att57 = Y | 6.604% | 100% | CBA |
| Human | Att70 = V and Att63 = S | 6.604% | 100% | CBA |
| Human | Att29 = K and Att20 = R | 5.189% | 100% | CBA |
| Human | Att56 = V and Att54 = R | 5.189% | 100% | CBA |
| Human | Att32 = R and Att23 = S and Att2 = G | 5.189% | 100% | CBA |
| Human | Att28 = P | 5.188% | 100% | DT |
| Human | Att60 = R | 4.717% | 100% | CBA |
| Human | Att70 = G and Att32 = R | 4.717% | 100% | CBA |
| Human | Att65 = R and Att59 = K | 4.717% | 100% | CBA |
| Human | Att18 = T and Att14 = G and Att3 = Q | 4.717% | 100% | CBA |
| Human | Att57 = F and Att47 = S | 3.774% | 100% | CBA |
| Human | Att77 = S and Att66 = N | 3.774% | 100% | CBA |
| Human | Att81 = R and Att40 = G and Att1 = M | 1.887% | 100% | CBA |
| Swine | Att71 = Y and Att44 = R | 8.962% | 100% | CBA |
| Swine | Att21=K and Att44=R and Att75=H | 8.962% | 100% | Ripper |
| Swine | Att79 = Q and Att45 = T | 6.132% | 100% | CBA |
| Swine | Att57 = F and Att47 = N | 6.132% | 100% | CBA |
| Swine | Att65 = R and Att1 = - | 5.189% | 100% | CBA |
| Swine | Att50 = V and Att48 = Q | 5.189% | 100% | CBA |
| Swine | Att71 = F and Att54 = Q | 5.189% | 100% | CBA |
| Swine | Att55 = I and Att29 = K | 4.717% | 100% | CBA |
| Swine | Att86 = H and Att83 = F | 3.774% | 100% | CBA |
| Swine | Att37 = R and Att14 = G | 3.302% | 100% | CBA |
| Swine | Att32 = H and Att4 = E and Att2 = G | 3.302% | 100% | CBA |
| Swine | Att74 = T and Att16 = I and Att2 = G | 3.302% | 100% | CBA |
| Swine | Att35 = L and Att10 = I | 2.830% | 100% | CBA |
| Swine | Att21 = K and Att6 = G | 1.415% | 100% | CBA |
