# Supplementary material for: Novel approach for identification of influenza virus host range and zoonotic transmissible sequences by determination of host-related associative positions in viral genome segments
Source: BMC Genomics. 2016 Nov 16;17:925. doi: 10.1186/s12864-016-3250-9 (PMC5112743; doi:10.1186/s12864-016-3250-9)
Supplement: Additional file 14: Table S12. — Listing the rules extracted from PB2 protein of influenza in identification of host ranges. (DOCX 22 kb) [file 12864_2016_3250_MOESM14_ESM.docx]

**Table S12.** Rules extracted from PB2 protein of influenza A in identification of host ranges

| **Class** | **Rule** | **Support** | **Confidence** | **Algorithm** |
| --- | --- | --- | --- | --- |
| Avian | Att134 = A | 11.682% | 100% | CBA |
| Avian | Att401 = Q | 7.944% | 100% | CBA |
| Avian | Att484 = T | 7.710% | 100% | CBA |
| Avian | Att131 = A | 5.140% | 100% | CBA |
| Avian | Att743 = R and Att31 = K | 4.439% | 100% | CBA |
| Avian | Att516 = V | 3.738% | 100% | CBA |
| Avian | Att571 = I | 2.336% | 100% | CBA |
| Avian | Att679 = V and Att18 = D | 2.336% | 100% | CBA |
| Avian | Att498 = S | 2.103% | 100% | CBA |
| Avian | Att59 = R | 1.402% | 100% | CBA |
| Avian | Att388 = I | 1.402% | 100% | CBA |
| Avian | Att504 = A | 1.402% | 100% | CBA |
| Avian | Att503 = N and Att18 = D | 1.402% | 100% | CBA |
| Avian | Att131 = I and Att26 = R | 1.402% | 100% | CBA |
| Avian | Att116 = I | 1.168% | 100% | CBA |
| Avian | Att261 = V | 1.168% | 100% | CBA |
| Avian | Att412 = K | 1.168% | 100% | CBA |
| Avian | Att554 = L | 1.168% | 100% | CBA |
| Avian | Att216 = A and Att80 = K | 1.168% | 100% | CBA |
| Avian | Att759 = M | 3.271% | 92.857% | CBA |
| Avian | Att107 = A | 2.804% | 92.308% | CBA |
| Avian | Att223 = K and Att80 = K | 3.037% | 86.667% | CBA |
| Avian | Att506 = V | 1.402% | 85.714% | CBA |
| Avian | Att759 = I and Att18 = D | 1.402% | 85.714% | CBA |
| Avian | Att744 = A and Att80 = K | 46.729% | 85.106% | CBA |
| Human | Att134 = A | 11.682% | 100% | CBA |
| Human | Att401 = R and Att743 = R | 4.673% | 100% | CBA |
| Human | Att557 = N and Att140 = I | 3.505% | 100% | CBA |
| Human | Att90 = I and Att767 = T | 3.505% | 100% | CBA |
| Human | Att634 = I | 3.271% | 100% | CBA |
| Human | Att324 = V and Att223 = K | 3.271% | 100% | CBA |
| Human | Att131 = M | 2.804% | 100% | CBA |
| Human | Att422 = K and Att223 = K | 2.804% | 100% | CBA |
| Human | Att153 = R | 2.570% | 100% | CBA |
| Human | Att430 = V | 2.570% | 100% | CBA |
| Human | Att108 = T | 2.103% | 100% | CBA |
| Human | Att368 = F | 1.402% | 100% | CBA |
| Human | Att372 = N | 1.402% | 100% | CBA |
| Human | Att347 = I | 19.860% | 97.701% | CBA |
| Human | Att782 = K and Att387 = K | 3.271% | 93.333% | CBA |
| Human | Att216 = T and Att350 = K | 2.804% | 92.308% | CBA |
| Human | Att324 = T | 14.019% | 89.552% | CBA |
| Human | Att652 = T | 21.729% | 86.916% | CBA |
| Swine | Att732 = I and Att331 = K | 7.477% | 100% | CBA |
| Swine | Att508 = M and Att513 = I | 6.542% | 100% | CBA |
| Swine | Att732 = I and Att784 = N | 6.308% | 100% | CBA |
| Swine | Att324 = I and Att331 = K | 6.075% | 100% | CBA |
| Swine | Att372 = R and Att132 = A | 5.841% | 100% | CBA |
| Swine | Att92 = T | 5.374% | 100% | CBA |
| Swine | Att508 = M and Att135 = I | 5.374% | 100% | CBA |
| Swine | Att486 = S and Att743 = R | 4.206% | 100% | CBA |
| Swine | Att508 = M and Att484 = V | 3.972% | 100% | CBA |
| Swine | Att494 = I and Att107 = A | 2.804% | 100% | CBA |
| Swine | Att102 = T and Att744 = V | 2.804% | 100% | CBA |
| Swine | Att90 = M and Att823 = N | 2.804% | 100% | CBA |
| Swine | Att131 = T and Att476 = R | 2.570% | 100% | CBA |
| Swine | Att554 = Q and Att671 = I | 2.570% | 100% | CBA |
| Swine | Att257 = G and Att259 = I | 2.570% | 100% | CBA |
| Swine | Att149 = A | 1.636% | 100% | CBA |
| Swine | Att303 = A and Att287 = I | 1.636% | 100% | CBA |
| Swine | Att59 = R | 1.402% | 100% | CBA |
| Swine | Att108 = N and Att259 = I | 2.804% | 92.308% | CBA |
| Swine | Att257 = G | 20.794% | 87.255% | CBA |
|  | **Iteration2** |  |  |  |
| Avian | Att179 = I and Att303 = T | 31.319% | 98.276% | CBA |
| Avian | Att108 = N and Att759 = I | 2.747% | 100% | CBA |
| Avian | Att511 = I and Att303 = T | 13.187% | 100% | CBA |
| Avian | Att108 = N and Att259 = I | 2.198% | 100% | CBA |
| Human | Att372 = K and Att70 = S | 6.000% | 100% | CBA |
| Human | Att92 = I | 2.000% | 100% | CBA |
| Human | Att617 = V and Att18 = N | 2.000% | 100% | CBA |
| Human | Att133 = N and Att70 = S | 5.000% | 90.909% | CBA |
| Human | Att106 = R and Att31 = R | 4.000% | 80.000% | CBA |
| Human | Att18 = Y | 2.000% | 100% | DT |
| Swine | Att91 = D and Att223 = E | 29.230% | 89.062% | Ripper |
