# Supplementary material for: Novel approach for identification of influenza virus host range and zoonotic transmissible sequences by determination of host-related associative positions in viral genome segments
Source: BMC Genomics. 2016 Nov 16;17:925. doi: 10.1186/s12864-016-3250-9 (PMC5112743; doi:10.1186/s12864-016-3250-9)
Supplement: Additional file 15: Tables S13–S15. — Which contain integrated rules based on whole segments of influenza to identify human, avian, and swine hosts. (DOCX 20 kb) [file 12864_2016_3250_MOESM15_ESM.docx]

Table S13 Combined rules extracted from influenza A strain protein of all segments to identify Avian Host

| **Confidence** | **Support** | **Rule** |
| --- | --- | --- |
| 100% | 30.861% | HA_Att444 = D |
| 100% | 22.255% | HA_Att540 = R and HA_Att9 = - |
| 100% | 21.513% | HA_Att540 = R and HA_Att10 = M |
| 100% | 17.953% | HA_Att117 = N and HA_Att15 = L |
| 100% | 17.507% | M1_Att115 = V and HA_Att9 = - |
| 100% | 16.320% | NS1_Att230 = S and HA_Att14 = V |
| 100% | 16.172% | HA_Att121 = D and HA_Att117 = N |
| 100% | 16.024% | HA_Att591 = V and HA_Att117 = N |
| 100% | 15.875% | NS1_Att91 = T and HA_Att10 = M |
| 100% | 15.282% | NS1_Att229 = E and HA_Att117 = N |
| 100% | 14.688% | NA_Att85 = L |
| 100% | 14.392% | PB1_Att257 = T and HA_Att121 = D |
| 100% | 14.243% | NA_Att38 = I and HA_Att9 = - |
| 100% | 12.463% | NA_Att364 = Y and HA_Att117 = N |
| 100% | 12.166% | NA_Att74 = - and HA_Att117 = N |
| 100% | 11.869% | HA_Att194 = A and HA_Att117 = N |
| 100% | 10.979% | PB1_F2_Att76 = V and HA_Att14 = V |
| 100% | 9.941% | HA_Att194 = T and HA_Att8 = - |
| 100% | 9.941% | HA_Att223 = T and HA_Att8 = M |
| 100% | 8.309% | HA_Att591 = I and HA_Att121 = G |
| 100% | 7.418% | HA_Att389 = L and HA_Att11 = I |
| 100% | 7.270% | NA_Att464 = N and HA_Att35 = K |
| 100% | 7.122% | PA_X_Att20 = - and HA_Att35 = K |
| 100% | 3.858% | NA_Att464 = N and HA_Att9 = N |
| 100% | 3.561% | HA_Att30 = V |
| 100% | 3.412% | HA_Att508 = R and HA_Att13 = I |
| 100% | 3.412% | HA_Att389 = L and HA_Att15 = S |
| 100% | 3.116% | HA_Att389 = L and HA_Att9 = K |
| 100% | 3.116% | HA_Att508 = R and HA_Att117 = N |
| 100% | 2.967% | HA_Att242 = S |
| 100% | 2.671% | HA_Att223 = D |
| 100% | 2.522% | HA_Att224 = E and HA_Att15 = V |
| 100% | 2.522% | HA_Att286 = I and HA_Att129 = I |
| 100% | 2.226% | NS1_Att93 = I |
| 100% | 1.929% | PB1_F2_Att73 = K and HA_Att11 = I |
| 100% | 1.632% | NS1_Att103 = L and HA_Att121 = G |
| 100% | 1.187% | HA_Att331 = E |
| 100% | 1.187% | NP_Att412 = V and HA_Att121 = G |

Table S14 Combined rules extracted from influenza A strain protein of all segments to identify Human Host

| **Confidence** | **Support** | **Rule** |
| --- | --- | --- |
| 100% | 8.754% | HA_Att194 = T and HA_Att16 = L |
| 100% | 8.605% | HA_Att176 = K and HA_Att9 = - |
| 100% | 6.973% | HA_Att194 = K and HA_Att9 = K |
| 100% | 6.231% | M2_Att66 = A |
| 100% | 6.083% | NA_Att387 = L and HA_Att9 = K |
| 100% | 5.786% | PB2_Att134 = A |
| 100% | 5.341% | HA_Att571 = I and HA_Att13 = I |
| 100% | 5.045% | NS2_Att14 = V and HA_Att13 = I |
| 100% | 4.303% | HA_Att173 = L and HA_Att13 = I |
| 100% | 3.858% | NA_Att454 = K and HA_Att9 = N |
| 100% | 3.709% | NA_Att207 = I and HA_Att9 = K |
| 100% | 3.709% | NA_Att454 = E and HA_Att13 = A |
| 100% | 3.412% | NA_Att26 = I and HA_Att9 = N |
| 100% | 3.412% | PB1_F2_Att84 = S and HA_Att13 = L |
| 100% | 3.412% | NS1_Att59 = L and HA_Att14 = F |
| 100% | 3.116% | HA_Att220 = V |
| 100% | 2.819% | NA_Att356 = M and HA_Att17 = L |
| 100% | 2.671% | NP_Att359 = L |
| 100% | 2.671% | NP_Att412 = V |
| 100% | 2.671% | M1_Att137 = A and HA_Att8 = - |
| 100% | 2.671% | PB1_Att375 = N and HA_Att9 = N |
| 100% | 2.522% | HA_Att71 = V |
| 100% | 2.522% | HA_Att222 = N and HA_Att8 = - |
| 100% | 2.522% | PB2_Att324 = T and HA_Att8 = - |
| 100% | 2.522% | NA_Att53 = G and HA_Att9 = M |
| 100% | 2.522% | HA_Att434 = K and HA_Att11 = A |
| 100% | 2.522% | PA_X_Att199 = K and HA_Att16 = L |
| 100% | 2.374% | NA_Att51 = K |
| 100% | 2.374% | PA_X_Att227 = R and HA_Att8 = - |
| 100% | 2.374% | HA_Att19 = T and HA_Att10 = M |
| 100% | 2.374% | HA_Att448 = I and HA_Att14 = L |
| 100% | 2.374% | HA_Att140 = G and HA_Att14 = A |
| 100% | 2.226% | HA_Att33 = M |
| 100% | 2.077% | HA_Att359 = K and HA_Att12 = A |
| 100% | 2.077% | HA_Att223 = A and HA_Att14 = I |
| 100% | 2.077% | NA_Att95 = V and HA_Att14 = L |
| 100% | 2.077% | PB1_Att54 = R and HA_Att17 = L |
| 100% | 1.929% | PB2_Att634 = I |
| 100% | 1.929% | PB2_Att223 = K and HA_Att12 = I |
| 100% | 1.780% | HA_Att273 = S and HA_Att8 = - |
| 100% | 1.780% | PB2_Att652 = I and HA_Att8 = - |
| 100% | 1.632% | PB1_Att619 = N |
| 100% | 1.632% | HA_Att163 = - and HA_Att9 = M |
| 100% | 1.632% | NP_Att359 = I and HA_Att11 = E |
| 100% | 1.484% | HA_Att374 = G |
| 100% | 1.484% | PA_Att224 = P |
| 100% | 1.484% | NP_Att358 = M and HA_Att12 = I |
| 100% | 1.335% | PA_Att581 = L |
| 100% | 1.335% | HA_Att358 = S and HA_Att8 = - |
| 100% | 1.335% | HA_Att207 = E and HA_Att11 = V |
| 100% | 1.187% | HA_Att260 = X |
| 100% | 1.187% | HA_Att259 = E and HA_Att10 = K |
| 100% | 1.039% | NS2_Att52 = V |
| 100% | 1.039% | HA_Att16 = M and HA_Att8 = - |
| 100% | 0.890% | NA_Att96 = A |
| 100% | 0.890% | NP_Att107 = G |
| 100% | 0.890% | PB2_Att372 = N |
| 100% | 0.890% | PB1_Att667 = T and HA_Att9 = - |
| 100% | 0.742% | HA_Att222 = V |
| 100% | 0.742% | HA_Att259 = X |
| 100% | 0.742% | HA_Att223 = E and HA_Att13 = I |
| 100% | 0.593% | PB1_F2_Att32 = R and HA_Att9 = K |
| 98.611% | 21.068 | HA_Att223 = A and HA_Att163 = K |
| 98.701% | 11.276% | PB1_Att12 = I and HA_Att9 = M |
| 98.450% | 18.843% | HA_Att434 = E |
| 97.802% | 13.205% | NA_Att410 = I and HA_Att14 = V |
| 96.667% | 4.303% | HA_Att510 = K and HA_Att14 = V |

Table S15 Combined rules extracted from influenza A strain protein of all segments to identify Swine Host

| **Confidence** | **Support** | **Rule** |
| --- | --- | --- |
| 100% | 17.211% | HA_Att240 = S and HA_Att222 = S |
| 100% | 12.908% | HA_Att177 = N and HA_Att9 = M |
| 100% | 12.463% | NS1_Att125 = I and HA_Att9 = M |
| 100% | 12.463% | NS1_Att125 = I and HA_Att11 = A |
| 100% | 10.682% | NS1_Att125 = I and HA_Att234 = A |
| 100% | 9.347% | M1_Att207 = S and HA_Att9 = M |
| 100% | 9.050% | PB1_Att12 = V and HA_Att9 = M |
| 100% | 8.309% | NA_Att394 = I and HA_Att177 = N |
| 100% | 6.825% | HA_Att223 = T and HA_Att11 = A |
| 100% | 6.231% | PB1_Att152 = L and HA_Att177 = N |
| 100% | 6.083% | PA_Att208 = K |
| 100% | 5.638% | HA_Att448 = I |
| 100% | 5.490% | HA_Att19 = A and HA_Att8 = - |
| 100% | 5.341% | NP_Att431 = I and HA_Att9 = M |
| 100% | 5.193% | NS1_Att26 = G and HA_Att14 = L |
| 100% | 5.193% | NA_Att85 = V and HA_Att177 = N |
| 100% | 5.045% | HA_Att52 = I and HA_Att9 = M |
| 100% | 4.896% | NS2_Att63 = E and HA_Att14 = L |
| 100% | 4.748% | NA_Att85 = V and HA_Att52 = I |
| 100% | 4.451% | HA_Att331 = H and HA_Att11 = A |
| 100% | 4.451% | NA_Att364 = N and HA_Att14 = L |
| 100% | 4.451% | NS1_Att41 = K and HA_Att223 = S |
| 100% | 4.303% | M2_Att28 = A |
| 100% | 4.154% | HA_Att324 = T and HA_Att13 = L |
| 100% | 4.154% | NP_Att203 = I and HA_Att223 = S |
| 100% | 4.006% | NS2_Att86 = K and HA_Att177 = N |
| 100% | 3.858% | PB1_Att517 = V and HA_Att222 = D |
| 100% | 3.709% | PA_Att226 = F |
| 100% | 3.709% | NP_Att195 = M and HA_Att9 = M |
| 100% | 3.561% | M2_Att14 = G and HA_Att11 = A |
| 100% | 3.412% | NA_Att106 = V and HA_Att223 = S |
| 100% | 3.264% | NS1_Att223 = E |
| 100% | 3.264% | PB2_Att92 = T |
| 100% | 2.819% | PB1_Att257 = A and HA_Att11 = A |
| 100% | 2.819% | NA_Att61 = I and HA_Att222 = N |
| 100% | 2.671% | PB2_Att743 = R and HA_Att9 = M |
| 100% | 2.671% | PB1_F2_Att37 = R and HA_Att13 = L |
| 100% | 2.522% | NS1_Att228 = T |
| 100% | 2.374% | HA_Att235 = K |
| 100% | 2.077% | HA_Att234 = A and HA_Att52 = I |
| 100% | 1.780% | NS1_Att44 = K and HA_Att222 = N |
| 100% | 1.632% | HA_Att226 = K |
| 100% | 1.335% | NP_Att363 = R and HA_Att14 = L |
| 100% | 1.335% | NA_Att52 = D and HA_Att222 = D |
| 100% | 1.187% | HA_Att260 = X |
| 100% | 1.187% | HA_Att563 = I and HA_Att13 = I |
| 100% | 1.039% | NA_Att21 = L and HA_Att15 = F |
| 100% | 1.039% | PB1_F2_Att21 = K and HA_Att19 = V |
| 100% | 0.890% | HA_Att313 = R and HA_Att15 = I |
| 96.551% | 24.925% | HA_Att9 = M and HA_Att240 = S |
| 96.551% | 24.925% | HA_Att377 = Q and HA_Att240 = S |
