# Supplementary material for: Novel approach for identification of influenza virus host range and zoonotic transmissible sequences by determination of host-related associative positions in viral genome segments
Source: BMC Genomics. 2016 Nov 16;17:925. doi: 10.1186/s12864-016-3250-9 (PMC5112743; doi:10.1186/s12864-016-3250-9)
Supplement: Additional file 16: Table S16. — Listing the properties of some of the critical (informative) spots in the determination of influenza host ranges, extracted by associative classification rule mining (in this study), which correspond with similar position in other studies. (DOCX 31 kb) [file 12864_2016_3250_MOESM16_ESM.docx]

**Table S16.** Properties of some of the critical (informative) spots in determination of influenza host ranges, extracted by associative classification rule mining (in this study), that correspond with similar position in other studies

| **Segment** | **Position** | **Comment** | **Method** | **Host Range of the positions in current study** |
| --- | --- | --- | --- | --- |
| HA | 13[[1](#_ENREF_1)],[[2](#_ENREF_2)] | Amino acid differences in HPAI H7N7 viruses isolated in the Netherlands in 2003 (Human and Avian) |  | Human, Avian, Swine |
|  | 129[[3](#_ENREF_3)] | This position is various between duck, chicken and human H5N1 genotype Z influenza viruses from southern China and Southeast Asia 2002–2005 |  | Avian |
|  | 158 [[4](#_ENREF_4), [5](#_ENREF_5)] | Residues differences observed among human and chicken |  | Avian |
|  | 194[[3](#_ENREF_3)] | Conserved Amino Acids in the Globular Head of the Avian HA That Differ in the HA of Human Viruses. A total of 85 avian HA1 sequences were studied. | Competitive assay of virus binding of soluble receptor analogues.  Virus binding to gangliosides in the microwell adsorption assay | Human, Avian |
|  | 222[[6](#_ENREF_6), [7](#_ENREF_7)] | In H5N1 viruses recently isolated from humans and poultry in Thailand and Vietnam, amino acid residues at the receptor binding pocket of HA1,—that is, positions 222 and 224 retain configurations predicted to have affinity for avian cell-surface receptors. |  | Human, Swine |
|  | 224[[6](#_ENREF_6), [8](#_ENREF_8)] |  |  | Avian |
|  | 226  [[3-5](#_ENREF_3), [9](#_ENREF_9)] | This position is various between A/equine/Kentucky/1/91 and A/Udorn/307/72 | Genotyping of reassortant viruses by using reverse tran-scription-PCR.  Immunologic detection of SAa2,3Gal and SAa2,6Gal in animal tissues.  SA determination by liquid chromatography. | Swine |
| M1 [[10](#_ENREF_10)] | 15 | This position is various between A/PR/8/34 , A/FPV/Rostock/34 and A/Bangkok/1/79 , A/Udorn/307/72 ,A/mallard/NY/6750/78 | extending a series of synthetic oligonucleotide primers by reverse transcription with dideoxynucleotides | Human, Swine |
|  | 59 | This position is various between A/FPV/Rostock/34 and A/PR/8/34, A/Bangkok/1/79 , A/Udorn/307/72 ,A/mallard/NY/6750/78 |  | Avian |
|  |  |  |  | “Avian-Swine” |
|  | 95 | This position is various between A/PR/8/34 , A/FPV/Rostock/34 and A/Bangkok/1/79 , A/Udorn/307/72 ,A/mallard/NY/6750/78 |  | Avian, Swine |
|  | 115 | divergence between influenza A/Udorn/307/72 and A/mallard/NY/6750/78 |  | Avian |
|  | 116 | This position is various between A/PR/8/34 and A/FPV/Rostock/34 , A/Bangkok/1/79 , A/Udorn/307/72 ,A/mallard/NY/6750/78 |  | Human, Swine |
|  | 121 | divergence between influenza A/Udorn/307/72 and A/mallard/NY/6750/78 |  | Avian |
|  | 167 |  |  | Human |
|  | 181 | This position is various between A/FPV/Rostock/34 and A/PR/8/34, A/Bangkok/1/79 , A/Udorn/307/72 ,A/mallard/NY/6750/78 |  | Swine |
|  | 239 | This position is various between , A/Bangkok/1/79 and A/FPV/Rostock/34 , A/PR/8/34, A/Udorn/307/72 ,A/mallard/NY/6750/78 |  | Avian |
| M2 [[10](#_ENREF_10)] | 11 | divergence between influenza A/Udorn/307/72 and A/mallard/NY/6750/78 | extending a series of synthetic oligonucleotide primers by reverse transcription with dideoxynucleotides | Human |
|  | 14 |  |  | Human, Swine |
|  | 16 |  |  | Avian |
|  | 18 |  |  | Human, Avian, Swine |
|  | 20 |  |  | Avian, Swine |
|  | 21 | This position is various between A/PR/8/34 and A/FPV/Rostock/34 , A/Bangkok/1/79 , A/Udorn/307/72 ,A/mallard/NY/6750/78 |  | Avian |
|  | 27 | This position is various between A/PR/8/34 , A/FPV/Rostock/34 and A/Bangkok/1/79 , A/Udorn/307/72 ,A/mallard/NY/6750/78 |  | Human, Avian, Swine |
|  | 28 | divergence between influenza A/Udorn/307/72 and A/mallard/NY/6750/78 |  | Human, Swine |
|  | 31 | This position is various between A/PR/8/34 and A/FPV/Rostock/34 , A/Bangkok/1/79 , A/Udorn/307/72 ,A/mallard/NY/6750/78 |  | Avian, Swine |
|  | 44 | This position is various between A/FPV/Rostock/34 and A/Bangkok/1/79 A/PR/8/34 , A/Udorn/307/72 ,A/mallard/NY/6750/78 |  | Avian |
|  | 54 | Divergence between influenza A/Udorn/307/72 and A/mallard/NY/6750/78 |  | Avian, Swine |
|  | 55 |  |  | Human, Avian, Swine |
|  |  |  |  | “Human” |
|  |  |  |  | “Human”, “Human-Avian” |
|  | 61 | This position is various between A/PR/8/34  and A/Udorn/307/72 ,A/mallard/NY/6750/78,  A/Bangkok/1/79 , A/FPV/Rostock/34 |  | Avian |
|  | 78 | divergence between influenza A/Udorn/307/72 and A/mallard/NY/6750/78 |  | Human |
|  | 82 | This position is various between A/Bangkok/1/79 A/PR/8/34 and A/Udorn/307/72 ,A/mallard/NY/6750/78, , A/FPV/Rostock/34 |  | Human |
| PB1 | 152 | The PB1 of WI/88, which seems to result from the direct introduction of a swine influenza virus into a human, has serine at amino acid residue 375 as in all other human PB1 proteins except those of Beij/56, whereas other HlNl swine PB1 proteins have glycine at position 375 [[11](#_ENREF_11)].  Most avian viruses have Asn at position 375 of PB1, but 18% have Ser, and 13% Thr [[12](#_ENREF_12)]. | evolutionary tree constructed from nucleotide sequences | Swine |
|  | 157 |  |  | Human |
|  | 211 |  |  | Avian, Swine |
|  | 339 |  |  | Human |
|  | 375 |  |  | Human, Avian |
|  | 581 |  |  | Avian, Swine |
|  | 654 |  |  | Human, Avian |
|  | 397 [[13](#_ENREF_13)] | Residues differences observed among human and chicken H5N1 viruses isolated in Hong Kong | Phylogenetic analysis | Avian |
| PB1-F2 | 66 [[14](#_ENREF_14)] | Amino acid residue that distinguish human and avian influenza virus . |  | Human |
| PB2 [[15](#_ENREF_15)] | 134 | Reverse genetics was used to analyze the host range of two avian influenza viruses which differ in their ability to replicate in mouse and human cells in culture.  A/FPV/Dobson/27,A/FPV/Rostock-S3 | To assess the level of replication supported by different avian PB2 genes in a mammalian cell environment, we inserted the PB2 genes of Rostock and Dobson 4H viruses into the mammalian expression vector pcDNA3. Forty-eight hours posttransfection the cells were tested for the expression of chloramphenicol acetyltransferase (CAT) by enzyme-linked immunosorbent assay (ELISA; Roche Molecular Biochemicals) or enzyme assay | Human, Avian |
|  | 153 |  |  | Human |
| NS1 | 91 | Residues differences observed among human and chicken H5N1 viruses isolated in Hong Kong [[13](#_ENREF_13)]. | Phylogenetic analysis | Human, Avian |
|  | 125 |  |  | Human, Swine |
|  | 127 |  |  | Avian, Swine |
|  | 213 |  |  | Human |
| NS2 [[13](#_ENREF_13)] | 40 | Residues differences observed among human and chicken H5N1 viruses isolated in Hong Kong | Phylogenetic analysis | Human, Avian |
| PA | 55 [[12](#_ENREF_12)] | Amino acid residue that distinguish human and avian influenza virus polymerases identified by comparison of the genome of the human 1918 virus strain with those of other human, avian, swine, and equine viruses |  | “Human, Avian |
|  | 57 | This position is various between 15 human and 13 avian [[16](#_ENREF_16)]. | A genetic system that allows the in vivo reconstitution of active ribonucleoproteins | Human |
|  | 409 [[13](#_ENREF_13)] | Residues differences observed among human and chicken H5N1 viruses isolated in Hong Kong | Phylogenetic analysis | Human, Avian |
| NP [[13](#_ENREF_13)] | 377 | Residues differences observed among human and chicken H5N1 viruses isolated in Hong Kong | Phylogenetic analysis | Swine |

**References**

1. de Wit, E., et al., *Molecular determinants of adaptation of highly pathogenic avian influenza H7N7 viruses to efficient replication in the human host.* Journal of virology, 2010. **84**(3): p. 1597-1606.

2. Fouchier, R.A., et al., *Avian influenza A virus (H7N7) associated with human conjunctivitis and a fatal case of acute respiratory distress syndrome.* Proceedings of the National Academy of sciences of the United States of América, 2004. **101**(5): p. 1356-1361.

3. Matrosovich, M., et al., *Avian influenza A viruses differ from human viruses by recognition of sialyloligosaccharides and gangliosides and by a higher conservation of the HA receptor-binding site.* Virology, 1997. **233**(1): p. 224-234.

4. Imai, M., et al., *Experimental adaptation of an influenza H5 HA confers respiratory droplet transmission to a reassortant H5 HA/H1N1 virus in ferrets.* Nature, 2012. **486**(7403): p. 420-428.

5. Imai, M., et al., *Transmission of influenza A/H5N1 viruses in mammals.* Virus research, 2013. **178**(1): p. 15-20.

6. Suzuki, Y., *Sialobiology of influenza: molecular mechanism of host range variation of influenza viruses.* Biological and Pharmaceutical Bulletin, 2005. **28**(3): p. 399-408.

7. Yamnikova, S., et al., *Differences between HA receptor-binding sites of avian influenza viruses isolated from Laridae and Anatidae.* Avian diseases, 2003. **47**(s3): p. 1164-1168.

8. Gao, Y., et al., *Identification of amino acids in HA and PB2 critical for the transmission of H5N1 avian influenza viruses in a mammalian host.* PLoS pathogens, 2009. **5**(12): p. e1000709.

9. Suzuki, Y., et al., *Sialic acid species as a determinant of the host range of influenza A viruses.* Journal of virology, 2000. **74**(24): p. 11825-11831.

10. Buckler-White, A.J., C. Naeve, and B. Murphy, *Characterization of a gene coding for M proteins which is involved in host range restriction of an avian influenza A virus in monkeys.* Journal of virology, 1986. **57**(2): p. 697-700.

11. Kawaoka, Y., S. Krauss, and R.G. Webster, *Avian-to-human transmission of the PB1 gene of influenza A viruses in the 1957 and 1968 pandemics.* Journal of virology, 1989. **63**(11): p. 4603-4608.

12. Parrish, C.R., et al., *Cross-species virus transmission and the emergence of new epidemic diseases.* Microbiology and Molecular Biology Reviews, 2008. **72**(3): p. 457-470.

13. Hiromoto, Y., et al., *Evolutionary characterization of the six internal genes of H5N1 human influenza A virus.* Journal of General Virology, 2000. **81**(5): p. 1293-1303.

14. Neumann, G., T. Noda, and Y. Kawaoka, *Emergence and pandemic potential of swine-origin H1N1 influenza virus.* Nature, 2009. **459**(7249): p. 931-939.

15. Yao, Y., et al., *Sequences in influenza A virus PB2 protein that determine productive infection for an avian influenza virus in mouse and human cell lines.* Journal of virology, 2001. **75**(11): p. 5410-5415.

16. Naffakh, N., et al., *Genetic analysis of the compatibility between polymerase proteins from human and avian strains of influenza A viruses.* Journal of General Virology, 2000. **81**(5): p. 1283-1291.
