# Supplementary material for: Novel approach for identification of influenza virus host range and zoonotic transmissible sequences by determination of host-related associative positions in viral genome segments
Source: BMC Genomics. 2016 Nov 16;17:925. doi: 10.1186/s12864-016-3250-9 (PMC5112743; doi:10.1186/s12864-016-3250-9)
Supplement: Additional file 17: — Gives details of the developed approach in this study in increasing the support and accuracy of prediction and generation of more informative rules through making secondary datasets. The components of secondary datasets are discovered spots at the first run of rule discovery instead of sequence features. We extracted combinational association rules from several tables to gain rules with higher support and confidence. (DOCX 37 kb) [file 12864_2016_3250_MOESM17_ESM.docx]

**Supporting information.** Details of the developed approach in this study in increasing the support and accuracy of prediction and generation of more informative rules through making secondary dataset. The components of secondary dataset are important discovered spots at the first run of rule discovery instead of sequence features. We extracted combinational association rules from several tables to gain rules with higher support and confidence.

**Hypothesis**

In some situations, there are several information tables for one entity. These tables provide different information from different aspects about one subject. As an example, influenza virus has 12 proteins. Each protein sequence provides different information about this virus.

Extracting information from each table (protein sequences) is valuable. These extracted rules may have low support or confidence as we observed. If we extract rules from several tables synchronously and produce combinational rules, this can result in more reliable decision and prediction. Also, this provides higher support and confidence than compared to condition where the rules come from only one table.

**Important note:** This method is only applicable for the cases where we have more than one information table for one entity.

**Method (procedure)**

The following steps were taken to extract combinational rules from several tables:

**Step 1)**

Rules were extracted from each table, separately. To perform this, associative rule based classifier can be employed like CBA, Ripper, Decision tree, and etc.

If the class ratio is imbalanced, the next step should be executed; otherwise, you can continue from Step 3.

**Step 2)**

When the distribution of data is imbalance in different classes, after Step1, commonly the rules cover large classes and the small classes are unattended. To gain rules from small classes as well as large classes, under-sampling operation is suggested. The records will be removed which satisfy the step 1’s rules. Extraction rules and removing rows continue until all existing rules of dataset are extracted and all records (rows) will be removed.

For better explanation, we continue with the following example:

**Table1** – Part of the sequence of HA segment of influenza A virus related to the human and swine hosts

| **Target** | **HA10** | **HA9** | **HA8** | **HA7** | **HA6** | **HA5** | **HA4** | **HA3** | **HA2** | **HA1** | **Row** |
| --- | --- | --- | --- | --- | --- | --- | --- | --- | --- | --- | --- |
| **Swine** | D | N | M | Y | S | M | Y | E | M | G | **1** |
| **Swine** | D | A | M | D | T | C | A | E | T | G | **2** |
| **Swine** | N | G | M | T | S | G | F | A | M | G | **3** |
| **Human** | D | C | M | G | S | I | T | A | T | G | **4** |
| **Human** | D | C | M | T | S | M | Y | A | M | G | **5** |
| **Human** | D | T | D | C | C | G | A | A | T | G | **6** |
| **Human** | N | G | D | T | C | T | C | A | M | G | **7** |
| **Human** | N | A | D | F | C | D | N | E | T | T | **8** |
| **Human** | G | D | M | N | S | A | D | C | T | W | **9** |
| **Human** | G | A | Q | C | S | E | A | A | T | A | **10** |
| **Human** | G | W | M | T | S | Y | E | F | T | R | **11** |
| **Human** | T | Q | W | A | S | D | Y | C | T | W | **12** |
| **Human** | G | T | M | E | T | R | D | F | T | T | **13** |
| **Human** | G | V | M | D | G | T | R | F | T | D | **14** |
| **Human** | G | R | M | F | C | C | T | F | T | M | **15** |

Table 1 represents a part of sequences of HA segment of influenza. We suppose that this dataset is imbalanced. So, when common rule based classifier (CBA, RIPPER, Decision tree) are executed on the dataset (Step1 of current method), only large class’s rule can be extracted. Table2 illustrate the rules resulted from Step1.

**Table2** – Extracted rules from Table1 (HA segment) in the first step (Iteration 1)

| **Rule** | **Support** | **Confidence** |
| --- | --- | --- |
| HA2 = T and HA6 = S 🡺 Human | %33.33 | %100 |
| HA3 = F and HA10 = G 🡺 Human | %20 | %100 |

As mentioned before, we assume that algorithms are unable to extract more rules in this step. So, we start under-sampling operation. The records (rows) which these rules cover them are removed. i.e. (row 4, 9 -12 and row 13 - 15). Table 3 shows the remaining rows.

Table 3 – The rows which are remained after removing the rows corresponding to the found rules in larhe class (human) Table1.

| **Target** | **HA10** | **HA9** | **HA8** | **HA7** | **HA6** | **HA5** | **HA4** | **HA3** | **HA2** | **HA1** | **Row** |
| --- | --- | --- | --- | --- | --- | --- | --- | --- | --- | --- | --- |
| **Swine** | D | N | M | Y | S | M | Y | E | M | G | **1** |
| **Swine** | D | A | M | D | T | C | A | E | T | G | **2** |
| **Swine** | N | G | M | T | S | G | F | A | M | G | **3** |
| **Human** | D | C | D | T | S | M | Y | A | M | G | **5** |
| **Human** | D | T | D | C | C | G | A | A | T | G | **6** |
| **Human** | N | G | D | T | C | T | C | A | M | G | **7** |
| **Human** | N | A | D | F | C | D | N | E | T | T | **8** |

As shown in Table 3, the dataset is not imbalanced now. Thus, common rule based classifier algorithms can extract rules from both classes (as iteration2). Table 4 illustrate the rules resulted from Table3.

**Table 4** – Extracted rules from Table 3 in the second step (Iteration 2)

| **Rule** | **Support** | **Confidence** |
| --- | --- | --- |
| HA3 = E and HA10 = D 🡺 Swine | %28.57 | %100 |
| HA6 = C and HA8 = D 🡺 Human | %42.85 | %100 |
| HA8 = D and HA10 = D 🡺 Human | %28.57 | %100 |
| HA3 = A and HA10 = N 🡺 Swine | %14.28 | %100 |

Note: for iteration 2, support of rules can be calculated in 2 coditions:

1. According to the record’s number (rows) of new tables (Table 1). Supports of Table 4 were calculated based on this case.
2. According to the record’s number (rows) of main tables (Table 3).

Extracted rules of Table 4 cover all the records. So, the operation has performed completely well.

**Step 3)**

Repeat step1 and step2 for all tables.

We assume Table 5 is a part of NA segment of influenza A virus. So, we execute step1 and step2 as described above.

**Table 5** – Part of the sequence of NA segment of influenza A virus related to the human and swine host

| **Target** | **NA10** | **NA9** | **NA8** | **NA7** | **NA6** | **NA5** | **NA4** | **NA3** | **NA2** | **NA1** | **Row** |
| --- | --- | --- | --- | --- | --- | --- | --- | --- | --- | --- | --- |
| **Swine** | D | A | A | A | F | E | I | Y | M | F | **1** |
| **Swine** | N | I | N | N | M | E | I | E | I | T | **2** |
| **Swine** | N | D | G | G | E | N | I | A | W | T | **3** |
| **Human** | A | D | C | C | E | A | N | C | C | A | **4** |
| **Human** | G | F | E | E | C | G | N | A | A | A | **5** |
| **Human** | C | G | F | F | D | C | N | A | G | A | **6** |
| **Human** | G | D | E | E | D | G | N | A | C | A | **7** |
| **Human** | D | D | G | C | E | A | C | C | N | T | **8** |
| **Human** | C | C | A | C | E | C | C | N | A | T | **9** |
| **Human** | N | N | F | C | F | C | N | A | T | F | **10** |
| **Human** | T | G | G | D | F | E | C | A | T | W | **11** |
| **Human** | E | A | N | D | E | C | I | T | A | W | **12** |
| **Human** | A | D | F | D | E | D | I | A | T | W | **13** |
| **Human** | G | G | A | D | D | C | N | T | A | W | **14** |
| **Human** | A | T | E | D | F | D | I | N | G | W | **15** |

Table 6 demonstrates the extracted rules of Table 5 on iteration 1.

**Table 6** – Extracted rules from Table5 (NA segment) in the first step (Iteration 1)

| **Rule** | **Support** | **Confidence** |
| --- | --- | --- |
| NA1 = A and NA4 = N 🡺 Human | %26.66 | %100 |
| NA1 = W and NA7 = D 🡺 Human | %33.33 | %100 |

In order to extract rules for swine in Table 5, under-sampling operation carried out. Rows 4-7 and 11-15 were removed from Table 5. Rules of Table 6 cover these rows. Table 7 shows the remaining rows.

**Table 7** – Remaining rows of Table 5, after under-sampling operation.

| **Target** | **NA10** | **NA9** | **NA8** | **NA7** | **NA6** | **NA5** | **NA4** | **NA3** | **NA2** | **NA1** | **Row** |
| --- | --- | --- | --- | --- | --- | --- | --- | --- | --- | --- | --- |
| **Swine** | D | A | A | A | F | E | I | Y | M | F | **1** |
| **Swine** | N | I | N | N | M | E | I | E | I | T | **2** |
| **Swine** | N | D | G | G | E | N | I | A | W | T | **3** |
| **Human** | D | D | G | C | E | A | C | C | N | T | **8** |
| **Human** | C | C | A | C | E | C | C | N | A | T | **9** |
| **Human** | N | N | F | C | F | C | C | A | T | F | **10** |

Table 7 is a balanced dataset. Extracted rules of that shows in Table 8 (Iteration 2).

**Table 8** – Extracted rules from Table7 (NA segment) in the second step (Iteration 2)

| **Rule** | **Support** | **Confidence** |
| --- | --- | --- |
| NA1 = T and NA10 = N 🡺 Swine | %33.33 | %100 |
| NA4 = C and NA7 = C 🡺 human | %50 | %100 |
| NA1 = F and NA10 =D 🡺 Swine | %16.66 | %100 |

**Step 4)**

After extracting rules from all tables separately, we construct a new table using these rules as features. To this end, the constitutive columns of previous rules are selected. The selected columns of Table1 are: HA2, HA3, HA6, HA8, HA10 and for Table 5 are: NA1, NA4, NA7, NA10. Table 9 was constructed by using these columns.

**Table 9** – Combined table of informative columns of two tables HA, NA

| **Target** | **NA10** | **NA7** | **NA4** | **NA1** | **HA10** | **HA8** | **HA6** | **HA3** | **HA2** | **Row** |
| --- | --- | --- | --- | --- | --- | --- | --- | --- | --- | --- |
| **Swine** | D | A | I | F | D | M | S | E | M | **1** |
| **Swine** | N | N | I | T | D | M | T | E | T | **2** |
| **Swine** | N | G | I | T | N | M | S | A | M | **3** |
| **Human** | A | C | N | A | D | M | S | A | T | **4** |
| **Human** | G | E | N | A | D | M | S | A | M | **5** |
| **Human** | C | F | N | A | D | D | C | A | T | **6** |
| **Human** | G | E | N | A | N | D | C | A | M | **7** |
| **Human** | D | C | C | W | N | D | C | E | T | **8** |
| **Human** | C | C | C | W | G | M | S | C | T | **9** |
| **Human** | N | C | N | F | G | Q | S | A | T | **10** |
| **Human** | T | D | C | W | G | M | S | F | T | **11** |
| **Human** | E | D | I | W | T | W | S | C | T | **12** |
| **Human** | A | D | I | W | G | M | T | F | T | **13** |
| **Human** | G | D | N | W | G | M | G | F | T | **14** |
| **Human** | A | D | I | W | G | M | C | F | T | **15** |

**Step 5)**

After generation of new dataset in previous step, Step1 and Step 2 apply on this combined dataset.

We are executed common rule based classifier (CBA, RIPPER, Decision tree) on Table 9. Table 10 illustrates extracted rules from it.

**Table 10** – Extracted rules from combined table (NA, HA segment)

| **Rule** | **Support** | **Confidence** |
| --- | --- | --- |
| HA8 = M and NA4 = I 🡺 Swine | %20 | %100 |
| HA3 = A and NA4 = N 🡺 Human | %33.33 | %100 |
| HA2 = T and NA1 = W 🡺 Human | %46.66 | %100 |

As shown in Table 10, extracted rules from combined table **cover all the dataset** with **high support and confidence**. The number of these rules is fewer than the number of rules from single tables. So, by using this method, we can classify related datasets with **reliable rules more accurately and faster.**
